# Physical activity and risk of breast cancer, colon cancer, diabetes, ischemic heart disease, and ischemic stroke events: systematic review and dose-response meta-analysis for the Global Burden of Disease Study 2013

**DOI:** 10.1136/bmj.i3857

**Published:** 2016-08-09

**Authors:** Hmwe H Kyu, Victoria F Bachman, Lily T Alexander, John Everett Mumford, Ashkan Afshin, Kara Estep, J Lennert Veerman, Kristen Delwiche, Marissa L Iannarone, Madeline L Moyer, Kelly Cercy, Theo Vos, Christopher J L Murray, Mohammad H Forouzanfar

**Affiliations:** 1 Institute for Health Metrics and Evaluation, University of Washington, 2301 5th Avenue, Suite 600, Seattle, WA 98121, USA; 2 School of Medicine, University of Washington, Seattle, WA 98105, USA; 3 School of Public Health, Faculty of Medicine and Biomedical Sciences, University of Queensland, Herston, QLD 4006, Australia; 4 Geisel School of Medicine, Dartmouth College, Hanover, NH 03755-1404, USA

## Abstract

**Objective** To quantify the dose-response associations between total physical activity and risk of breast cancer, colon cancer, diabetes, ischemic heart disease, and ischemic stroke events.

**Design** Systematic review and Bayesian dose-response meta-analysis.

**Data sources** PubMed and Embase from 1980 to 27 February 2016, and references from relevant systematic reviews. Data from the Study on Global AGEing and Adult Health conducted in China, Ghana, India, Mexico, Russia, and South Africa from 2007 to 2010 and the US National Health and Nutrition Examination Surveys from 1999 to 2011 were used to map domain specific physical activity (reported in included studies) to total activity.

**Eligibility criteria for selecting studies** Prospective cohort studies examining the associations between physical activity (any domain) and at least one of the five diseases studied.

**Results** 174 articles were identified: 35 for breast cancer, 19 for colon cancer, 55 for diabetes, 43 for ischemic heart disease, and 26 for ischemic stroke (some articles included multiple outcomes). Although higher levels of total physical activity were significantly associated with lower risk for all outcomes, major gains occurred at lower levels of activity (up to 3000-4000 metabolic equivalent (MET) minutes/week). For example, individuals with a total activity level of 600 MET minutes/week (the minimum recommended level) had a 2% lower risk of diabetes compared with those reporting no physical activity. An increase from 600 to 3600 MET minutes/week reduced the risk by an additional 19%. The same amount of increase yielded much smaller returns at higher levels of activity: an increase of total activity from 9000 to 12 000 MET minutes/week reduced the risk of diabetes by only 0.6%. Compared with insufficiently active individuals (total activity <600 MET minutes/week), the risk reduction for those in the highly active category (≥8000 MET minutes/week) was 14% (relative risk 0.863, 95% uncertainty interval 0.829 to 0.900) for breast cancer; 21% (0.789, 0.735 to 0.850) for colon cancer; 28% (0.722, 0.678 to 0.768) for diabetes; 25% (0.754, 0.704 to 0.809) for ischemic heart disease; and 26% (0.736, 0.659 to 0.811) for ischemic stroke.

**Conclusions** People who achieve total physical activity levels several times higher than the current recommended minimum level have a significant reduction in the risk of the five diseases studied. More studies with detailed quantification of total physical activity will help to find more precise relative risk estimates for different levels of activity.

## Introduction

Although the protective effect of physical activity on various chronic diseases is well studied and supported in the literature, relatively few studies have systematically quantified the dose-response relations between physical activity and chronic disease endpoints. Systematic reviews that examined the dose-response associations between physical activity and breast cancer,^1^ diabetes,^2^ ischemic heart disease,^3^ or any cancer^4^ focused mainly on a single domain such as leisure time physical activity.^1^ ^2^ ^3^ ^4^ As leisure time activity constitutes a relatively small part of total daily activity,^5^ ^6^ ^7^ ^8^ it does not provide an overall picture of physical activity. Physical activity in any domain (recreation, transportation, household chores, and/or occupation) is beneficial for health and recommended by the World Health Organization.^9^ Moreover, consideration of only a single domain ignores the activity undertaken in other domains, which could result in biased estimates.^10^

Whereas it is important to assess total physical activity and its dose-response association with health outcomes, there are several challenges to accomplish this. The measurement and classification of physical activity in individual studies are heterogeneous. There is a lack of consistency across studies in terms of the domains being assessed (recreational, household, transport related, occupational, or total activity) and the metric of measurement (for example, quantitative metric such as hours a week and qualitative metric such as inactive versus highly active). The categorization of activity levels also varies widely across studies. As a result, most meta-analyses did not assess dose-response associations but compared only the least active with the most active individuals. A limitation of this approach is that being the most active in recreational activities might not be comparable with being the most active in occupational activities, indicating a need for standardization of physical activity across studies.

WHO recommends at least 600 metabolic equivalent (MET) minutes of total activity (irrespective of domains) per week for health benefits; this would be, for example, about 150 minutes/week of brisk walking or 75 minutes/week of running.^11^ Despite the well established causal relations between physical activity and chronic diseases, including breast cancer, colon cancer, diabetes, ischemic heart disease, and ischemic stroke,^10^ ^12^ ^13^ knowledge is limited as to how much the risk decreases with an increase in the amount of total activity. To date, no study has quanitified, in a dose-response fashion, the amount of total physical activity required to lower the risk of these diseases using all available data sources. We conducted a systematic review to estimate the association between total physical activity, standardized as a continuous scale (MET minutes/week) and the five outcomes (breast cancer, colon cancer, diabetes, ischemic heart disease, and ischemic stroke) for the Global Burden of Disease (GBD) 2013 study.

## Methods

### Literature search

We conducted this systematic review following PRISMA and MOOSE guidelines.^14^ ^15^ This review was performed following the methods documented in a systematic review protocol (appendix 1). We searched PubMed and Embase from 1980 to 30 September 2014 and updated the search up to 27 February 2016 for studies that examined the association between physical activity and the risks of one of the five outcomes (breast cancer, colon cancer, diabetes, ischemic heart disease, ischemic stroke). We restricted the search to English language publications and studies in humans. Appendix 2 shows the search strategies. We also reviewed the reference list of included studies in previous systematic reviews of these outcomes.

### Study selection

Prospective cohort studies that assessed physical activity as the exposure variable (total activity or domain specific activity that allowed conversion to total activity) and at least one of the five chosen diseases as an outcome and provided risk estimates (relative risk, hazard ratio, or odds ratio) with confidence intervals or standard errors (or sufficient data to calculate them) were eligible for inclusion. Disagreements on eligibility were resolved by consensus. We included only prospective cohort studies to minimize recall and selection biases that are common in case-control studies. For studies that categorized physical activity qualitatively, they also had to report number of individuals or person years in each activity category. If multiple studies reported on the same dataset and study period, we included the one with a more detailed report of physical activity and better control of confounding variables. We considered the following variables to be the main potential confounders: age (all outcomes), sex (ischemic heart disease and ischemic stroke), family history of the outcome of interest (all outcomes), estrogen and progesterone exposure (breast cancer), and lifestyle factors (all outcomes).

### Data extraction

Four authors (VB, MM, JEM, and HHK) independently extracted data using a standardized data extraction form. HHK and LTA independently checked the data. The following variables were extracted from each included study: author, year of publication, study location, duration of follow-up, sex, age at baseline, type of physical activity (leisure time/recreational, domestic, occupational and/or transport related activity), measurement method of physical activity, category, duration, frequency and/or intensity of physical activity, dose of physical activity (for example, minutes a week, MET hours/week), sample size, response rate, number of cases and participants in each category, and risk estimates with corresponding confidence intervals (age/sex adjusted and multivariate adjusted) for each activity category.

### Assessment of quality of included studies

We used the Newcastle-Ottawa scale (NOS)^16^ to assess the quality of included studies in representativeness of the cohort, whether the non-exposed participants were drawn from the same population as the exposed, ascertainment of exposure, whether the outcome of interest was absent at the start of study, comparability of the exposed and unexposed (that is, adjustment for potential confounding variables), ascertainment of the outcome, whether the length of the follow-up was long enough (at least five years) for the outcome to occur, and the completeness of the follow-up (loss to follow-up <20%). A maximum score of 2 can be awarded for comparability and a maximum score of 1 can be given for each of the remaining items. A study can have a maximum possible quality score of 9.

### Preparation of data for dose-response meta-analysis

In preparation for the dose-response meta-analysis, we standardized domain specific physical activity measures to total MET minutes of activity a week. Domain specific physical activity measures refer to the activity undertaken in different domains of life (for example, activity in the domain of leisure time, activity in the domain of work, and activity in the domain of transportation). MET represents the ratio of the working metabolic rate to the resting metabolic rate. One MET is defined as the amount of oxygen consumed while a person is sitting quietly and is about 3.5 mL O_2_/kg body weight/min.^17^ To map domain specific activity to total activity, we carried out log-log ordinary least squares regression to determine the association between total and domain specific activities using data from the Study on Global Ageing and Adult Health (SAGE) conducted in six countries (China, Ghana, India, Mexico, Russia, and South Africa) from 2007 to 2010 and additional data from the US National Health and Nutrition Examination Surveys (NHANES) from 1999 to 2011.^18^ ^19^ ^20^ ^21^ ^22^ ^23^ ^24^ ^25^ ^26^ ^27^ ^28^ ^29^ ^30^ Both SAGE and NHANES are nationally representative surveys that measured activity in recreation, transportation, and occupation separately with the Global Physical Activity Questionnaire (GPAQ), allowing mapping from domain specific metrics to total activity across all domains. As domestic physical activity is not explicitly captured in these surveys, we applied a correction factor for domestic activity for women in developing countries, for whom we found significantly lower activity levels when assessed with the GPAQ compared with the International Physical Activity Questionnaire (IPAQ), which captured all activity (including domestic activity). We did not adjust for domestic activity for men and women in developed countries and for men in developing countries as we did not find any significant difference in the total activity level between surveys that used the GPAQ and those that used the IPAQ. The regression coefficients were applied to the MET minutes/week cut offs for the domain specific activity categories in included studies that reported MET minutes/week or were converted directly to MET minutes/week, resulting in estimated total weekly activity for each relative risk level. For studies that measured physical activity quantitatively, but not in METs, we calculated MET minutes/week based on the reported duration and intensity of activity, assigning 4 METs to the time spent in moderate intensity activities and 8 METs to vigorous activities, as suggested by WHO.^11^

For studies that assessed physical activity qualitatively (such as low, moderate, and high) with no additional information on duration and intensity of activity, we calculated the centiles of the activity distribution and mapped them to the GBD 2013 exposure distribution in MET minutes/week (which are specific for country, year, age, and sex) to generate estimates of total physical activity. More specifically, we assigned a centile cut off to each reported activity category based on the percentage of the study population falling into that activity category. A centile value was also assigned to each of the GBD 2013 MET minute cut points (600, 3999, and 8000 MET minutes/week) based on the GBD 2013 estimated exposure distributions. The method used to estimate physical activity exposure in GBD 2013 has been reported in detail elsewhere.^13^ Within each activity category, exposure was assumed to be uniformly distributed. Centiles from the studies were mapped to those assigned to the GBD 2013 distribution of physical activity exposure, and the corresponding MET minute value was assigned as the cut off for each category reported by a study.

### Data analysis

We used Dismod-MR 2.0,^31^ GBD’s bayesian meta-regression tool, to pool effect sizes from included studies and generate a dose-response total physical activity curve for each of the five outcomes. The tool enabled us to incorporate random effects across studies and include data with different activity ranges and variation in categorization across studies. The equations and explanations are presented in appendix 3. The dose-response meta-analysis/meta-regression was run separately for each of the five outcomes. We included study covariates indicating whether or not the MET minutes were estimated with the GBD 2013 exposure data; whether a study reported relative risk, odds ratio, or hazard ratio; and a sex covariate (men, women, or both sexes) (sex was not included as a covariate in the meta-analysis for breast cancer as we focused only on breast cancer among women). For ischemic stroke, because the number of studies identified was small (n=13), we also included 13 studies that reported the association between physical activity and total stroke and included a study level covariate indicating whether the outcome was ischemic stroke or total stroke. Ischemic stroke was set as the reference category, which allowed Dismod-MR2 to adjust the relative risk for total stroke to the reference level.

### Publication bias

Publication bias was assessed with funnel plots and the Egger test.^32^ To assess the influence of any possible publication bias we used sensitivity analyses with the trim and fill method, which identifies potentially missing studies and corrects for funnel plot asymmetry.^33^

### Sensitivity analysis

We assessed the impact of the quality of studies on the findings by sensitivity analyses including only higher quality studies. To evaluate the influence of imprecise measurement of exposure, we also conducted sensitivity analyses separately for studies that measured physical activity quantitatively and those that assessed it qualitatively, for which MET minutes were estimated with the GBD 2013 exposure data.

### Patient involvement

No patients were involved in setting the research question or the outcome measures, nor were they involved in developing plans for design or implementation of the study. No patients were asked to advise on interpretation or writing up of results. There are no plans to disseminate the results of the research to study participants or the relevant patient community.

## Results

Our literature search identified a total of 11 166 citations (fig 1). After removal of the duplicate citations, 6965 studies remained for title and abstract screening, of which 223 articles were potentially relevant for full text review. We excluded 26 articles that used the same dataset as other included studies. An additional 23 articles with insufficient data (such as lack of information on the number of individuals or person years in each activity category, which was needed to convert from qualitative activity levels to METs) were also excluded. This left a total of 174 studies (149 184 285 total person years of follow-up) to include in our bayesian dose-response meta-analysis: 35 studies for breast cancer (50 949 108 person years), 19 for colon cancer (53 929 648 person years), 55 for diabetes (14 051 132 person years), 43 for ischemic heart disease (16 583 824 person years), and 26 for ischemic stroke (13 670 573 person years) (the number of included studies for each outcome does not sum up to 174 because some studies included multiple outcomes). Characteristics of included studies for each outcome are shown in the tables A-E in appendix 4.

**Figure f1:**
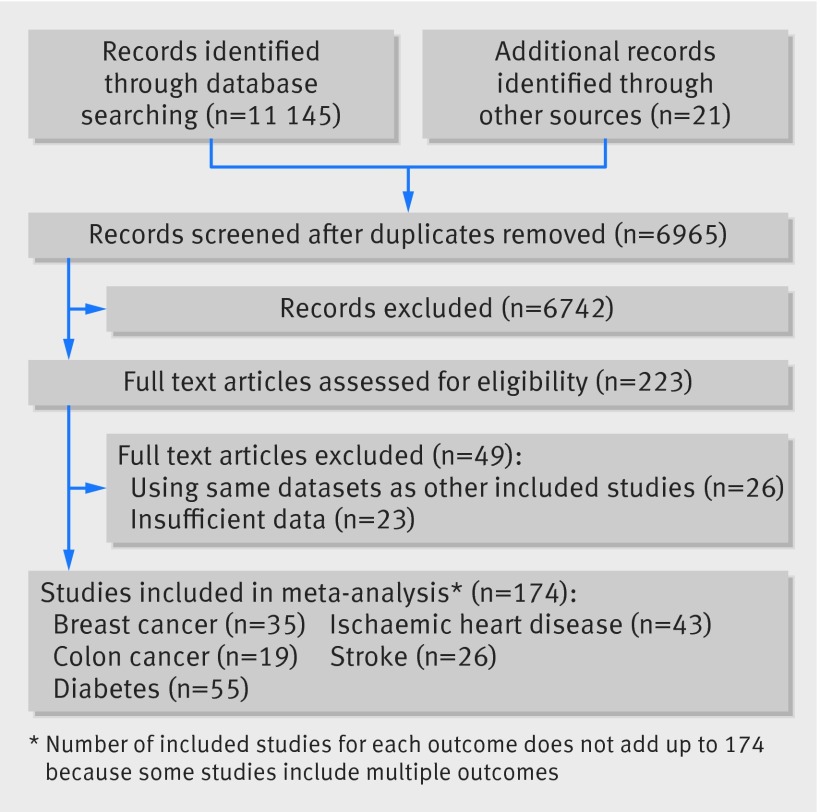
**Fig 1** Flow chart of selecting studies for inclusion in dose-response meta-analysis of effect of physical activity on five diseases

### Continuous dose-response relations

The continuous risk curves for each of the five outcomes are shown in figures 2-6[Fig f2 f3 f4 f5 f6]. Higher levels of total physical activity were associated with lower risk of all outcomes. Major gains occurred at lower levels of activity, and the decrease in risk was minimal at levels higher than 3000-4000 MET minutes/week. (A person can achieve 3000 MET minutes/week by incorporating different types of physical activity into the daily routine—for example, climbing stairs 10 minutes, vacuuming 15 minutes, gardening 20 minutes, running 20 minutes, and walking or cycling for transportation 25 minutes on a daily basis would together achieve about 3000 MET minutes a week). This pattern was most prominent for ischemic heart disease and diabetes and least prominent for breast cancer (fig 7; table F in appendix 4). For example, individuals with a total activity level of 600 MET minutes/week (the minimum level recommended by WHO) had a 2% lower risk of diabetes compared with those reporting no physical activity. An increase from 600 to 3600 MET minutes/week reduced the risk by an additional 19%. The same amount of increase yielded much smaller returns at higher levels of activity: an increase of total activity from 9000 to 12 000 MET minutes/week reduced the risk of diabetes by only 0.6% (table F in appendix 4). The corresponding risk reduction for breast cancer was 1% for an increase in total physical activity from 0 to 600 MET minutes/week (not significant), an additional 4% reduction in risk for an increase from 600 to 3600 MET minutes/week, and a 2% reduction in risk for an increase in total activity from 9000 to 12 000 MET minutes/week (table F in appendix 4).

**Figure f2:**
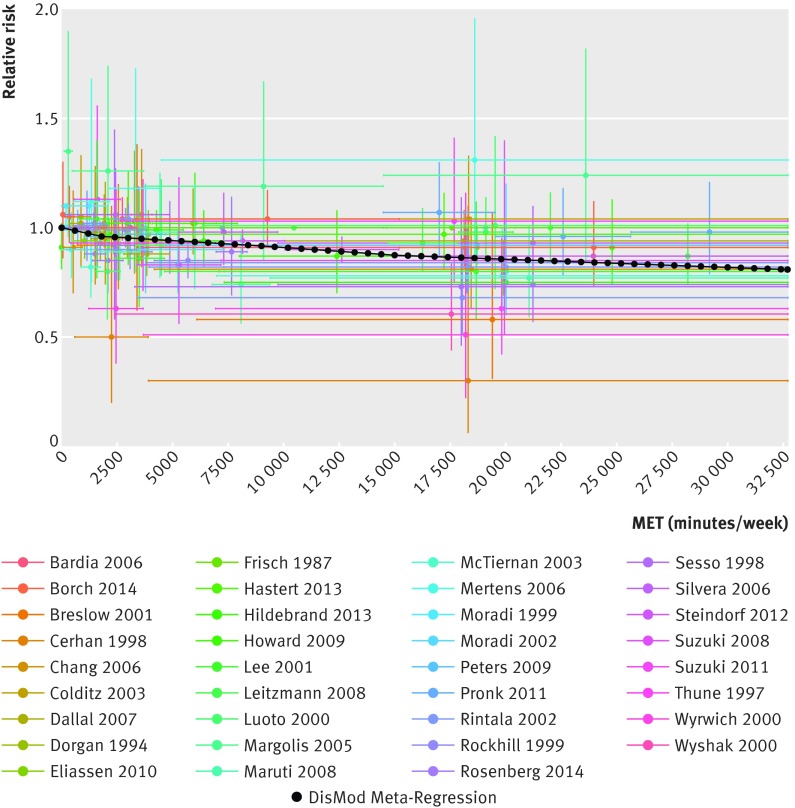
**Fig 2** Continuous risk curve for association between physical activity and breast cancer. For each datapoint in included studies, which are represented by different colors, length of horizontal bar refers to total physical activity interval in MET-minutes/week and vertical bar refers to confidence interval of relative risks

**Figure f3:**
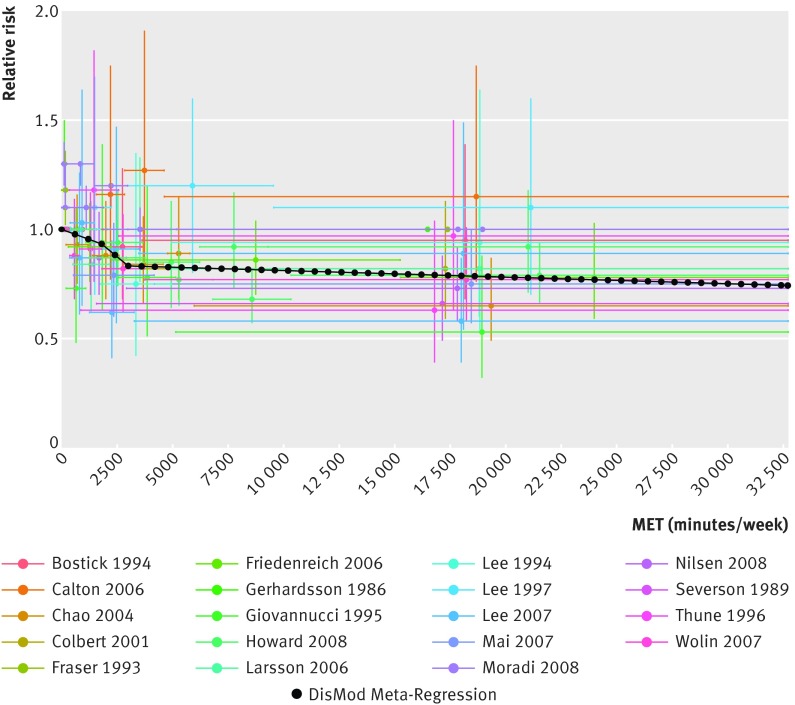
**Fig 3** Continuous risk curve for association between physical activity and colon cancer. For each datapoint in included studies, which are represented by different colors, length of horizontal bar refers to total physical activity interval in MET-minutes/week and vertical bar refers to confidence interval of relative risks

**Figure f4:**
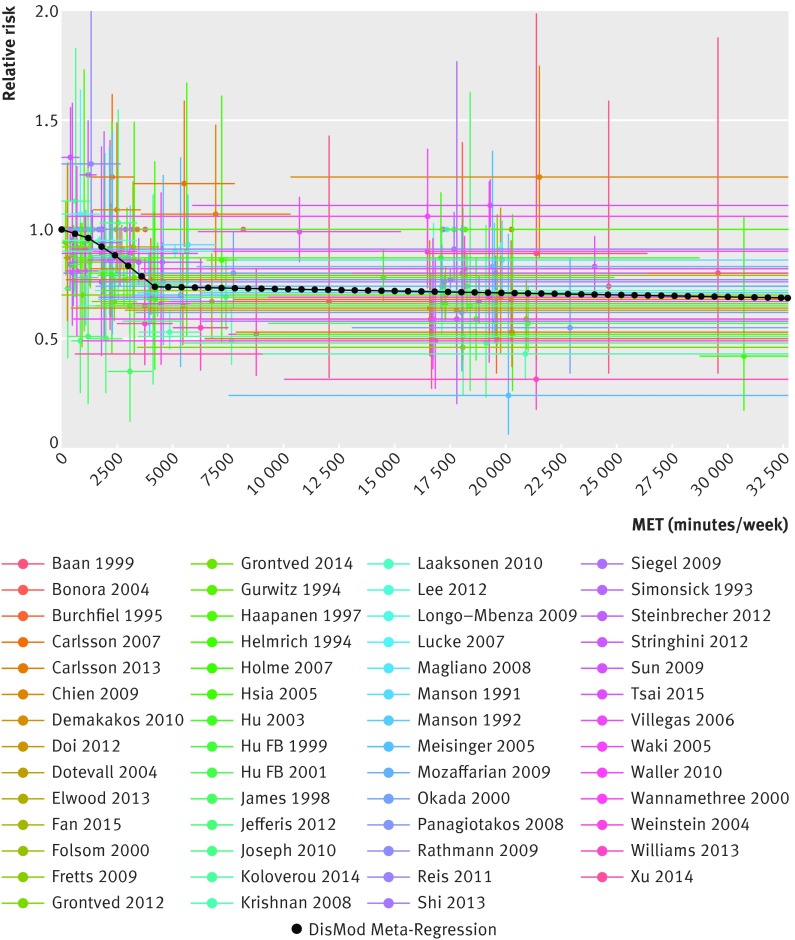
**Fig 4** Continuous risk curve for association between physical activity and diabetes. For each datapoint in included studies, which are represented by different colors, length of horizontal bar refers to total physical activity interval in MET-minutes/week and vertical bar refers to confidence interval of relative risks

**Figure f5:**
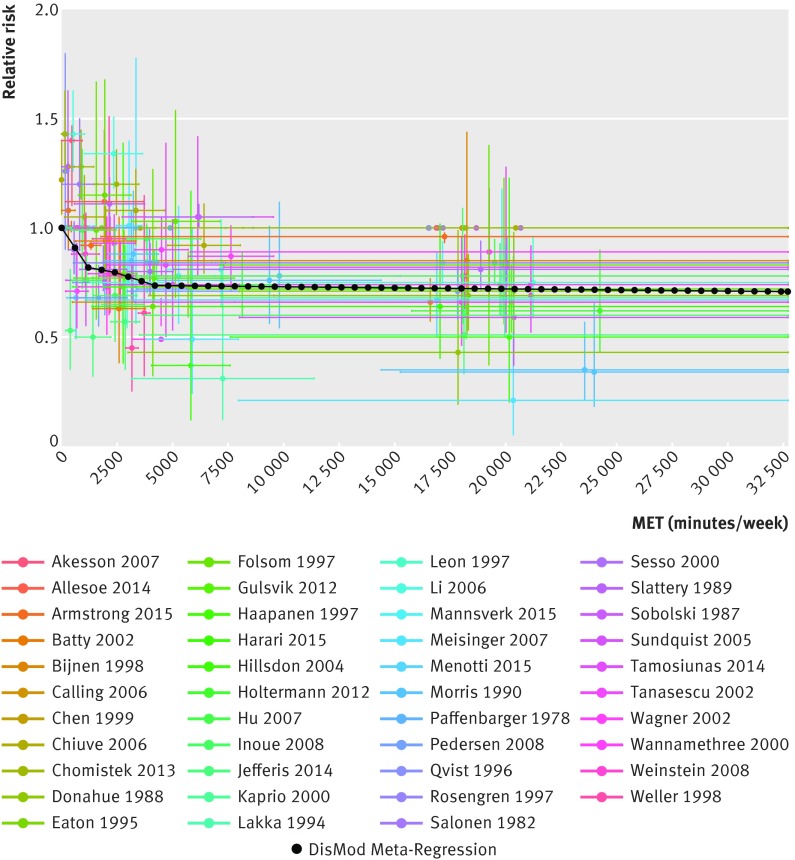
**Fig 5** Continuous risk curve for association between physical activity and ischemic heart disease. For each datapoint in included studies, which are represented by different colors, length of horizontal bar refers to total physical activity interval in MET-minutes/week and vertical bar refers to confidence interval of relative risks

**Figure f6:**
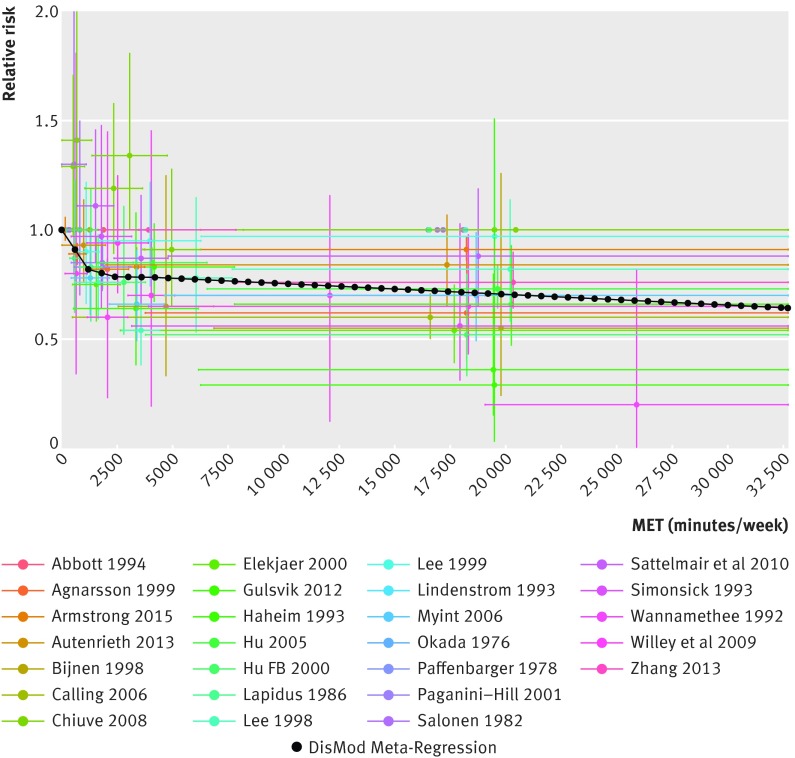
**Fig 6** Continuous risk curve for association between physical activity and ischemic stroke. For each datapoint in included studies, which are represented by different colors, length of horizontal bar refers to total physical activity interval in MET-minutes/week and vertical bar refers to confidence interval of relative risks

**Figure f7:**
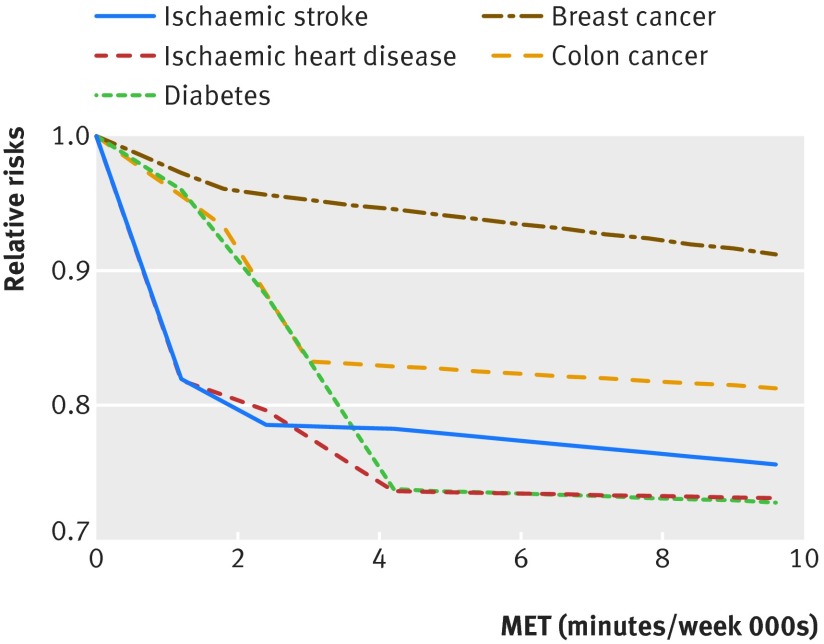
**Fig 7** Continuous risk curves for association between physical activity and breast cancer, colon cancer, diabetes, ischemic heart disease, and ischemic stroke

### Categorical dose-response relations

Table 1 shows the relative risks and uncertainty intervals for the associations between physical activity and the five outcomes (uncertainty intervals are a range of values that are likely to include the correct risk estimate for the association between physical activity and each health outcome).

**Table 1 tbl1:** Categorical dose-response relations between physical activity and breast cancer, colon cancer, diabetes, ischemic heart disease, and ischemic stroke

Physical activity in MET minutes/week	Pooled relative risk (95% uncertainty interval)				
	Breast cancer	Colon cancer	Diabetes	Ischemic heart disease	Ischemic stroke
<600	Reference	Reference	Reference	Reference	Reference
600-3999	0.967 (0.937 to 0.998)	0.903 (0.851 to 0.952)	0.857 (0.816 to 0.902)	0.837 (0.791 to 0.886)	0.843 (0.779 to 0.918)
4000-7999	0.941 (0.904 to 0.981)	0.833 (0.771 to 0.896)	0.748 (0.701 to 0.799)	0.769 (0.698 to 0.838)	0.810 (0.690 to 0.937)
≥8000	0.863 (0.829 to 0.900)	0.789 (0.735 to 0.850)	0.722 (0.678 to 0.768)	0.754 (0.704 to 0.809)	0.736 (0.659 to 0.811)
Heterogeneity	0.002 (0.0001 to 0.008)	0.005 (0.0003 to 0.018)	0.050 (0.040 to 0.070)	0.042 (0.025 to 0.061)	0.016 (0.001 to 0.043)

Compared with insufficiently active women (reporting less than 600 MET minutes/week of total physical activity), the risk of breast cancer among those in the low active (600-3999 MET minutes), moderately active (4000-7999 MET minutes), and highly active (≥8000 MET minutes) categories was reduced by 3%, 6%, and 14%, respectively. Compared with insufficiently active individuals (both men and women), the risk of colon cancer among those in the low active, moderately active, and highly active categories was reduced by 10%, 17%, and 21%, respectively. The corresponding reductions in risk were 14%, 25%, and 28% for diabetes, 16%, 23%, and 25% for ischemic heart disease, and 16%, 19%, and 26% for ischemic stroke, respectively.

### Assessment of risk of bias and publication bias

Tables A-E in appendix 4 show the quality assessment scores for each study. The scores varied from a maximum of 9 to a minimum of 3. Sensitivity analyses in which we excluded studies with a score <7 showed similar results, except for diabetes (table 2). The strength of association between total physical activity and diabetes was weaker for moderately active and highly active categories when we restricted the analysis to higher quality studies (table 2).

**Table 2 tbl2:** Categorical dose-response relations between physical activity and breast cancer, colon cancer, diabetes, ischemic heart disease, and ischemic stroke in sensitivity analysis excluding studies with quality score <7 on Newcastle-Ottawa scale

Physical activity in MET minutes/week	Pooled relative risk (95% uncertainty interval)				
	Breast cancer	Colon cancer	Diabetes	Ischemic heart disease	Ischemic stroke
<600	Reference	Reference	Reference	Reference	Reference
600-3999	0.974 (0.937 to 1.015)	0.887 (0.799 to 0.980)	0.890 (0.808 to 0.968)	0.860 (0.796 to 0.926)	0.862 (0.780 to 0.954)
4000-7999	0.950 (0.905 to 0.998)	0.814 (0.708 to 0.920)	0.827 (0.724 to 0.930)	0.786 (0.696 to 0.883)	0.839 (0.697 to 0.981)
≥8000	0.877 (0.834 to 0.923)	0.795 (0.702 to 0.898)	0.798 (0.702 to 0.891)	0.782 (0.723 to 0.842)	0.802 (0.696 to 0.898)
Heterogeneity	0.003 (0.0001 to 0.009)	0.009 (0.0004 to 0.031)	0.055 (0.038 to 0.073)	0.045 (0.022 to 0.071)	0.019 (0.001 to 0.056)

The study covariate indicating whether or not the MET minutes were estimated with the GBD 2013 exposure data was not significant for all outcomes except for breast cancer. Sensitivity analyses for breast cancer studies did not show a significant difference between those that assessed physical activity quantitatively and those that assessed it qualitatively, though the latter tended to show slightly stronger associations (table G, appendix 4).

Egger’s test^27^ for publication bias was significant (P<0.05) for diabetes, ischemic heart disease, and ischemic stroke. Sensitivity analyses in which we included the missing studies identified through the trim and fill method showed similar results (data not shown). There was no significant evidence of publication bias for breast cancer and colon cancer.

## Discussion

This is the first meta-analysis to quantify the dose-response association between total physical activity across all domains and the risk of five chronic diseases. Using data from 174 cohort studies, we estimated relative risks of diseases for each dose of total physical activity in MET minutes/week. The results of our meta-analysis showed that higher levels of total physical activity were significantly associated with lower risk for all outcomes: major gains occurred at lower levels of activity and there were diminishing returns at levels higher than 3000-4000 MET minutes/week. There was no evidence that the findings differed between studies with a higher or lower risk of bias for activity levels where most health gains occurred.

### Comparison with previous work

The findings of this study extend previous meta-analyses in several important ways. First, this study included a lot more studies than previous quantitative dose-response meta-analyses. For example, we included 55, 35, and 43 cohort studies on diabetes, breast cancer, and ischemic heart disease, respectively, in our study compared with 16 studies on diabetes,^2^ 13 studies on breast cancer,^1^ and nine studies on ischemic heart disease^3^ included in previous dose-response meta-analyses. No studies have systematically quantified the dose-response associations between physical activity and the remaining outcomes (colon cancer and ischemic stroke). Second, in contrast with previous dose-response meta-analyses that focused on a single domain, such as leisure time activity,^1^ ^3^ ^4^ we quantified total physical activity across all domains and thus provide an overall picture. The quantification of total activity was made possible by the use of nationally representative surveys, including SAGE and NHANES, that cover activity in different domains, making it possible to map domain specific activity to total activity. Third, the use of Dismod-MR 2.0, GBD’s bayesian meta-regression tool, enabled us to include data with different activity ranges and variation in categorization across studies. This has not been possible previously, and, as a result, most meta-analyses provided pooled estimates comparing the most active with the least active individuals. As being most active in recreational activities might not be comparable with being the most active in occupational activities, this makes it difficult to interpret the resulting pooled estimates. In the present study, we enhanced the comparability across studies by standardizing different measures of physical activity into total activity in MET minutes/week.

Of the previous meta-analyses examining the dose-response associations of leisure time physical activity with breast cancer,^1^ diabetes,^2^ and ischemic heart disease,^3^ the breast cancer study reported a linear association whereas the latter two found smaller returns at higher levels of activity. Consistent with the latter studies, we found diminishing returns at higher levels of total physical activity for diabetes and ischemic heart disease. We also found a similar pattern of associations for breast cancer, colon cancer, and ischemic stroke.

### Implications of findings

Our findings have several important implications. They suggest that total physical activity needs to be several times higher than the current recommended minimum level of 600 MET minutes/week to achieve larger reductions in risks of breast cancer, colon cancer, diabetes, ischemic heart disease, and ischemic stroke. Focusing on a particular domain such as leisure time physical activity, which represents only a small fraction of total activity, as was done by most studies, restricts the scope of applicability of the findings in the real world by limiting the opportunity of increasing activity in different domains in daily life (such as being more physically active at work, engaging more in domestic activities such as housework and gardening, and/or engaging in active transportation such as walking and cycling). Taking into account all domains of physical activity increases opportunities for promoting physical activity. Further work with studies with more detailed quantification of total physical activity is warranted to provide more precise estimates for different levels of physical activity. Finally, the methodological innovation of this study could be applicable to other systematic reviews (in different specialties) meta-analyzing studies with variation in categorizations of exposure.

### Strengths and limitations of the study

Our study included much larger data coverage than previous dose response meta-analyses on breast cancer, diabetes, and ischemic heart disease. It is the first to examine the dose-response associations between physical activity and colon cancer and ischemic stroke. Other strengths included the quantification of total physical activity instead of restricting it to a single domain of activity, the enhancement of comparability across studies through standardization of the measure of physical activity, and the ability to include data with various categorizations of physical activity while incorporating random effects across studies.

It does, however, have some limitations. First, we might have missed articles as a result of restricting our search to two databases and studies published in English. Previous systematic reviews without language restrictions found none or few non-English studies.^1^ ^2^ Available evidence^34^ suggests that a combination of Embase and Medline (a subset of the PubMed database)^35^ yields a coverage of 97.5%. In addition to searching Embase and PubMed, we manually searched the reference list of relevant articles so we believe that the percentage of missing studies is likely to be small and have little impact on the findings. Second, the effect of physical activity on the outcomes might be influenced by unmeasured factors and/or effect modifiers. Because our analysis relied on the data reported by cohort studies, we could not account for the potential for residual confounding or effect modification. Third, results of the test of publication bias were significant for diabetes, ischemic heart disease, and ischemic stroke. The inclusion of potentially missing studies (imputed based on funnel plot asymmetry) in our sensitivity analyses, however, showed similar results. Fourth, the dose-response meta-analysis included studies that measured physical activity qualitatively, which were mapped to the GBD 2013 exposure distribution to generate estimates of total physical activity. This imprecise measurement of exposure could lead to regression dilution bias, resulting in underestimation of the relative risks. Sensitivity analyses, however, showed no significant difference in the pooled relative risks between studies that used quantitative versus qualitative exposure metrics. Fifth, we focused on volume (a combination of intensity, frequency, and duration) because of its wide use in practice and the availability of the data. This ignores the role that intensity plays in the dose of physical activity. Future investigations could extend our findings by examining the impact of different intensity compositions of total physical activity on risks of disease. Sixth, in this study, we calculated pooled relative risks for the associations between different doses of total physical activity and five chronic diseases. Although it would be ideal to provide both relative and absolute risk reduction to portray a more complete picture, many included studies reported only relative risks and did not provide the risk of events in the exposed and unexposed groups separately or the information to calculate them to compute the absolute risk. Seventh, there could be changes in MET hours/week over time. As we relied on the physical activity reported by studies, we did not have supplementary data to correct for that. Eighth, we used NHANES and SAGE surveys to map from domain specific metrics to total activity across all domains. These surveys did not capture European countries (other than Russia). Physical activity patterns might vary across locations, even among developed countries, and our mapping might be less precise for countries that were not included in NHANES and SAGE surveys. Finally, we chose the upper bound of the highest activity category based on the 99th centile of population based microdata, which is large and could cause underestimation of the effect size for that category, but the continuous risk curves reached a plateau at lower levels of activity, suggesting that shifting studies to the left would not make a big difference.

### Conclusions

In conclusion, the findings of this study showed that a higher level of total physical activity is strongly associated with a lower risk of breast cancer, colon cancer, diabetes, ischemic heart disease, and ischemic stroke, with most health gains occurring at a total activity level of 3000-4000 MET minutes/week. Results suggest that total physical activity needs to be several times higher than the recommended minimum level of 600 MET minutes/week for larger reductions in the risk of these diseases. With population ageing, and an increasing number of cardiovascular and diabetes deaths since 1990,^36^ greater attention and investments in interventions to promote physical activity in the general public is required. More studies using the detailed quantification of total physical activity will help to find a more precise estimate for different levels of physical activity.

**What is already known on this topic**

Many cohort studies and meta-analyses have shown the health benefits of physical activity, resulting in WHO recommending a minimum total physical activity level (irrespective of domains including leisure time, household, occupation, and/or transportation) of 600 MET minutes a week, but the upper limit of total activity required is not known
Meta-analyses that examined the dose-response associations between physical activity and chronic diseases focused mainly on a single domain of activity such as leisure time physical activity, which constitutes a relatively small part of total daily activity, and thus the relation between total physical activity and chronic diseases has not been well characterized

**What this study adds**

This dose-response meta-analysis focused on total physical activity across different domains of life (leisure time, occupation, domestic, transportation) and included about three to five times more prospective cohort studies than previous dose-response meta-analyses that focused on a single domain of activity only
The continuous risk curves for the associations between total physical activity and breast cancer, colon cancer, diabetes, ischemic heart disease, and ischemic stroke show that although the risks of these diseases decrease with increasing level of total activity, most health gains occur at relatively lower levels of activity (up to 3000-4000 MET minutes/week), with diminishing returns at higher levels of activity

## Web Extra

Extra material supplied by the author

**Appendix 1:** Protocol

**Appendix 2:** Search strategies

**Appendix 3:** Equations

## Notes

We thank Belén Zapata Diomedi for her helpful comments on the manuscript; Emmanuela Gakidou for her valuable contribution to the production of the manuscript; Erica L Slepak and Yesenia Roman for their help with the citations of included studies; and Brad Bell for his valuable insights about the DisMod-MR software and the documentation.

Contributors: HHK, TV, CJLM, and MHF conceived, designed, and supervised the study. HHK and MLI developed and implemented the literature search strategy. HHK, VFB, MLM, MLI, JEM, KC, TV, and MHF acquired the data, including review of literature search results and data abstraction. HHK, VFB, LTA, KD, JLV, TV, CJLM, and MHF analysed and interpreted the data. HHK, VFB, LTA, AA, TV, and MHF drafted the manuscript, which was critically revised for important intellectual content by all authors. HHK and MHF carried out statistical analysis. KE provided administrative, technical, and material support. TV, CJLM, and MHF are joint senior authors. HHK and MHF are guarantors.

Ethical approval: Not required.

Data sharing: The full dataset is available from the corresponding author.

Transparency: The lead author affirms that the manuscript is an honest, accurate, and transparent account of the study being reported; that no important aspects of the study have been omitted; and that any discrepancies from the study as planned have been explained.
