# Supplementary material for: Physical activity and risk of breast cancer, colon cancer, diabetes, ischemic heart disease, and ischemic stroke events: systematic review and dose-response meta-analysis for the Global Burden of Disease Study 2013
Source: BMJ. 2016 Aug 9;354:i3857. doi: 10.1136/bmj.i3857 (PMC4979358; doi:10.1136/bmj.i3857)
Supplement: Supplementary file 1 — Appendix 1: Protocol [file kyuh031211.ww1_default.pdf]

Appendix 1: Systematic Review Protocol [posted as supplied by author]

Title

Physical Activity and Risks of Breast Cancer, Colon Cancer, Diabetes, Ischemic Heart Disease and Ischemic Stroke Events: A Systematic Review and Dose-Response Meta-Analysis for the Global Burden of Disease Study 2013

Reviewers

Hmwe Kyu, Victoria Bachman, Theo Vos, Mohammad Forouzanfar

Review objective

To quantify the dose-response relationship between total physical activity and risk of breast cancer, colon cancer, diabetes, ischemic heart disease and ischemic stroke events using all available data.

Exposure(s)

The exposure will be total physical activity or activity in any domain (recreational, household, transport-related, occupational) that allows conversion to total activity. Physical activity may be defined by different methods. The gold standard and preferred method is to measure activity in all domains by the Global Physical Activity Questionnaire (GPAQ) or International Physical Activity Questionnaire (IPAQ) and report for each activity category. If a study did not define physical activity in terms of metabolic equivalent of task (MET) but reported type, duration and intensity of it, we will convert it to MET-minutes, by assigning 4 METs to the time spent in moderate intensity activities and 8 METs to vigorous activities respectively as suggested by the World Health Organization [1]. If participants were categorized qualitatively (e.g., inactive versus active), we will extract the proportion of sample in each group as the percentile range of

activity level. We will then map the categories of activity reported in the paper to the 4 levels of activity categories in the Global Burden of Disease Study (GBD) 2013 exposure data, using country, year, sex and age of participants. Country-year-age-sex-specific levels of activity are measured in the GBD as part of estimating the distribution of exposure to different levels of physical activity. We will assume the 99th percentile of total activity in available micro data from population based surveys as the maximum level of activity.

Outcome(s)

Breast cancer, colon cancer, diabetes, ischemic heart disease and/or ischemic stroke events including newly diagnosed cases and mortality.

Participants/population

Studies reporting findings of the associations between physical activity (any domain) and risks of breast cancer, colon cancer, diabetes, ischemic heart disease and/or ischemic stroke events among adults 15 years and above.

Inclusion criteria

Prospective observational studies that examined the association between physical activity and breast cancer, colon cancer, diabetes, ischemic heart disease and/or ischemic stroke events, and provided risk estimates (relative risk, hazard ratio or odds ratio) with confidence intervals or standard errors, or data to calculate them, are eligible for inclusion. For studies that categorized physical activity qualitatively, the number of individuals or person years in each activity category must also be reported.

Exclusion criteria:

- Based on the study design (e.g., cross-sectional, case-control)
- Based on the population (e.g., people with the underlying chronic diseases)

- Based on the type of articles (e.g., reviews, commentaries, letters, and duplicate publications from the same study). If multiple studies reported on the same dataset and study period, we will include the one with a more detailed report of physical activity and a better control of confounding variables.

Search strategy

We will search PubMed and EMBASE from 1980 to October 15, 2013 for studies that examined the association between physical activity and the risks of one of the five outcomes (breast cancer, colon cancer, diabetes, ischemic heart disease and ischemic stroke), restricting to English-language publications and human studies. A systematic search strategy will be developed using key words related to the outcomes being investigated. An updated search will be conducted immediately prior to data synthesis. We will also review the reference list of included studies in previous systematic reviews of these outcomes.

Search Query for EMBASE and PubMed

**EMBASE**	**Search strategies**
Breast cancer	'physical activity':ab,ti and 'breast cancer':ab,ti and ([article]/lim or [article in press]/lim) and [humans]/lim and [english]/lim and [embase]/lim not [1-10-2014]/sd and [1980-2015]/py
Colon cancer	'physical activity':ab,ti AND 'colon cancer':ab,ti AND [humans]/lim AND [english]/lim AND [embase]/lim NOT [1-10-2014]/sd AND [1980-2015]/py
Diabetes	'physical activity':ab,ti AND 'type 2 diabetes':ab,ti AND [1980-2015]/py AND ([article]/lim OR [article in press]/lim) AND [humans]/lim AND [english]/lim AND [embase]/lim NOT [1-10-2014]/sd
Diabetes	'physical activity':ab,ti AND 'noninsulin dependent diabetes mellitus':ab,ti AND [humans]/lim AND [english]/lim AND [embase]/lim NOT [1-10-2014]/sd AND [1980-2015]/py
Diabetes	'physical activity':ab,ti AND 'niddm':ab,ti AND [humans]/lim AND [english]/lim AND [embase]/lim NOT [1-10-2014]/sd AND [1980-2015]/py
Ischemic heart disease	'physical activity':ab,ti AND 'ischemic heart disease':ab,ti AND [humans]/lim AND [english]/lim AND [embase]/lim NOT [1-10-

	2014]/sd AND [1980-2015]/py
Ischemic heart disease	'physical activity':ab,ti AND 'ischaemic heart disease':ab,ti AND [humans]/lim AND [english]/lim AND [embase]/lim NOT [1-10-2014]/sd AND [1980-2015]/py
Ischemic heart disease	'physical activity':ab,ti AND 'coronary heart disease':ab,ti AND [humans]/lim AND [english]/lim AND [embase]/lim NOT [1-10-2014]/sd AND [1980-2015]/py
Ischemic stroke	'physical activity':ab,ti AND 'ischemic stroke':ab,ti AND [humans]/lim AND [english]/lim AND [embase]/lim NOT [1-10-2014]/sd AND [1980-2015]/py
Ischemic stroke	'physical activity':ab,ti AND 'ischaemic stroke':ab,ti AND [humans]/lim AND [english]/lim AND [embase]/lim NOT [1-10-2014]/sd AND [1980-2015]/py
**PubMed**	
Breast cancer	physical activity[Title/Abstract] AND breast cancer [Title/Abstract] AND "humans"[MeSH Terms] AND English[lang] AND ("1980/01/01"[PDAT] : "2014/09/30"[PDAT])
Colon cancer	physical activity[Title/Abstract] AND colon cancer [Title/Abstract] AND "humans"[MeSH Terms] AND English[lang] AND ("1980/01/01"[PDAT] : "2014/09/30"[PDAT])
Diabetes	physical activity[Title/Abstract] AND type 2 diabetes[Title/Abstract] AND "humans"[MeSH Terms] AND English[lang] AND ("1980/01/01"[PDAT] : "2014/09/30"[PDAT])
Diabetes	physical activity[Title/Abstract] AND noninsulin dependent diabetes mellitus [Title/Abstract] AND "humans"[MeSH Terms] AND English[lang] AND ("1980/01/01"[PDAT] : "2014/09/30"[PDAT])
Diabetes	physical activity[Title/Abstract] AND niddm[Title/Abstract] AND "humans"[MeSH Terms] AND English[lang] AND ("1980/01/01"[PDAT] : "2014/09/30"[PDAT])
Ischemic heart disease	physical activity[Title/Abstract] AND ischemic heart disease [Title/Abstract] AND "humans"[MeSH Terms] AND English[lang] AND ("1980/01/01"[PDAT] : "2014/09/30"[PDAT])
Ischemic heart disease	physical activity[Title/Abstract] AND ischaemic heart disease [Title/Abstract] AND "humans"[MeSH Terms] AND English[lang] AND ("1980/01/01"[PDAT] : "2014/09/30"[PDAT])
Ischemic heart disease	physical activity[Title/Abstract] AND coronary heart disease [Title/Abstract] AND "humans"[MeSH Terms] AND English[lang] AND ("1980/01/01"[PDAT] : "2014/09/30"[PDAT])
Ischemic stroke	physical activity[Title/Abstract] AND ischemic stroke [Title/Abstract] AND "humans"[MeSH Terms] AND English[lang] AND ("1980/01/01"[PDAT] : "2014/09/30"[PDAT])
Ischemic stroke	physical activity[Title/Abstract] AND ischaemic stroke[Title/Abstract] AND "humans"[MeSH Terms] AND English[lang] AND ("1980/01/01"[PDAT] : "2014/09/30"[PDAT])

Data Extraction

Data will be extracted by two reviewers using a standardized data extraction form, and a third reviewer will independently check the data. The following variables will be extracted from each included study: author's last name, year of publication, study location (country), follow-up duration (years), sex, age at baseline, type of physical activity (leisure/recreational, domestic, occupational and/or transport related activity), measurement method of physical activity, category, duration, frequency and/or intensity of physical activity, dose of physical activity (e.g., minutes per week, MET hours per week), sample size, response rate, number of cases and participants in each category, and risk estimates and confidence intervals (age/sex adjusted and multivariate adjusted) for each activity category.

Risk of bias (quality) assessment

The quality of included studies will be assessed using the Newcastle-Ottawa scale (NOS) [2] in the following areas: (1) representativeness of the cohort (2) whether the non-exposed were drawn from the same population as the exposed; (3) ascertainment of exposure; (4) whether the outcome of interest was absent at the start of study; (5) comparability of the exposed and unexposed (i.e., adjustment for potential confounding variables); (6) ascertainment of the outcome; (7) whether the length of the follow-up was long enough (≥5 years) for the outcome to occur; and (8) the completeness of the follow-up (loss to follow up <20%). A maximum score of two can be awarded for comparability and a maximum score of one can be given for each of the remaining items. A study can have a maximum possible quality score of nine.

Strategy for data synthesis

We will use Dismod-MR 2.0, GBD's Bayesian meta-regression tool, to pool effect sizes from included studies and generate a dose-response total physical activity curve for each of the five outcomes. The tool will enable us to incorporate random effects across studies, and include data with different activity ranges and variation in categorization across studies. We will use a log-Gaussian likelihood function in a mixed-effects model as shown in the equation below:

$$-log\left[p\left(y_j|\Phi\right)\right] = \log\left(\sqrt{2\pi}\right) + \log\left(\delta_j + s_j\right) + \frac{1}{2}\left(\frac{\log\left(a_j + \eta_j\right) - \log\left(m_j + \eta_j\right)}{\delta_j + s_j}\right)^2$$

where, y_j is a data point; Φ denotes all model random variables; η_j is the offset value and a_j is the adjusted value for study level covariates for data point j, defined by:

$$a_j = e^{(-u_j - c_j)} y_j$$

where u_j is the study random effects and c_j is the total covariate effect (i.e. the mean combined fixed effects for sex and study level covariates), defined by:

$$c_j = \sum_{k=0}^{K[I(j)]-1} \beta_{I(j),k} \hat{X}_{k,j}$$

with standard deviation

$$s_j = \sum_{l=0}^{L[I(j)]-1} \zeta_{I(j),l} \hat{Z}_{k,j}$$

where k denotes the mean value of each data point in relation to a study-level covariate (also called x-covariate); $I(j)$ denotes a data point j; $\beta_{I(j),k}$ is the multiplier of the kth x-covariate; $\hat{X}_{k,j}$ is the covariate value corresponding to the data point j for covariate k; l denotes the standard deviation of each data point in relation to a covariate (also called z-covariate); $\zeta_{I(j),k}$ is the multiplier of the lth z-covariate; and δ_j is the standard deviation for adjusted measurement j where

m_j denotes the expected value for the jth measurement, not counting effects or measurement noise and defined by:

$$m_j = \frac{1}{B(j)-A(j)} \int_{A(j)}^{B(j)} I_j(\mathrm{a})\,\mathrm{da}$$

where *A(j)* is the lower bound of the MET-hour range for a data point; *B(j)* is the upper bound of the MET-hour range for a data point; and *Ij* denotes the function of MET-hour corresponding to the data point *j*. The basic settings of DisMod-MR 2.0 also estimates the parameter 'zeta' (ζ) that determines how much heterogeneity (non-sampling variation) between studies exists. An estimated value of zeta close to 0.5 indicates that there is a lot of heterogeneity in the data. We used non-increasing priors based on the literature to inform the analyses.

The dose-response meta-analysis will be run separately for each of the five outcomes. We will include study covariates indicating whether or not the MET-minutes are estimated using the GBD 2013 exposure data, whether a study reported relative risk, odds ratio or hazard ratio, and a sex covariate (male, female or both sexes) (sex will not be included as a covariate in the meta-analysis for breast cancer since we will focus only on breast cancer among females).

Publication bias will be assessed using funnel plots and the Egger test [3]. The influence of possible publication bias (if any) will be assessed by sensitivity analyses using the trim and fill method [4] which identifies potentially missing studies and corrects for funnel plot asymmetry. The impact of quality of studies on the findings will be assessed by sensitivity analyses including only higher quality studies. Study attributes such as qualitative versus quantitative categorization will also be evaluated for possible bias.

Source of funding

Bill & Melinda Gates Foundation.

Conflicts of interest

None.

Language

English

References

1. World Health Organization. Global Physical Activity Questionnaire (GPAQ) Analysis Guide [June 9, 2015]. Available from: http://www.who.int/chp/steps/resources/GPAQ_Analysis_Guide.pdf.
2. Wells G, Shea B, O'Connell D, et al. The Newcastle-Ottawa Scale (NOS) for assessing the quality of nonrandomized studies in meta-analyses. Department of Epidemiology and Community Medicine, University of Ottawa, Canada. www ohr i ca/programs/clinical_ epidemiology/oxford htm. 2011.
3. Egger M, Smith GD, Schneider M, et al. Bias in meta-analysis detected by a simple, graphical test. BMJ. 1997;315(7109):629-34.
4. Duval S, Tweedie R. A nonparametric "trim and fill" method of accounting for publication bias in meta-analysis. Journal of the American Statistical Association. 2000;95(449):89-98.
