# Supplementary material for: Physical activity and risk of breast cancer, colon cancer, diabetes, ischemic heart disease, and ischemic stroke events: systematic review and dose-response meta-analysis for the Global Burden of Disease Study 2013
Source: BMJ. 2016 Aug 9;354:i3857. doi: 10.1136/bmj.i3857 (PMC4979358; doi:10.1136/bmj.i3857)
Supplement: Supplementary file 2 — Appendix 2: Search strategies [file kyuh031211.ww2_default.pdf]

Appendix 2: Search strategies [posted as supplied by author]

EMBASE	Search strategies
Breast cancer	'physical activity':ab,ti AND 'breast cancer':ab,ti AND ([article]/lim OR [article in press]/lim) AND [humans]/lim AND [english]/lim AND [embase]/lim NOT [28-2-2016]/sd AND [1980-2016]/py
Breast cancer	'physical activity':ab,ti AND 'breast carcinoma':ab,ti AND ([article]/lim OR [article in press]/lim) AND [humans]/lim AND [english]/lim AND [embase]/lim NOT [28-2-2016]/sd AND [1980-2016]/py
Breast cancer	'physical activity':ab,ti AND 'breast neoplasm':ab,ti AND ([article]/lim OR [article in press]/lim) AND [humans]/lim AND [english]/lim AND [embase]/lim NOT [28-2-2016]/sd AND [1980-2016]/py
Breast cancer	'physical activity':ab,ti AND 'breast tumor':ab,ti AND ([article]/lim OR [article in press]/lim) AND [humans]/lim AND [english]/lim AND [embase]/lim NOT [28-2-2016]/sd AND [1980-2016]/py
Colon cancer	'physical activity':ab,ti AND 'colon cancer':ab,ti AND [humans]/lim AND [english]/lim AND [embase]/lim NOT [28-2-2016]/sd AND [1980-2016]/py
Colon cancer	'physical activity':ab,ti AND 'colon carcinoma':ab,ti AND [humans]/lim AND [english]/lim AND [embase]/lim NOT [28-2-2016]/sd AND [1980-2016]/py
Colon cancer	'physical activity':ab,ti AND 'colon neoplasm':ab,ti AND [humans]/lim AND [english]/lim AND [embase]/lim NOT [28-2-2016]/sd AND [1980-2016]/py
Colon cancer	'physical activity':ab,ti AND 'colon tumor':ab,ti AND [humans]/lim AND [english]/lim AND [embase]/lim NOT [28-2-2016]/sd AND [1980-2016]/py
Diabetes	'physical activity':ab,ti AND 'type 2 diabetes':ab,ti AND [1980-2016]/py AND ([article]/lim OR [article in press]/lim) AND [humans]/lim AND [english]/lim AND [embase]/lim NOT [28-2-2016]/sd
Diabetes	'physical activity':ab,ti AND 'noninsulin dependent diabetes mellitus':ab,ti AND [humans]/lim AND [english]/lim AND [embase]/lim NOT [28-2-2016]/sd AND [1980-2016]/py
Diabetes	'physical activity':ab,ti AND 'niddm':ab,ti AND [humans]/lim AND [english]/lim AND [embase]/lim NOT [28-2-2016]/sd AND [1980-2016]/py
Ischemic heart disease	'physical activity':ab,ti AND 'ischemic heart disease':ab,ti AND [humans]/lim AND [english]/lim AND [embase]/lim NOT [28-2-2016]/sd AND [1980-2016]/py
Ischemic heart disease	'physical activity':ab,ti AND 'ischaemic heart disease':ab,ti AND [humans]/lim AND [english]/lim AND [embase]/lim NOT [28-2-2016]/sd AND [1980-2016]/py
Ischemic heart disease	'physical activity':ab,ti AND 'coronary heart disease':ab,ti AND [humans]/lim AND [english]/lim AND [embase]/lim NOT [28-2-2016]/sd AND [1980-2016]/py
Ischemic stroke	'physical activity':ab,ti AND 'ischemic stroke':ab,ti AND [humans]/lim

	AND [english]/lim AND [embase]/lim NOT [28-2-2016]/sd AND [1980-2016]/py
Ischemic stroke	'physical activity':ab,ti AND 'ischaemic stroke':ab,ti AND [humans]/lim AND [english]/lim AND [embase]/lim NOT [28-2-2016]/sd AND [1980-2016]/py
**PubMed**	
Breast cancer	physical activity[Title/Abstract] AND breast cancer [Title/Abstract] AND "humans"[MeSH Terms] AND English[lang] AND ("1980/01/01"[PDAT] : "2016/02/27"[PDAT])
Breast cancer	physical activity[Title/Abstract] AND breast carcinoma [Title/Abstract] AND "humans"[MeSH Terms] AND English[lang] AND ("1980/01/01"[PDAT] : "2016/02/27"[PDAT])
Breast cancer	physical activity[Title/Abstract] AND breast neoplasm [Title/Abstract] AND "humans"[MeSH Terms] AND English[lang] AND ("1980/01/01"[PDAT] : "2016/02/27"[PDAT])
Breast cancer	physical activity[Title/Abstract] AND breast tumor [Title/Abstract] AND "humans"[MeSH Terms] AND English[lang] AND ("1980/01/01"[PDAT] : "2016/02/27"[PDAT])
Colon cancer	physical activity[Title/Abstract] AND colon cancer [Title/Abstract] AND "humans"[MeSH Terms] AND English[lang] AND ("1980/01/01"[PDAT] : "2016/02/27"[PDAT])
Colon cancer	physical activity[Title/Abstract] AND colon carcinoma [Title/Abstract] AND "humans"[MeSH Terms] AND English[lang] AND ("1980/01/01"[PDAT] : "2016/02/27"[PDAT])
Colon cancer	physical activity[Title/Abstract] AND colon neoplasm [Title/Abstract] AND "humans"[MeSH Terms] AND English[lang] AND ("1980/01/01"[PDAT] : "2016/02/27"[PDAT])
Colon cancer	physical activity[Title/Abstract] AND colon tumor [Title/Abstract] AND "humans"[MeSH Terms] AND English[lang] AND ("1980/01/01"[PDAT] : "2016/02/27"[PDAT])
Diabetes	physical activity[Title/Abstract] AND type 2 diabetes[Title/Abstract] AND "humans"[MeSH Terms] AND English[lang] AND ("1980/01/01"[PDAT] : "2016/02/27"[PDAT])
Diabetes	physical activity[Title/Abstract] AND noninsulin dependent diabetes mellitus [Title/Abstract] AND "humans"[MeSH Terms] AND English[lang] AND ("1980/01/01"[PDAT] : "2016/02/27"[PDAT])
Diabetes	physical activity[Title/Abstract] AND niddm[Title/Abstract] AND "humans"[MeSH Terms] AND English[lang] AND ("1980/01/01"[PDAT] : "2016/02/27"[PDAT])
Ischemic heart disease	physical activity[Title/Abstract] AND ischemic heart disease [Title/Abstract] AND "humans"[MeSH Terms] AND English[lang] AND ("1980/01/01"[PDAT] : "2016/02/27"[PDAT])
Ischemic heart disease	physical activity[Title/Abstract] AND ischaemic heart disease [Title/Abstract] AND "humans"[MeSH Terms] AND English[lang] AND ("1980/01/01"[PDAT] : "2016/02/27"[PDAT])
Ischemic heart	physical activity[Title/Abstract] AND coronary heart disease

disease	[Title/Abstract] AND "humans"[MeSH Terms] AND English[lang] AND ("1980/01/01"[PDAT] : "2016/02/27"[PDAT])
Ischemic stroke	physical activity[Title/Abstract] AND ischemic stroke [Title/Abstract] AND "humans"[MeSH Terms] AND English[lang] AND ("1980/01/01"[PDAT] : "2016/02/27"[PDAT])
Ischemic stroke	physical activity[Title/Abstract] AND ischaemic stroke[Title/Abstract] AND "humans"[MeSH Terms] AND English[lang] AND ("1980/01/01"[PDAT] : "2016/02/27"[PDAT])
