# Supplementary material for: Physical activity and risk of breast cancer, colon cancer, diabetes, ischemic heart disease, and ischemic stroke events: systematic review and dose-response meta-analysis for the Global Burden of Disease Study 2013
Source: BMJ. 2016 Aug 9;354:i3857. doi: 10.1136/bmj.i3857 (PMC4979358; doi:10.1136/bmj.i3857)
Supplement: Supplementary file 3 — Appendix 3: Equations [file kyuh031211.ww3_default.pdf]

Appendix 3: DisMod-MR 2.0 equations [posted as supplied by author]

We used a log-Gaussian likelihood function in a mixed-effects model as shown in the equation below:

$$-log\left[p\left(y_j|\Phi\right)\right] = \log\left(\sqrt{2\pi}\right) + \log\left(\delta_j + s_j\right) + \frac{1}{2}\left(\frac{\log\left(a_j + \eta_j\right) - \log\left(m_j + \eta_j\right)}{\delta_j + s_j}\right)^2$$

where, y_j is a data point; Φ denotes all model random variables; η_j is the offset value and a_j is the adjusted value for study-level covariates for data point j, defined by:

$$a_j = e^{(-u_j - c_j)} y_j$$

where u_j is the study random effects and c_j is the total covariate effect (i.e. the mean combined fixed effects for sex and study-level covariates), defined by:

$$c_j = \sum_{k=0}^{K[I(j)]-1} \beta_{I(j),k} \hat{X}_{k,j}$$

with standard deviation

$$s_j = \sum_{l=0}^{L[I(j)]-1} \zeta_{I(j),l} \hat{Z}_{k,j}$$

where k denotes the mean value of each data point in relation to a study-level covariate (also called x-covariate); $I(j)$ denotes a data point j; $\beta_{I(j),k}$ is the multiplier of the kth x-covariate; $\hat{X}_{k,j}$ is the covariate value corresponding to the data point j for covariate k; l denotes the standard deviation of each data point in relation to a covariate (also called z-covariate); $\zeta_{I(j),k}$ is the multiplier of the lth z-covariate; and δ_j is the standard deviation for adjusted measurement j where m_j denotes the expected value for the jth measurement, not counting effects or measurement noise and defined by:

$$m_j = \frac{1}{B(j) - A(j)} \int_{A(j)}^{B(j)} I_j(\mathrm{a})\, \mathrm{da}$$

where $A(j)$ is the lower bound of the MET-hour range for a data point; $B(j)$ is the upper bound of the MET-hour range for a data point; and Ij denotes the function of MET-hour corresponding to the data point j. The basic settings of DisMod-MR 2.0 also estimates the parameter 'zeta' (ζ) that determines how much heterogeneity (non-sampling variation) between studies exists. An estimated value of zeta close to 0.5 indicates that there is substantial heterogeneity in the data.
