# Supplementary material for: Physical activity and risk of breast cancer, colon cancer, diabetes, ischemic heart disease, and ischemic stroke events: systematic review and dose-response meta-analysis for the Global Burden of Disease Study 2013
Source: BMJ. 2016 Aug 9;354:i3857. doi: 10.1136/bmj.i3857 (PMC4979358; doi:10.1136/bmj.i3857)
Supplement: Supplementary file 4 — Appendix 4: Supplementary tables A-G [file kyuh031211.ww4_2kb.pdf]

Appendix 4: Supplementary tables A-G [posted as supplied by author]

Table A. Characteristics and quality assessment of studies included in the meta-regression analysis for the association between physical activity and breast cancer

Author (Year)	Study Name	Country	Time Period	Age Range	Sex	Sample Size	Activity Type	Effect Type	Effect	Covariates adjusted	NOS score
Bardia (2006)	Iowa Women's Health Study	United States	1986-2003	55-69	Female	36363	Recreation	RR	Low: 1.00; Medium: 1.04 (0.94, 1.14); High: 0.91 (0.82, 1.01)	Age, Education, Family History of Breast Cancer, Age at Menarche, Number of Live Births, Age at First Live Birth, Oral Contraceptive Use, Use of Hormone Therapy, Alcohol, Smoking, BMI at Baseline, BMI at Age 18	7
Borch (2014)	Norwegian Women and Cancer (NOWAC) study	Norway	1991-2004. Mean follow up 8.2 years	34-70	Female	80202	Total daily activity	RR	Very Low: 1.06 (0.86, 1.30); Low: 1.05 (0.93, 1.19); Moderate: 1.00 High: 1.04 (0.92, 1.17); Very High: 0.91 (0.73, 1.12)	Height, BMI, Smoking Status, Smoking Duration (Pack Years), Age at Menarche, Use of Oral Contraceptives, Age at First Birth, Parity, Use of Hormone Replacement Therapy, Self-Reported Disease, History of Breast Cancer in the Participant’s Mother.	7
Breslow (2001)	NHANES I cohort followed prospectively through the Epidemioloic follow-up study	United States	1982-1992 Median follow up 9.2 years	24-75	Female	6160	Recreation	RR	Consistently Low: 1.00; Moderate/Inconsistent: 0.92 (0.62, 1.38); Consistently High: 0.58 (0.31, 1.07)	Height, BMI at Age 25, Adult Weight Change, Sample Design Variables	7

Author (Year)	Study Name	Country	Time Period	Age Range	Sex	Sample Size	Activity Type	Effect Type	Effect	Covariates adjusted	NOS score
	(NHEFS)										
Cerhan (1998)	Iowa 65+ Rural Health Study	United States	1982-1993	65-102	Female	1806	Recreation+ Household	RR	Inactive: 1.00; Moderate: 0.50 (0.20, 1.10); High: 0.30 (0.06, 1.10)	Age, Education, BMI, Age at Menstruation, Age at Menopause, Use of Hormone replacement therapy, Systolic BP	7
Chang (2006)	Part of prostate, lung, colorectal, and ovarian cancer screening trial	United States	1993-2003 Median follow-up 4.9 years	55-74	Female	27541	Recreation	RR	0 hr/week: 1.00; <1 hr/week: 0.91 (0.70, 1.17); 1 hr/week: 0.98 (0.74, 1.28); 2 hr/week: 0.93 (0.72, 1.20); 3 hr/week: 1.06 (0.82, 1.36); ≥4 hr/week: 0.81 (0.63, 1.05)	Center, Race, Height, Family History of Breast Cancer, History of Benign Breast Disease, Age at Menarche, Age at First Birth, Parity, Age at Menopause, Menopausal Hormone Therapy, Education, Energy Intake, BMI	6
Colditz (2003)	Nurses' Health Study II	United States	1989-1999	25-42	Female	110468	Recreation + walking	RR	<3 MET hr/week: 1.0; 3-8.9 MET hr/week: 1.05 (0.82, 1.33); 9-17.9 MET hr/week: 0.95 (0.74, 1.21); 18-26.9 MET hr/week: 1.03 (0.79, 1.35); ≥27 MET hr/week: 1.04 (0.82, 1.33)	Age, Height, Alcohol, Age At Menarche, Age At First Birth, Oral Contraceptive Use, History of Benign Breast Disease, Mother or Sister with Breast Cancer, BMI	7
Dallal (2007)	California Teachers Study	United States	1995-2002 Mean follow-up 6.6 years	20-79	Female	110599	Recreation	RR	Strenuous Activity: 0-0.5 hr/week: 1.00; 0.51-2.0 hr/week: 0.93 (0.85, 1.02); 2.01-3.50 hr/week: 0.88 (0.78, 0.99);	Race, Family History of Breast Cancer, Age at First Full-Term Pregnancy & Number Full-Term Pregnancies, Hormone	6

Author (Year)	Study Name	Country	Time Period	Age Range	Sex	Sample Size	Activity Type	Effect Type	Effect	Covariates adjusted	NOS score
									3.51-5.0 hr/week: 1.02 (0.88, 1.18); >5.0 hr/week: 0.80 (0.69, 0.94)<br><br>Moderate activity: 0-0.5 hr/week: 1.00; 0.51-2.0 hr/week: 1.02 (0.92, 1.13); 2.01-3.50 hr/week: 1.02 (0.91, 1.15); 3.51-5.0 hr/week: 0.99 (0.86, 1.14); >5.0 hr/week: 0.94 (0.81, 1.08)	Replacement Therapy Use and Menopausal Status, BMI, Smoking, Alcohol, History of Breast Biopsy, Mammography Screening	
Dorgan (1994)	Framingham Heart Study	United States	1954-1984	35-68	Female	2307	Total daily activity	RR	Low (Physical Activity Index 25-28): 1.00; Q2 (Physical Activity Index 29-30): 1.20 (0.70, 2.10); Q3 (Physical Activity Index 31-32): 1.30 (0.70, 2.40); High (Physical Activity Index 33-54): 1.60 (0.90, 2.90)	Age, Stratifying on Age at Baseline Examination, Number of Pregnancies, Menopausal Status, Age at First Pregnancy, Education, Occupation, Alcohol	8
Eliassen (2010)	Nurses' Health Study	United States	1986-2006	40-65	Female	95396	Recreation + walking	RR	<3 MET hr/week: 1.00; 3-<9 MET hr/week: 0.94 (0.86, 1.02); 9-<18 MET hr/week: 0.96 (0.88, 1.05); 18-<27 MET hr/week: 0.97	Age, age at Menarche, BMI, Height, Parity and Age at First Birth, Alcohol, Postmenopausal Hormone Use, Age at Menopause, Missing Age at Menopause, Family History of	7

Author (Year)	Study Name	Country	Time Period	Age Range	Sex	Sample Size	Activity Type	Effect Type	Effect	Covariates adjusted	NOS score
									(0.87, 1.08); ≥27 MET hr/week: 0.91 (0.83, 1.01)	Breast Cancer, History Benign Breast Disease	
Frisch (1987)	Former college athletes	United States	1925-1981	22-78	Female	5398	Recreation	RR	Inactive: 1.86 (1.00, 3.47); Active: 1.00	Age, Number of Pregnancies, Family History of Cancer, Leanness, Age at Menarche, Smoking, Use of Oral Contraceptives, Use of Hormones for Menopause Symptoms	4
Hastert (2013)	Vitamins and Lifestyle (VITAL) study cohort	United States	2000-2008 Mean follow up 6.7 years	50-76	Female	30797	Recreation	HR	Did Not Meet Recommendation (<150 Minutes/Week): 1.0 Met; Recommendation (>=150 Minutes/Week): 0.97 (0.81, 1.16)	Age, Education, Race, Mammography, Family History of Breast Cancer, Age at Menarche, Age at First Birth, Age at Menopause, Years of Estrogen plus Progestin Hormone Therapy Use, Daily Energy Intake, Body Fatness, Energy Density, Plant Foods, Red Meat, Alcohol	7
Hildebrand (2013)	CPS-II Nutrition Cohort	United States	1992-2009	50-74	Female	73615	Recreation	RR	None: 0.91 (0.81, 1.02); > 0 - 7.0 MET hr/week: 1.00; > 7.0 - 17.5 MET hr/week: 0.97 (0.90, 1.04); > 17.5 - 31.5 MET hr/week: 0.99 (0.92, 1.08); > 31.5 - 42.0 MET	Age, Race, Education, BMI, Weight Change, Alcohol Use, Smoking Status, Menopausal Hormone Use, Number of Live Births, Age at First Live Birth, Age at Menopause, Family	7

Author (Year)	Study Name	Country	Time Period	Age Range	Sex	Sample Size	Activity Type	Effect Type	Effect	Covariates adjusted	NOS score
									hr/week: 0.94 (0.82, 1.08); > 42.0 MET hr/week: 0.75 (0.63, 0.89)	History of Breast Cancer, Breast Cysts, Hysterectomy, Oophorectomy, Mammogram within Last Year.	
Howard (2009)	US Radiologic Technologists cohort	United States	1994-2005 Mean length of follow up 8.9 years	20-99	Female	45631	Recreation + walking	HR	0.0-9.5 MET hr/week: 1.00; 11.5-23.0 MET hr/week: 1.02 (0.83, 1.26); 23.5-45.5 MET hr/week: 1.02 (0.83, 1.25); 46.0-96.5 MET hr/week: 0.87 (0.70, 1.08); ≥97.0 MET hr/week: 0.91 (0.74, 1.13)	Age, BMI, Age at Menarche, Parity, Age at First Birth, Age at Menopause, Family History of Breast Cancer, Personal History of Breast Disease, Oral Contraceptive Use, Menopausal Hormone Therapy, Race, Smoking, Alcohol	6
Lee (2001)	Women's Health Study (United States)	United States	1992-1998. Mean follow-up 48 months	45+	Female	39322	Recreation	RR	<840 kJ/week: 1.00; 840-2519 kJ/week: 1.04 (0.77, 1.40); 2520-6299 kJ/week: 0.86 (0.64, 1.17); ≥6300 kJ/week: 0.80 (0.58, 1.12)	Age, Treatment Group, BMI, Alcohol, Age at Menarche, Age at First Pregnancy Lasting ≥6 Months, Number of Pregnancies Lasting ≥6 Months, Menopausal Status, Ever Use of Oral Contraceptives, Use of Postmenopausal Hormones, Family History Breast Cancer	6
Leitzmann (2008)	Breast Cancer Detection Demonstra	United States	1987-1998	40-93	Female	32269	Recreation+ Household+ Occupational	RR	105-244 MET hr/week: 1.00; 245-297 MET hr/week: 0.93	Age at Baseline, Family History of Breast Cancer, History of Benign	7

Author (Year)	Study Name	Country	Time Period	Age Range	Sex	Sample Size	Activity Type	Effect Type	Effect	Covariates adjusted	NOS score
	tion Project Follow-up Study								(0.80, 1.09); 298-339 MET hr/week: 0.98 (0.84, 1.14); 340-394 MET hr/week: 1 (0.85, 1.16); 395-721 MET hr/week: 0.87 (0.74, 1.02)	Breast Disease, Breast Cancer Screening History, Height, Age at Menarche, Age at Menopause, Age at First Live Birth, History of Oral Contraceptive Use, Menopausal Hormone Therapy, Education, Smoking, Dietary Fat Intake, Alcohol, BMI	
Luoto (2000)	Finnish adult health behaviour survey	Finland	1978-1995	15-64	Female	30548	Recreation	RR	<Once/Week: 1.00; Once/Week: 0.80 (0.58, 1.10); 2-3 Times/Week: 0.92 (0.78, 1.22); Daily: 1.01 (0.72, 1.42)	Age, Length of Follow-Up, Education, BMI, Parity, Age At First Birth	6
Margolis (2005)	Women's Lifestyle and Health Study	Norway, Swedend en	1991-2003, Mean follow up 9.1 years	30-49	Female	99504	Recreation	RR	None: 1.00; Low: 1.35 (0.96, 1.90); Moderate: 1.26 (0.91, 1.74); High: 1.19 (0.85, 1.67); Vigorous: 1.24 (0.85, 1.82)	Age, Education, BMI, Height, Smoking, Alcohol, Age at Menarche, Parity, Age at First Birth, Number of Months Breast Feeding, Oral Contraceptive Use, Family History of Breast Cancer, Menopausal Status, Country of Origin	7
Maruti (2008)	Nurses' Health Study II	United States	1997-2003	33-51	Female	64777	Recreation + walking	RR	<21.0 MET hr/week: 1.00; 21.0-29.9 MET hr/week: 0.98	Age, Average Childhood Body Shape, Oral Contraceptive Use,	6

Author (Year)	Study Name	Country	Time Period	Age Range	Sex	Sample Size	Activity Type	Effect Type	Effect	Covariates adjusted	NOS score
									(0.77, 1.25); 30.0-38.9 MET hr/week: 0.93 (0.72, 1.20); 39.0-53.9 MET hr/week: 0.74 (0.56, 0.97); ≥54.0 MET hr/week: 0.77 (0.59, 1.01)	History of Benign Breast Disease, Mother or Sister with Breast Cancer, Parity and Age at First Birth, Alcohol Consumption, Height	
McTiernan (2003)	WHI Observational Study	United States	1993-2002 Mean follow up 4.7 years	50-79	Female	74171	Recreation + Walking	RR	None: 1.00; ≤5 MET hr/week: 0.90 (0.77, 1.07); 5.1-10 MET hr/week: 0.82 (0.68, 0.97); 10.1-20 MET hr/week: 0.89 (0.76, 1.00); 20.1-40 MET hr/week: 0.83 (0.70, 0.98); >40 MET hr/week: 0.78 (0.62, 1.00)	Age, BMI, Hormone Therapy, Race, Region, Income, Education, Ever Breastfed, Hysterectomy, First-Degree Relative with Breast Cancer, Smoking, Parity, Age at First Birth, Mammograms in 5 Years before Study, Alcohol, Age at Menarche, Age at Menopause	7
Mertens (2006)	Atherosclerosis Risk in Communities (ARIC) Study	United States	1987-1999	45-64	Female	7994	Recreation	RR	Q1: 1.00; Q2: 1.12 (0.75, 1.68); Q3: 1.18 (0.80, 1.73); Q4: 1.31 (0.87, 1.96)	Age, Race, Study Center, Age at First Live Birth, Age at Menopause, Family History of Breast Cancer in a First-Degree Relative	8
							Occupation		Q1 (1.00-1.50): 1.00; Q2 (1.75-2.0): 0.75 (0.50, 1.22); Q3 (2.25-2.75): 0.84 (0.59, 1.19); Q4		

Author (Year)	Study Name	Country	Time Period	Age Range	Sex	Sample Size	Activity Type	Effect Type	Effect	Covariates adjusted	NOS score
									(3.00-5.00): 0.87 (0.61, 1.24)		
Moradi (1999)	Swedish nationwide censuses in 1960 & 1970	Swedenden	1960-1989	40-85	Female	704904	Occupational	RR	Sedentary: 1.10 (1.00, 1.10); Light: 1.10 (1.00, 1.10); Medium: 1.00 (1.00, 1.10); Very High/High: 1.00	Age, Calendar Year of Follow Up, Place of Residence, Socioeconomic Status	6
Moradi (2002)	Swedish Twin Registry	Swedenden	1967-1997 Mean follow-up 15 years.	42-70	Female	9539	Recreation<br><br>Occupation	RR	Sedentary: 1.00 Moderate: 0.90 (0.70, 1.20); Regular Activity: 0.80 (0.60, 1.20)<br><br>Sedentary: 1.00 Active: 0.90 (0.70, 1.20); Strenuous: 1.00 (0.70, 1.50)	Age	5
Peters (2009)	NIH-AARP Diet and Health Study	United States	1995-2003. Mean follow up 7 years	50-71	Female	182862	Recreation+ Housework	RR	Inactive: 1.00; <1 Time/Week: 1.00 (0.92, 1.08); 1-2 Times/Week: 0.96 (0.89, 1.03); 3-4 Times/Week: 0.97 (0.90, 1.04); ≥ 5 Times/Week: 0.92 (0.85, 1.00)	Age, Race/Ethnicity, Education, Smoking, Family History Breast Cancer, Menopausal Hormone Use, Age at First Birth, Age at Menarche, Age at Menopause, Parity, Alcohol, BMI	7
Pronk (2011)	Shanghai Women's Health Study (SWHS)	China	1996-2007	40-70	Female	74049	Recreation+ Household+ Commuting	HR	0-<74.3 MET hr/week: 1.00; 74.3-<100.2 MET hr/week: 1.07 (0.87, 1.30); 100.2-<131.5 MET hr/week: 0.96 (0.78, 1.18); 131.5+ MET hr/week: 0.98	Age, Education, Family History Breast Cancer, Age at First Birth, Number of Pregnancies	8

**Author (Year)**	**Study Name**	**Country**	**Time Period**	**Age Range**	**Sex**	**Sample Size**	**Activity Type**	**Effect Type**	**Effect**	**Covariates adjusted**	**NOS score**
							Occupational		(0.79, 1.21)<br><br><4.64: 1.00;<br>4.64-<9.61: 1.00 (0.82, 1.22); 9.61-<10: 0.95 (0.76, 1.18);<br>10: 0.73 (0.53, 0.99)		
Rintala (2002)	1970 population census of Finland (link job codes to the Finnish Cancer Registry)	Finland	1971-1995	25-89	Female	680000	Occupational	RR	Age 25-39:<br>Low (Class 1+2): 1.00;<br>Q2 (Class 3): 0.99 (0.85, 1.17);<br>Q3 (Class 4): 0.90 (0.76, 1.07);<br>High (Class 5): 0.68 (0.51, 0.93)<br><br>Age 40-54:<br>Low (Class 1+2): 1.00;<br>Q2 (Class 3): 1.02 (0.94, 1.11);<br>Q3 (Class 4): 0.99 (0.91, 1.09);<br>High (Class 5): 0.84 (0.70, 1.00)<br><br>Age 55-89:<br>Low (Class 1+2): 1.00;<br>Q2 (Class 3): 1.01 (0.96, 1.07);<br>Q3 (Class 4): 1.04 (0.98, 1.11);<br>High (Class 5): 0.82 (0.71, 0.94)	Social Class, Reproductive Factors	5
Rockhill (1999)	Nurses' Health	United States	1980-1996	34-59	Female	85364	Recreation	RR	<1 hr/week: 1.00;<br>1.0-1.9 hr/week:	Age at Baseline, Age at Menarche, History	7

Author (Year)	Study Name	Country	Time Period	Age Range	Sex	Sample Size	Activity Type	Effect Type	Effect	Covariates adjusted	NOS score
	Study								0.88 (0.79, 0.98); 2.0-3.9 hr/week: 0.89 (0.81, 0.99); 4.0-6.9 hr/week: 0.85 (0.77, 0.94); ≥7 hr/week: 0.82 (0.70, 0.97)	of Benign Breast Disease, History of Breast Cancer in Mother and/or Sister, Height, Parity and Age at First Birth, BMI at Age 18, Menopausal Status, Postmenopausal Hormone Use	
Rosenberg (2014)	Black Women's Health Study	United States	1995-2011	30-69	Female	44078	Recreation	RR	Vigorous Exercise, <1 H/Wk: 1.00; Vigorous Exercise, 1 H/Wk: 0.85 (0.70, 1.03); Vigorous Exercise, 2 H/Wk: 0.99 (0.83, 1.19); Vigorous Exercise, 3-4 H/Wk: 0.94 (0.79, 1.12); Vigorous Exercise, 5-6 H/Wk: 0.89 (0.69, 1.14); Vigorous Exercise, >= 7 H/Wk: 0.74 (0.57, 0.96)	Adjusted for Age (Single Year), Time Period (Questionnaire Cycle), Years of Education (12, 13–15, 16, 17), Parity (0, 1, 2, 3), Vegetable/Fruit Dietary Pattern (Quintiles), and Meat/Fried Foods Dietary Pattern (Quintiles)	5
Sesso (1998)	The College Alumni Health Study	United States	1962-1993	37-69	Female	1566	Recreation, Commuting	RR	<500 kcal/week: 1.00; 500-999 kcal/week: 0.92 (0.58, 1.45); 1000+ kcal/week: 0.73 (0.46, 1.14)	Age, BMI	3
Silvera (2006)	The Canadian National Breast Screening Study	Canada	1980-2000 mean follow-up 16.4	40-59	Female	40318	Recreation+ housework	HR	0-30 Min/Day: 1.06 (0.88, 1.27); 30-60 Min/Day: 0.98 (0.83, 1.16); >60 Min/Day: 0.93 (0.78, 1.10)	Age, Alcohol, Smoking, Oral Contraceptive Use, Hormone Replacement Therapy, Parity, Age	7

Author (Year)	Study Name	Country	Time Period	Age Range	Sex	Sample Size	Activity Type	Effect Type	Effect	Covariates adjusted	NOS score
	(NBSS)		years							at Menarche, Age at First Birth, Family History of Breast Cancer, History of Breast Disease, Menopausal Status at Baseline, Center, Randomization Group, Energy Intake, BMI	
Steindorf (2012)	European Prospective Investigation into Cancer and Nutrition (EPIC)	Denmark, France, Germany, Greece, Italy, Netherlands, Spain, United Kingdom, Sweden	1992-2008 Median length of follow up 11.6 years	20-98.5	Female	257805	Recreation+ Housework+ Commuting	HR	≤50.5 MET hr/week: 1.00; >50.5-81.4 MET hr/week: 0.94 (0.88, 0.99); >81.4-123 MET hr/week: 0.90 (0.84, 0.96); >123 MET hr/week: 0.87 (0.81, 0.94)	Age, Center, BMI, Age at First Period, Age at First Full Term Pregnancy, Number of Full Term Pregnancies, Breast Feeding, Oral Contraceptive Pill, Menopausal Status, Age at Menopause, Hormone Replacement Therapy, Alcohol, Smoking, Education	7
Suzuki (2008)	Japan Collaborative Cohort Study (JACC)	Japan	1988-2001 Median follow up 12.4 years	40-69	Female	30157	Recreation	HR	Never or Seldom: 1.0; 1-2 Hr/Day: 0.83 (0.56, 1.23); ≥3 Hr/Day: 0.85 (0.51, 1.40)	Age, BMI, Alcohol, Age at Menarche, Education, Parity, Age at First Birth, Use of Exogenous Female Hormone, Family History Breast Cancer First-Degree Relative, Menopausal Status, Menopausal Age	8
Suzuki (2011)	Japan Public Health Center-based	Japan	1990-2007	40-69	Female	23977	Total daily activity	RR	Tertile 1 (Low): 1.00; Tertile 2: 1.13 (0.82, 1.56); Tertile 3 (High):	Age, Area, Height, Recent BMI, BMI At Age 20, Smoking Status, Age at Menarche, Age at	7

Author (Year)	Study Name	Country	Time Period	Age Range	Sex	Sample Size	Activity Type	Effect Type	Effect	Covariates adjusted	NOS score
	Prospective Study								1.03 (0.75, 1.41)	First Birth, Parity, Age at Menopause, Use of Exogenous Hormones, Alcohol, Energy Adjusted Intake of Isoflavones, Participation Frequency in Leisure-Time Physical Activity	
Thune (1997)	National Health Screening Service in Norway	Norway	1974-1994 Median follow-up 13.7 years	20-54	Female	25624	Recreation<br><br>Occupation	RR	Sedentary: 1.00; Moderate: 0.93 (0.71, 1.22); Regular Exercise: 0.63 (0.42, 0.95)<br><br>Sedentary: 1.00; Walking: 0.84 (0.63, 1.12); Lifting: 0.74 (0.52, 1.06); Heavy Manual Labor: 0.48 (0.25, 0.92)	Age, BMI, Height, County of Residence, Number of Children	7
Wyrwich (2000)	Longitudinal Study on Aging (LSOA)	United States	1984-1991	70-98	Female	3131	Recreation+ Housework	HR	Inactive: 1.00; Moderate: 0.63 (0.38, 1.18); High: 0.51 (0.22, 1.16)	Prior Cancer, Age, BMI, Education, Excluding Breast Cancer Cases in First 2 Years	5
Wyshak (2000)	Alumnae listed as alive by the alumnae office of eight colleges and two	United States	1981-1996	22-78	Female	3940	Recreation	OR	Inactive: 1.00; Active: 0.61 (0.44, 0.84)	Age, Ever-Pregnant, Use of Contraceptives, Use of Hormone Replacement Therapy, Family History of Breast Cancer, Current Exercise, Ever	5

Author (Year)	Study Name	Country	Time Period	Age Range	Sex	Sample Size	Activity Type	Effect Type	Effect	Covariates adjusted	NOS score
	universities (former athletes vs former non-athletes)									Smoked, Percent Body Fat	

Table B. Characteristics and quality assessment of studies included in the meta-regression analysis for the association between physical activity and colon cancer

Author (Year)	Study Name	Country	Time Period	Age Range	Sex	Sample Size	Activity Type	Effect Type	Effect	Covariates adjusted	NOS score
Bostick (1994)	The Iowa Women's Health Study cohort	United States	1986-1990	55-69	Female	35215	Recreation	RR	Low: 1.00; Moderate: 0.92 (0.68, 1.28); Vigorous: 0.95 (0.68, 1.39)	Age, Total Energy Intake, Height, Parity, Total Vit E Intake, Total Vit E by Age Interaction Term, Vit A Supplement Intake	5
Calton (2006)	The Breast Cancer Detection Demonstration Project (BCDDP)	United States	1987-1998	55-75	Female	31783	All activity	RR	34-48.5 MET hr/day: 1.0; 48.51-54.3 MET hr/day: 1.45 (0.98, 2.15); 54.31-59.0 MET hr/day: 1.16 (0.77, 1.75); 59.1-64.9 MET hr/day: 1.27 (0.84, 1.91); 65.0-91.1 MET	Age, BMI, Education, History of Colorectal Cancer, Smoking, Menopausal Hormone Use, Aspirin Use, Alcohol, Energy-Adjusted	8

Author (Year)	Study Name	Country	Time Period	Age Range	Sex	Sample Size	Activity Type	Effect Type	Effect	Covariates adjusted	NOS score
									hr/day: 1.15 (0.76, 1.75)	Intake Calcium, Energy-Adjusted Intake Red Meat	
Chao (2004)	The Cancer Prevention Study II Nutrition Cohort	United States	1992-1999	50-74	Both	151174	Recreation	RR	No Activity: 1.00; <7 MET hr/week: 0.93 (0.75, 1.16); 7-13 MET hr/week: 0.88 (0.68, 1.13); 14-23 MET hr/week: 0.84 (0.66, 1.06); 24-29 MET hr/week: 0.89 (0.68, 1.15); ≥30 MET hr/week: 0.65 (0.49, 0.87)	Age, Education, Exercise Level in 1982, Cigarette Smoking, Alcohol, Red Meat, Folate, Fiber, Multivitamin Use in 1982, Hormone Replacement Therapy in women, Sex	6
Colbert (2001)	The Alpha-Tocopherol, Beta-Carotene Cancer Prevention study	Finland	1985-1997 Mean follow-up 6 years.	50-69	Male	29133	Recreation<br><br>Occupational	RR	Sedentary: 1.00; Active: 0.82 (0.59, 1.13)<br><br>Sedentary: 1.00; Light: 0.60 (0.34, 1.04); Moderate/Heavy: 0.45 (0.26, 0.78)	Age, Supplement Group, BMI, Cigarettes/Day	6
Fraser (1993)	New Zealand Cancer Registry	New Zealand	1972-1980	15-64	Male	1651	Occupational	RR	Sedentary: 1.18 (1.02, 1.36); High: 1.00	-	3
Friedenreich (2006)	The EPIC study	France, Italy, Spain, United	1992-2003	35-70	Both	413044	Recreation	HR	<12.0 MET hr/week: 1.00; ≥12.0-<24.8 MET hr/week: 0.85 (0.71,	Age, Study Center, Kcal/Day Quartiles,	7

Author (Year)	Study Name	Country	Time Period	Age Range	Sex	Sample Size	Activity Type	Effect Type	Effect	Covariates adjusted	NOS score
		Kingdom, Netherlands, Greece, Germany, Swedenden, Denmark							1.00); ≥24.8-<42.8 MET hr/week: 0.83 (0.70, 0.98); ≥42.8 MET hr/week: 0.88 (0.74, 1.05)	Education, Smoking, Height, Weight, Fiber	
Gerhardsson (1986)	Swedish census	Sweden	1960-1979	20-64	Male	1100000	Occupational	RR	Sedentary: 1.30 (1.20, 1.50); Active: 1.00	Age, Population Density, Social Class	5
Giovannucci (1995)	Health Professionals Follow-up Study	United States	1986-1992	40-75	Male	47723	Recreation	RR	By MET hr/week quintile (Quintile median MET hr/week): Q1 (0.9 MET hr/week): 1.00; Q2 (4.8 MET hr/week): 0.73 (0.48, 1.10); Q3 (11.3 MET hr/week): 0.94 (0.63, 1.39); Q4 (22.6 MET hr/week): 0.78 (0.51, 1.20); Q5 (46.8 MET hr/week): 0.53 (0.32, 0.88)	BMI, Age, History Endoscopic Screening or Polyp Diagnosis, Parental History of Colorectal Cancer, Pack-Years Smoking, Aspirin Use, Folate Intake, Methione Intake, Alcohol, Fiber Intake, Red Meat Intake, Total Energy Intake	7
Howard (2008)	The NIH-AARP Diet and Health	United States	1995-2003. Mean follow up	50-71	Male<br>Female	175600 (males)<br>125073	Recreation+ Household	RR	By MET hr/week quintile (Quintile Median) Male:	Age, Smoking, Alcohol, Education, Race, Family	7

Author (Year)	Study Name	Country	Time Period	Age Range	Sex	Sample Size	Activity Type	Effect Type	Effect	Covariates adjusted	NOS score
	Study		6.9 years.			(females)			Q1 (5.53 MET hr/week): 1.00; Q2 (16.52 MET hr/week): 0.87 (0.74, 1.04); Q3 (29.97 MET hr/week): 0.77 (0.65, 0.91); Q4 (45.43 MET hr/week): 0.68 (0.57, 0.81); Q5 (66.08 MET hr/week): 0.79 (0.66, 0.94)<br><br>Female:<br>Q1 (8.07 MET hr/week): 1.00; Q2 (20.99 MET hr/wek): 0.94 (0.74, 1.18); Q3 (35.72 MET hr/week): 0.85 (0.64, 1.13); Q4 (53.43 MET hr/week): 0.92 (0.73, 1.17); Q5 (66.08 MET hr/week): 0.92 (0.71, 1.18)	History Colon Cancer, Total Energy, Energy-Adjusted Intake Red Meat, Calcium, Whole Grains, Fruit, Vegetables, BMI, hormone therapy (for female analysis)	
Larsson (2006)	The cohort of Swedish men	Sweden	1997-2005. Mean follow up 7.1 years	45-79	Male	45906	All activity	HR	<37.9 MET hr/day: 1.00; 37.9-40.7 MET hr/day: 0.84 (0.62, 1.13); 40.8-44.8 MET hr/day: 1.00 (0.76, 1.33); ≥44.9 MET hr/day: 0.82 (0.60, 1.10)	Age, Education, Family History of Colorectal Cancer, History of Diabetes, Smoking, Aspirin Use, BMI	7

Author (Year)	Study Name	Country	Time Period	Age Range	Sex	Sample Size	Activity Type	Effect Type	Effect	Covariates adjusted	NOS score
Lee (1994)	Harvard Alumni Study	United States	1962-1988	30-79	Male	17607	Recreation+transport	RR	<1000 kcal/day: 1.00;<br>1000-2499 kcal/day: 0.75 (0.42, 1.35);<br>≥2500 kcal/day: 0.94 (0.54, 1.64)	Age, Quetelet's Index, Parental History of Cancer	5
Lee (1997)	The Physicians ' Health Study	United States	1982-1994. Mean follow up 10.9 years	40-84	Male	21807	Recreation	RR	Frequency of vigorous exercise:<br><1 time per/week: 1.00;<br>1 time/week: 1.10 (0.70, 1.70);<br>2-4 times/week: 1.20 (0.80, 1.60);<br>5+ times/week: 1.10 (0.70, 1.60)	Age, Obesity, Alcohol, Randomization Assignment	6
Lee (2007)	Japan Public Health Center-based Prospective Study	Japan	1995-2002. Mean follow up 5.8 years.	45-74	Male<br><br>Female	29842 (males)<br><br>35180 (females)	All activity	RR	By MET hr/day quartile (quintile medina in MET hr/day)<br>Males:<br>Q1 (28.25 MET hr/day): 1.00;<br>Q2 (33.25 MET hr/day): 0.87 (0.61, 1.26);<br>Q3 (35.25 MET hr/day): 0.62 (0.41, 0.95);<br>Q4 (43.75 MET hr/day): 0.58 (0.39, 0.87)<br><br>Females:<br>Q1 (28.5 MET hr/day): 1.00;<br>Q2 (33.25 MET hr/day): 1.03 (0.65, 1.64);<br>Q3 (35.25 MET hr/day): 0.91 (0.57,	Age, Study Area, Family History of Colorectal Cancer, Smoking, Alcohol, BMI, Intake Red Meat, Intake Dietary Fiber, Intake Folate	8

Author (Year)	Study Name	Country	Time Period	Age Range	Sex	Sample Size	Activity Type	Effect Type	Effect	Covariates adjusted	NOS score
									1.47);<br>Q4 (43.75 MET hr/day): 0.89 (0.54, 1.49)		
Mai (2007)	California Teachers Study	United States	1995-2002 Mean length of follow up 6.6 years	22-84	Female	120147	Recreation	RR	Lifetime strenuous activity:<br>0-0.5 hr/week: 1.00;<br>0.51-1.99 hr/week: 0.98 (0.77, 1.24);<br>≥2.0 hr/week: 0.99 (0.78, 1.27)<br><br>Lifetime moderate activity"<br>0-0.5 hr/week: 1.00;<br>0.51-1.99 hr/week: 0.74 (0.57, 0.95);<br>≥2.0 hr/week: 0.79 (0.62, 1.00)	Race, Age	5
Moradi (2008)	Swedish census of the Population and Housing	Sweden	1960-1989	25-85	Male<br><br>Female	1343696 (males)<br><br>699519 (females)	Occupational	RR	Male:<br>Sedentary: 1.30 (1.20, 1.40);<br>Light: 1.30 (1.20, 1.30);<br>Moderate: 1.20 (1.10, 1.20);<br>Very High/High: 1.00<br><br>Female:<br>Sedentary: 1.10 (1.10, 1.30);<br>Light: 1.10 (1.00, 1.20);<br>Moderate: 1.00 (0.90, 1.10);<br>Very High/High: 1.00	Age, Calendar Year of Follow-Up, Place of Residence, Socioeconomic Status	5
Nilsen (2008)	Nord-Trondelag	Norway	1984-2002	20-101	Both	59369	Recreation	HR	No Activity: 1.00;<br>Low: 0.87 (0.70,	Sex, BMI, Smoking	7

Author (Year)	Study Name	Country	Time Period	Age Range	Sex	Sample Size	Activity Type	Effect Type	Effect	Covariates adjusted	NOS score
	Health Study								1.08);<br>High: 0.73 (0.58, 0.92)	Status, Alcohol, Education, Marital Status (used attained age as the time variable)	
Severson (1989)	Cohort of Japanese men living on the Hawaiian island of Oahu in 1965	United States	1965-1986	46-65	Male	8006	All 24-hour activity	RR	Lowest tertile: 1.00;<br>Middle tertile: 0.56 (0.39, 0.80);<br>Highest tertile: 0.71 (0.51, 0.99)	Age, BMI	5
Thune (1996)	A Populatio-based cohort in Norway	Norway	1972-1991. Mean follow up 16.3 years in males and 15.5 years in females	20-49	Male<br><br>Female	52242 (males)<br><br>28274 (females)	Work+Recreation	RR	Males:<br>Sedentary: 1.00;<br>Moderate: 1.18 (0.76, 1.82);<br>Active: 0.97 (0.63, 1.5)<br><br>Females:<br>Sedentary: 1.00<br>Moderate: 0.97 (0.33, 2.77)<br>Active: 0.63 (0.39, 1.04)	Age at Entry, BMI, Geographic Region	6
Wolin (2007)	Nurses' Health Study	United States	1986-2002	40-65	Female	79295	Recreation	RR	<2 MET hr/week: 1.00;<br>2.1-4.5 MET hr/week: 0.88 (0.68, 1.14);<br>4.6-10.3 MET hr/week: 0.91 (0.70, 1.17);<br>10.4-21.4 MET hr/week: 0.82 (0.62, 1.07);<br>≥21.5 MET	Age, BMI, Smoking, Multivitamin Use, Aspirin, Alcohol, Red Meat Intake, Vit D, Calcium, Family History Colon Cancer, Endoscopy, History of	7

Author (Year)	Study Name	Countr y	Time Period	Age Range	Sex	Sample Size	Activity Type	Effect Type	Effect	Covariates adjusted	NOS score
									hr/week: 0.77 (0.58, 1.01)	Polyps	

Table C. Characteristics and quality assessment of studies included in the meta-regression analysis for the association between physical activity and diabetes

**Author (Year)**	**Study Name**	**Country**	**Time Period**	**Age Range**	**Sex**	**Sample Size**	**Activity Type**	**Effect Type**	**Effect**	**Covariates adjusted**	**NOS score**
Baan (1999)	The Rotterdam Study	Netherlands	1990-1994	55-75	Male<br><br>Female	503 (males)<br><br>513 (females)	Recreation	OR	Males:<br><847 min/week: 1.00;<br>847-<1860 min/week: 0.67 (0.32, 1.43);<br>≥1860 min/week: 0.74 (0.34, 1.59)<br><br>Females:<br><1245 min/week: 1.00;<br>1245-<2007 min/week: 0.89 (0.40, 1.99);<br>≥2007 min/week: 0.80 (0.34, 1.88)	Age, BMI, Waist-Hip Ratio, Family History of Diabetes, Smoking	5
Bonora (2004)	The Bruneck Study	Italy	1990-2000	40-80	Both	7884	Recreation and Occupation	OR	Low Physical Activity: 1.00; High Physical Activity: 0.80 (0.50, 1.40)	Age and Sex	6
Burchfiel (1995)	Honolulu Heart Program	United States	1965-1974	45-68	Male	6811	Total	OR	Lower Four Quintiles: 1.00; Upper Quintile: 0.50 (0.34, 0.73)	Age, Body Mass Index, Subscapularlt riceps Skinfold Ratio, Systolic Blood Pressure, Triglycerides, Glucose, Hematocrit, and Parental History of Diabetes. Free of	6

Author (Year)	Study Name	Country	Time Period	Age Range	Sex	Sample Size	Activity Type	Effect Type	Effect	Covariates adjusted	NOS score
										Prevalent and Incident Cardiovascular Disease during Study Period.	
Carlsson (2007)	Nord-Trøndelag Health Survey	Norway	1984-1997 Mean follow up 6.5 years.	≥20 years	Both	38128	Recreation	RR	Never: 2.03 (1.52, 2.71); Less Than Once Per Week: 1.61 (1.24, 2.10); Once Per Week: 1.24 (0.94, 1.62); Two or Three Times Per Week: 1.21 (0.92, 1.59); Every Day: 1.00	Sex, BMI, Smoking	5
Carlsson (2013)	The Swedish Twin Registry	Sweden	1967-2002	30-99	Male	23539	Recreation	HR	Low: 1.00 Moderate: 0.77 (0.61, 0.96); High: 0.53 (0.37, 0.75)	Smoking, Occupation, Alcohol Consumption	5
Chien (2009)	Chin-Shan community cardiovascular cohort study	China	1990-2000 Mean follow up 5 years.	≥35	Both	11690<br><br>12251<br><br>12566	Occupation<br><br>Recreation<br><br>Sports	RR	Q1: 1.00; Q2: 0.75 (0.54, 1.05); Q3: 0.75 (0.50, 1.12); Q4: 0.97 (0.69, 1.37)<br><br>Q1: 1.00; Q2: 1.09 (0.80, 1.49); Q3: 1.07 (0.77, 1.48); Q4: 1.24 (0.87, 1.75)	Metabolic Syndrome, BMI, Gender, and Age Groups, Smoking, Current Alcohol Drinking (Regular/No), Marital Status (Single, Married and Living with Spouse, or Divorced and	8

Author (Year)	Study Name	Country	Time Period	Age Range	Sex	Sample Size	Activity Type	Effect Type	Effect	Covariates adjusted	NOS score
									Q1: 1.00; Q2: 0.82 (0.60, 1.12); Q3: 0.65 (0.47, 0.89); Q4: 0.68 (0.49, 0.95)	Separated), Education Level (Less Than 9 Years, at Least 9 Years), Occupation (No Work, Labor, Official or Business), Hypertension Status, HDL-C, Triglycerides, Glucose Levels and Family History of Diabetes.	
Demakakos (2010)	ELSA	United Kingdom	2002-2007. Mean follow up 45.3 months.	50+	Both	7466	Recreation	HR	None: 1.00; Low Intensity: 0.87 (0.58, 1.30); Vigorous/Moderate Intensity: 0.64 (0.43, 0.95)	Age, Age-Squared, Sex, Marital Status, Educational Attainment, Total Household Wealth, BMI, Self-Reported Long-Standing Illness/Disability, Cardiovascular Comorbidities, Non-Cardiovascular Comorbiditie	4

Author (Year)	Study Name	Country	Time Period	Age Range	Sex	Sample Size	Activity Type	Effect Type	Effect	Covariates adjusted	NOS score
										s, Health Behaviours, Elevated Depressive Symptoms	
Doi (2012)	Population-based prospective study of cardiovascular disease and its risk factors	Japan	1988-2002 Mean follow up 11.8 years.	20-99	Both	1935	Recreation	HR	Regular exercise: 0.69 (0.43, 1.1)	Age, Sex, Family History of Diabetes, Central Obesity, BMI, Hypertension, Smoking (/Day), FPG Levels	8
Dotevall (2004)	The BEDA Study	Sweden	1980-1998	39-65	Female	1351	Recreation and Occupation	HR	Not Sedentary: 1.00 Sedentary: 1.56 (0.96, 2.53)	Age, BMI, Triglycerides, and SBP	6
Elwood (2013)	Caerphilly Cohort Study	United Kingdom	1979-2009	45-59	Male	2235	Transportation and Recreation	OR	No Regular Exercise: 1.00; Regular Exercise: 0.63 (0.46, 0.85)	Age, Social Class	5
Fan (2015)	China Multicenter Collaborative Study of Cardiovascular Epidemiology & China Cardiovascular Health Study	China	1998-2008 Mean follow up 7.9 years.	35-74	Both	6348	Total	HR	Sedentary (Pal, 1.00–1.39): 1.00; Low Active (Pal, 1.40–1.59): 0.92 (0.69, 1.22); Active (Pal, 1.60–1.89): 0.67 (0.50, 0.89); Very Active (Pal >1.89): 0.59 (0.45, 0.77)	Age, Sex, Geographic region (North or South), Educational level (0–6, 7–9, or ≥10 yrs), cigarette smoking (never, ever, or current), alcohol consumption (yes or no), and family history of	6

Author (Year)	Study Name	Country	Time Period	Age Range	Sex	Sample Size	Activity Type	Effect Type	Effect	Covariates adjusted	NOS score
										diabetes (yes or no), waist circumstance	
Folsom (2000)	Iowa Women's Health Study Cohort	United States	1986-1997	55-69	Female	34257	Recreation	RR	Low: 1.00; Medium: 0.91 (0.82, 1.02); High: 0.79 (0.70, 0.90)	Age, Education, Smoking, Alcohol, Estrogen Replacement, Energy Intake, Whole Grain Intake, Keys' Score, Family History Diabetes, BMI, Waist-To-Hip Ratio	6
Fretts (2009)	The Strong Heart Study	United States	1989-1999 Mean follow up 5 years.	45-74	Both	1651	Recreation and Occupation	OR	No activity: 1.00; <30 MET-h/wk: 0.70 (0.46, 1.03); 30-106 MET-h/wk: 0.72 (0.47, 1.08); >106 MET-h/wk: 0.71 (0.47, 1.07)	Age, Study Site, Sex, Education, Cigarette Smoking, Alcohol Use, Family History of Diabetes, Systolic Blood Pressure, Diastolic Blood Pressure, High Density Lipoprotein Cholesterol, Low Density Lipoprotein Cholesterol, Plasma Fibrinogen,	7

Author (Year)	Study Name	Country	Time Period	Age Range	Sex	Sample Size	Activity Type	Effect Type	Effect	Covariates adjusted	NOS score
										Body Mass Index	
Grontved (2012)	Health Professionals Follow-up Study	United States	1990-2008	40-75	Male	32002	Recreation	RR	0 Mins/Wk: 1.00; 1-59 Mins/Wk: 0.92 (0.81, 1.05); 60-149 Mins/Wk: 0.67 (0.58, 0.78); >=150 Mins/Wk: 0.46 (0.40, 0.52)	Age, Smoking, Alcohol Consumption, Coffee Intake, Race, Family History of Diabetes, Intake of Total Energy, Trans Fat, Polyunsaturated Fat To Saturated Fat Ratio, Cereal Fiber, Whole Grain, and Glycemic Load.	6
Grontved (2014)	Nurses' Health Study<br><br>Nurses' Health Study II	United States	2000-2008<br><br>2001-2009	53-81	Female	51642<br><br>47674	Recreation and Transportation	RR	None: 1.00; 1-29 Mins/Wk: 0.94 (0.81, 1.09); 30-59 Mins/Wk: 0.88 (0.76, 1.02); 60-150 Mins/Wk: 0.85 (0.74, 0.96); >150 Mins/Wk: 0.66 (0.58, 0.75)<br><br>None: 1.00; 1-29 Mins/Wk: 0.94 (0.79, 1.13); 30-59 Mins/Wk: 0.83 (0.69, 1.00); 60-150 Mins/Wk: 0.86 (0.73, 1.01);	Age, Smoking, Alcohol Consumption, Coffee Intake, Race, Family History of Diabetes, Post Menopausal Hormone Use, Intake of Total Energy, Trans Fat, Polyunsaturated Fat to Saturated Fat Ratio, Cereal	6

Author (Year)	Study Name	Country	Time Period	Age Range	Sex	Sample Size	Activity Type	Effect Type	Effect	Covariates adjusted	NOS score
									>150 Mins/Wk: 0.70 (0.59, 0.83)	Fiber, Wholegrain, and Glycemic Load, Oral Contraceptive Use, Menopausal Status, Resistance Exercise, Lower Intensity Muscular Conditioning Exercises, and BMI	
Gurwitz (1994)	The East Boston Senior Health Project	United States	1982-1989	65+	Both	2737	Recreation+ Housework	OR	Low: 1.50 (1.00, 2.10);<br>Moderate: 1.00<br>High: 1.40 (0.84, 2.40)	Age, Sex, BMI, Alcohol, Blood Pressure, Self-Reported High Blood Sugar	6
Haapanen (1997)	Census data of a medium-size industrial town and two rural municipalities in northeastern Finland	Finland	1980-1990	35-63	Male<br><br>Female	891 (males)<br><br>973 (female)	Recreation, Household, Commuting	RR	Males:<br>Low (0-1100 kcal/week): 1.54 (0.83, 2.84);<br>Moderate (1101-1900 kcal/week): 1.21 (0.63, 2.31);<br>High (>1900 kcal/week): 1.00<br><br>Females:<br>Low (0-900 kcal/week): 2.64 (1.28, 5.44);<br>Moderate (901-1500 kcal/week):	Age	4

Author (Year)	Study Name	Country	Time Period	Age Range	Sex	Sample Size	Activity Type	Effect Type	Effect	Covariates adjusted	NOS score
									1.17 (0.50, 2.70); High (>1500 kcal/week): 1.00		
Helmrich (1994)	The original University of Pennsylvania Alumni Health Study	United States	1962-1976	39-68	Male	5990	Recreation	RR	<500 kcal/week: 1.00; 500-999 kcal/week: 0.94 (0.53, 1.73); 1000-1499 kcal/week: 0.79 (0.43, 1.49); 1500-1999 kcal/week: 0.78 (0.43, 1.49); 2000-2499 kcal/week: 0.68 (0.36, 1.31); 2500-2999 kcal/week: 0.90 (0.51, 1.67); 3000-3499 kcal/week: 0.86 (0.48, 1.61); ≥3500 kcal/week: 0.52 (0.26, 1.07)	Age	4
Holme (2007)	Oslo Study	Norway	1972-2000	40-49	Male	6379	Recreation	OR	Sedentary/Light: 1.00; Moderate: 0.87 (0.68, 1.10); Moderately Vigorous: 0.87 (0.64, 1.17); Vigorous: 0.42 (0.17, 1.06)	Smoking, Age, Years of Education, Glucose, Triglycerides, Body Mass Index, Treated Hypertension, and Systolic Blood Pressure in 1972/3	5
Hsia (2005)	Women's Health Initiative	United States	1994-2002. Mean	50-79	Female	86708	Recreation	HR	0-2.3 MET hr/week: 1.00; 2.3-7.4 MET	Age, BMI, Alcohol, Education,	7

Author (Year)	Study Name	Country	Time Period	Age Range	Sex	Sample Size	Activity Type	Effect Type	Effect	Covariates adjusted	NOS score
	Observational Study		follow up 5.1 years						hr/week: 0.91 (0.80, 1.03); 7.5-13.9 MET hr/week: 0.80 (0.70, 0.91); 14.0-23.4 MET hr/week: 0.86 (0.75, 0.99); 23.5-143.0 MET hr/week: 0.78 (0.67, 0.91)	Smoking, Hypertension, Hypercholesterolemia, Dietary Fiber, Percent Energy from Carbohydrates	
Hu (1999)	Nurses' Health Study	United States	1986-1994	40-65	Female	70102	Recreation	RR	0-2.0 MET hr/week: 1.00; 2.1-4.6 MET hr/week: 0.84 (0.72, 0.97); 4.7-10.4 MET hr/week: 0.87 (0.75, 1.02); 10.5-21.7 MET hr/week: 0.77 (0.65, 0.91); ≥21.8 MET hr/week: 0.74 (0.62, 0.89)	Age, Time Period, Cigarette Smoking, Menopausal Status, Parental History of Diabetes, Alcohol Consumption, Hypertension, High Cholesterol, BMI	6
Hu (2001)	The Health Professional 's Follow-up Study	United States	1986-1996	40-75	Male	37918	Recreation	RR	0-5.9 MET hr/week: 1.00; 6.0-13.7 MET hr/week: 0.88 (0.71, 1.10); 13.8-24.2 MET hr/week: 0.75 (0.60, 0.94); 24.3-40.8 MET hr/week: 0.69 (0.54, 0.87); ≥40.9 MET hr/week: 0.57 (0.44, 0.74)	Excluding First 2 Years Of Follow-Up, Age, Pack-Years Smoking, Parental Family History of Diabetes, Alcohol Intake, Vit E Supplement Use	7

Author (Year)	Study Name	Country	Time Period	Age Range	Sex	Sample Size	Activity Type	Effect Type	Effect	Covariates adjusted	NOS score
Hu (2003)	Study in two Eastern provinces and in a region in Southwestern Finland plus the Helsinki capital area	Finland	1982-1998 Mean follow-up 8 years.	35-64	Both	14290	Occupational<br><br>Commuting<br><br>Recreation	HR	Light: 1.00 Moderate: 0.60 (0.45, 0.81); Active: 0.68 (0.53, 0.87)<br><br>0 Min: 1.00; 1-29 Min: 0.80 (0.62, 1.03); >=30 Min: 0.47 (0.33, 0.66)<br><br>Low: 1.00; Moderate: 0.65 (0.52, 0.82); High: 0.59 (0.40, 0.87)	Age, Sex, Study Year, SBP, Smoking, Education	7
James (1998)	The Pitt County Study in North Carolina	United States	1988-1993	25-50	Both	916	Recreation+ Occupational+Housework	OR	Inactive: 1.00 Low: 0.51 (0.20, 1.28); Moderate: 0.35 (0.12, 0.98); Strenuous: 0.65 (0.26, 1.63)	Age, Sex, Education, BMI, Waist-To-Hip Ratio	5
Jefferis (2012)	A cohort based in general practices in 24 British towns	United Kingdom	1996-2000 Mean follow up 7.1 years.	60-79	Male	2675	Recreation	HR	None: 1.00 Occasional: 0.73 (0.41, 1.31); Light: 0.49 (0.25, 0.95); Moderate: 0.50 (0.25, 1.01); Moderately	Age, Region, Social Class (Nonmanual, Manual, or Armed forces), Smoking	6

Author (Year)	Study Name	Country	Time Period	Age Range	Sex	Sample Size	Activity Type	Effect Type	Effect	Covariates adjusted	NOS score
									Vigorous: 0.58 (0.29, 1.16); Vigorous: 0.48 (0.23, 1.02)	History (Nonsmoker or Current Smoker); Alcohol Intake (none/occasional, 1–15 units/week, or ≥16 units/week), Coffee (0, 1–3, or ≥4 cups/day), Total kcal/day, Dietary fiber (g)/day, Protein (g)/day, and Carbohydrate (g)/day, total Cholesterol, HDL Cholesterol, Triglycerides, BMI	
Joseph (2010)	Tromsø Study	Norway	1994-2005 Median follow up 10.8 years.	25-98	Male	12431	Recreation	HR	Inactive: 1.69 (1.12, 2.55); Moderate: 1.03 (0.68, 1.55); Hard: 1.00	Age, BMI, Total Cholesterol, Triglycerides, HDL Cholesterol, Hypertension, Family History, Education, and Smoking	8
					Female	13737			Inactive: 1.13 (0.69, 1.83); Moderate: 0.81 (0.49, 1.35); Hard: 1.00		
Koloverou (2014)	ATTICA study	Greece	2002-2012	rathm	Both	1485	Total	OR	Inactive: 1.00; Physically Active:	Age, Sex, Years of	8

Author (Year)	Study Name	Country	Time Period	Age Range	Sex	Sample Size	Activity Type	Effect Type	Effect	Covariates adjusted	NOS score
									0.51 (0.24, 1.10)	School, Smokers vs. Non-Smokers, Family History of Diabetes, Energy Intake, Abnormal Waist Height Ratio, Fasting Blood Glucose, Hypercholesterolemia, and Hypertension	
Krishnan (2008)	The Black Women's Health Study	United States	1995-2005	21-69	Female	45668	Recreation	HR	0 hr/wk: 1.00; <1 hr/wk: 0.90 (0.82, 0.99); 1-2 hr/wk: 0.77 (0.69, 0.85); 3-4 hr/wk: 0.53 (0.45, 0.63); 5-6 hr/wk: 0.49 (0.38, 0.64); >=7 hr/wk: 0.43 (0.31, 0.59)	Age, Time Period, Family History of Diabetes, Years of Education, Family Income, Marital Status, Cigarette Use, Alcohol Use, Energy Intake, Coffee Consumption, Television Watching	6
Laaksonen (2010)	Mini-Finland Health Survey	Finland	1978-1988	40-79	Both	4517	Recreation	RR	No Exercise: 1.00; Occasional or Regular Exercise: 0.72 (0.52, 1.01)	Age, Sex	6

Author (Year)	Study Name	Country	Time Period	Age Range	Sex	Sample Size	Activity Type	Effect Type	Effect	Covariates adjusted	NOS score
	Health 2000 Survey		2000-2007	40-79	Both	4110	Recreation		No Exercise: 1.00; Occasional or Regular Exercise: 0.65 (0.40, 1.03)		
Lee (2012)	Individuals were selected from the database of the National Health Insurance Corporation (NHIC)	South Korea	1996-2005. Mean follow up 7.5 years	18+	Male	675496	Recreation	HR	0 min/week: 1.00; 1-149 min/week: 0.95 (0.93, 0.97); 150-299 min/week: 0.90 (0.87, 0.93); ≥ 300 min/week: 0.91 (0.88, 0.94)	Age, Smoking Status, Alcohol Intake, Hypertension, Parental Diabetes, Baseline Glucose, BMI	8
Longo-Mbenza (2009)	A prospective cohort of non-diabetic Central Africans	Democratic Republic Of The Congo	2004-2008 Median follow-up 50 months.	≥40 years	Both	807	Recreation and Occupation	HR	Unexposed to Physical Inactivity: 1.0; Exposed to Physical Inactivity: 3.5 (1.2, 10.7)	Waist Circumference, Systolic BP, Sex, Diabetes, Heredity, Malnutrition, Environment, Psychosocial (Stress), Lifestyle, and Westernisation Risk Factors	6
Lucke (2007)	Australian Longitudinal Study on Women's Health	Australia	1996-2003	18-75	Female	40395	Total	RR	Nil/Low: 1.07 (0.70, 1.64); Moderate/High: 1.00	BMI, Smoking, Alcohol, Education	5
Magliano (2008)	The Australian Diabetes, Obesity and Lifestyle Study	Australia	1999-2005 Mean follow up 3 years.	≥25 years	Both	5842	Recreation	OR	Inactive (0 Min/Week): 1.56 (1.12, 2.18); Insufficient (1–149 Min/Week): 1.51 (1.01, 2.25); Sufficient (150	Age, Female Sex, Waist Circumference, Smoking Status, Education, Hypertension,	8

Author (Year)	Study Name	Country	Time Period	Age Range	Sex	Sample Size	Activity Type	Effect Type	Effect	Covariates adjusted	NOS score
									Min/Week): 1.00	Family History of Diabetes, Log FPG, Hypertriglyceridemia, Low HDL Cholesterol, Total Cholesterol	
Manson (1991)	Nurses' Health Study	United States	1980-1988	34-59	Female	87253	Recreation	RR	Frequency of Vigorous Exercise:<br>0 Times/Week: 1.00;<br>1 Time/Week: 0.89 (0.72, 1.11);<br>2 Times/Week: 0.71 (0.56, 0.89);<br>3 Times/Week: 0.93 (0.75, 1.16);<br>4+ Times/Week: 0.86 (0.71, 1.04)	Age, BMI	5
Manson (1992)	The Physician's Health Study	United States	1982-1988. Mean follow up 60.2 months.	40-84	Male	21271	Recreation	RR	Frequency of Vigorous Exercise:<br>0 Times/Week: 1.00;<br>1 Time/Week: 0.78 (0.56, 1.09);<br>2-4 Times/Week: 0.68 (0.51, 0.90);<br>≥5 Times/Week: 0.71 (0.49, 1.03)	Age, BMI	4
Meisinger (2005)	The MONICA/KORA Augsburg Cohort Study	Germany	1984-1998. Mean follow up 7.4 years.	25-74	Male<br><br>Female	4069 (males)<br><br>4034 (female)	Recreation	HR	Males:<br>No Activity: 1.00<br>Low: 0.91 (0.61, 1.38);<br>Moderate: 0.76 (0.47, 1.25);<br>High: 0.83 (0.50, 1.36)	Age, Survey, Actual Hypertension, Dyslipidaemia, Parental History of Diabetes, Regular	8

Author (Year)	Study Name	Country	Time Period	Age Range	Sex	Sample Size	Activity Type	Effect Type	Effect	Covariates adjusted	NOS score
									Females:<br>No Activity: 1.0<br>Low: 0.87 (0.52, 1.45);<br>Moderate: 0.70 (0.37, 1.33);<br>High: 0.24 (0.06, 0.98)	Smoking, Alcohol Intake, Education, BMI	
Mozaffarian (2009)	Cardiovascular Health Study	United States	1989-1998	65+	Both	4883	Total daily activity	HR	< Median: 1.00;<br>≥ Median: 0.74 (0.58, 0.93)	Age, Sex, Race, Educational Level, Annual Income, Diet, Smoking, Alcohol use, BMI, waist circumference	7
Okada (2000)	The Osaka Health Survey	Japan	1981-1997	35-60	Male	6013	Recreation+ Housework	RR	Frequency of regular physical activity:<br>0 Times/Week: 1.00;<br>1-2 Times/Week: 0.80 (0.64, 0.99);<br>≥3 Times/Week: 0.55 (0.34, 0.87)	Age, BMI, Alcohol, Smoking, Blood Pressure, Parental History of Type 2 Diabetes	7
Panagiotakos (2008)	The ATTICA study	Greece	2001-2006	18+	Both	1806	Total	OR	Inactive: 1.00;<br>Active: 0.62 (0.35, 1.02)	Age, Waist Circumference, Fasting Blood Glucose, Family History of Diabetes	7
Rathmann (2009)	KORA S4⁄ F4 cohort study	Germany	1999-2008 Mean	55-74	Both	887	Recreation	OR	Inactive During Leisure Time: 1.3 (0.8, 2.0);	Age, Sex	5

Author (Year)	Study Name	Country	Time Period	Age Range	Sex	Sample Size	Activity Type	Effect Type	Effect	Covariates adjusted	NOS score
			follow up 4.5 years.						Active During Leisure Time: 1.0		
Reis (2011)	National Institutes of Health (NIH)-AARP Diet and Health Study.	United States	1995-2006 1999-2008	50-71	Male<br><br>Female	114996<br><br>92483	Total	OR	Regular Physical Activity? No: 1.00; Regular Physical Activity? Yes: 0.76 (0.73, 0.79)<br><br>Regular Physical Activity? No: 1.00; Regular Physical Activity? Yes: 0.77 (0.73, 0.82)	Age, Race/Ethnicity, Educational Attainment, Marital Status, BMI, Diet Score, Smoking, Moderate Alcohol Consumption, Use of Hormone Replacement (for women)	6
Shi (2013)	Shanghai Men's Health Study (SMHS)	China	2002-2011	40-74	Male	51464	Recreation	HR	None: 1.00; <1.2 MET hr/week: 0.80 (0.65, 0.97); 1.2-3.0 MET hr/week: 0.89 (0.74, 1.07); ≥3.0 MET hr/week: 0.91 (0.76, 1.08)	Age at Interview, Energy Intake, Smoking, Alcohol Consumption, Education Level, Occupation, Income Level, Hypertension, Family History of Diabetes, BMI, and Waist Hip ratio	8
Siegel (2009)	Physicians' Health	United States	1982-2006	40-84	Male	20757	Recreation	HR	Rarely/Never: 1.00;	Age, Alcohol Use, Smoking	5

Author (Year)	Study Name	Country	Time Period	Age Range	Sex	Sample Size	Activity Type	Effect Type	Effect	Covariates adjusted	NOS score
	Study		Median follow up 23.1 years.						1-3/Mo: 0.84 (0.72, 0.98); Once/Wk: 0.81 (0.70, 0.93); 2-4/Wk: 0.69 (0.61, 0.79); >=5 Times/Wk: 0.58 (0.48, 0.69)	Status, History of High Cholesterol, History of Hypertension, WHO BMI Group	
Simonsick (1993)	Three sites of the Established Populations for Epidemiologic Studies of the Elderly (EPESE)	United States	1982-1989	65-99	Both	2188<br><br>2537<br><br>2714	Recreation and Household	OR	Boston:<br>Inactive: 1.00<br>Moderately Active: 0.85 (0.50, 1.45);<br>Highly Active: 1.11 (0.53, 2.30)<br><br>Iowa:<br>Inactive: 1.00<br>Moderately Active: 0.76 (0.42, 1.38);<br>Highly Active: 1.51 (0.83, 2.75)<br><br>New Haven:<br>Inactive: 1.00<br>Moderately Active: 1.54 (0.68, 3.49);<br>Highly Active: 0.59 (0.20, 1.77)	Age, Sex, Education, Work Status, Smoking, Respiratory Symptoms, Myocardial Infarction, Stroke, Diabetes, Angina, Self-Rated Health, and Modified Depression Score.	5
Steinbrecher (2012)	Hawaii component of the Multiethnic Cohort	United States	1993-2007	45-75	Male	35976	Recreation	HR	Strenuous Sport H/Wk, Never: 1.00;<br>Strenuous Sport H/Wk, 1/2-1: 0.94 (0.87, 1.02);	Age, Ethnicity, Education, BMI	6

Author (Year)	Study Name	Country	Time Period	Age Range	Sex	Sample Size	Activity Type	Effect Type	Effect	Covariates adjusted	NOS score
									Strenuous Sport H/Wk, 2-3: 0.85 (0.77, 0.94); Strenuous Sport H/Wk, 4+: 0.80 (0.72, 0.88)		
							Transportation		Vigourous Work H/Wk, Never: 1.00; Vigourous Work H/Wk, 1/2-1: 0.91 (0.85, 0.98); Vigourous Work H/Wk, 2-3: 0.91 (0.83, 1.00); Vigourous Work H/Wk, 4+: 0.84 (0.77, 0.92)		
					Female	38937	Recreation		Strenuous Sport H/Wk, Never: 1.00; Strenuous Sport H/Wk, 1/2-1: 1.00 (0.91, 1.09); Strenuous Sport H/Wk, 2-3: 0.85 (0.75, 0.96); Strenuous Sport H/Wk, 4+: 0.67 (0.57, 0.79)		
							Transportation		Vigourous Work H/Wk, Never: 1.00; Vigourous Work H/Wk, 1/2-1: 1.03 (0.95, 1.12); Vigourous Work		

Author (Year)	Study Name	Country	Time Period	Age Range	Sex	Sample Size	Activity Type	Effect Type	Effect	Covariates adjusted	NOS score
									H/Wk, 2-3: 0.99 (0.87, 1.13); Vigourous Work H/Wk, 4+: 0.91 (0.78, 1.06)		
Stringhini (2012)	Whitehall II study	United Kingdom	1991-2009 Mean follow up 14.2 years.	35-55	Both	7237	Total	HR	Inactive: 1.33 (1.13, 1.56); Moderately Active : 1.25 (1.04, 1.50); Active: 1.00	Age, Sex, Ethnicity.	4
Sun (2009)	Taiwan MJ Longitudinal health-check-up-based Population Database (MJLPD)	Taiwan	1998-2006	35-74	Both	73961	Recreation	HR	Sport Time, Seldom: 1.00; Sport Time >=1H/Wk: 1.11 (1.01, 1.23)	Sex, Education, Age, Family History of Type 2 Diabetes, Current Smoking, Hypertension, BMI, WC, FPG	7
Tsai (2015)	Taiwan Longitudinal Survey on Aging (TLSA)	Taiwan	1999-2003	53-99	Both	2995	Recreation	OR	Inactive: 1.00; Moderate: 0.95 (0.56, 1.58); Physical Activity, High: 0.59 (0.43, 0.80)	Sex, age, formal education(year), smoking, alcohol drinking, betel quid chewing, BMI, Instrumental activities of daily living, Hypertension, Heart Disease, Chronic Kidney	6

Author (Year)	Study Name	Country	Time Period	Age Range	Sex	Sample Size	Activity Type	Effect Type	Effect	Covariates adjusted	NOS score
										Disease, Gout, Center for Epidemiologic Studies, Depression Scale	
Villegas (2006)	The Shanghai Women's Health Study (SWHS)	China	1997-2004. Follow up 4.6 years	40-70	Female	64130	Recreation	RR	0 MET hr/Day: 1.00;<br><0.8 MET hr/Day: 0.89 (0.76, 1.03);<br>0.8-1.99 MET hr/Day: 0.99 (0.85, 1.15);<br>>1.99 MET hr/Day: 0.83 (0.70, 0.97)	Age, Kcal/Day, Education, Income, Occupation, Smoking, Alcohol, Hypertension	7
Waki (2005)	Japan Public Health Center-based prospective study on cancer and cardiovascular diseases (JPHC Study) Cohort I	Japan	1990-2000	40-59	Male<br><br>Female	12913<br><br>15980	Recreation	OR	Inactive: 1.00;<br>Active: 0.90 (0.73, 1.12)<br><br>Inactive: 1.0<br>Active: 1.06 (0.82, 1.37)	Age, BMI, Smoking Status, Alcohol Intake, Family History, Hypertension	6
Waller (2010)	The Finnish Twin Cohort	Finland	1976-2004	18+	Male<br><br>Female	9842 (males)<br><br>10645(females)	Recreation+ Commuting	HR	Males:<br><0.59 MET hr/Day: 1.00;<br>≥0.59 MET hr/Day: 0.49 (0.27, 0.87)<br><br>Females:	BMI	7

Author (Year)	Study Name	Country	Time Period	Age Range	Sex	Sample Size	Activity Type	Effect Type	Effect	Covariates adjusted	NOS score
									<0.59 MET hr/Day: 1.00; ≥0.59 MET Hr/Day: 0.59 (0.36, 0.96)		
Wannamethee (2000)	The British regional heart study	United Kingdom	1978-1995. Mean follow up 16.8 years.	40-59	Male	5159	Recreation+ Commuting	RR	Inactive: 1.00; Occasional: 0.81 (0.51, 1.29); Light: 0.86 (0.54, 1.41) Moderate: 0.66 (0.38, 1.17); Moderately Vigorous/Vigorous : 0.69 (0.39, 1.22)	Age, Smoking, Alcohol, Social Class, BMI, Preexisting CHD, Insulin, Diastolic Blood Pressure, Triglyceride, HDL Cholesterol, Gamma-Glutamyltransferase	8
Weinstein (2004)	Women's Health Study (WHS)	United States	1992-1999. Mean follow up 6.9 years	45+	Female	37878	Recreation	HR	0-199 kcal/week: 1.00; 200-599 kcal/week: 0.91 (0.79, 1.06); 600-1499 kcal/week: 0.86 (0.74, 1.01); ≥1500 kcal/week: 0.82 (0.70, .97)	Age, Family History of Diabetes, Alcohol, Smoking, Hormone Therapy Use, Hypertension, High Cholesterol, Dietary Factors, Randomized Women's Health Study Treatment Group, BMI	7
Williams (2013)	National Runners'	United States	1998-2006		Both	33060	Recreation	HR	Running, <1: 1.00; Running, 1-2:	Age, Sex, Race,	4

Author (Year)	Study Name	Country	Time Period	Age Range	Sex	Sample Size	Activity Type	Effect Type	Effect	Covariates adjusted	NOS score
	Health Study II and the National Walkers' Health Study								0.57(0.38, 0.87); Running, 2-3: 0.55(0.35,0.87); Running, 3-4: 0.52 (0.33, 0.82) Running, >= 4: 0.31 (0.17, 0.54)<br><br>Walking, <1: 1.00; Walking, 1-2: 0.65 (0.53, 0.81); Walking, 2-3: 0.55 (0.38, 0.78);	Smoking, Prior Coronary Heart Disease, Intakes of Red Meat, Fruit, Alcohol	
Xu (2014)	Two community-based prospective cohort studies	China	2004-2010 Mean follow up 3 years.	35-100	Both	5659	Total	OR	Insufficient: 1.00; Sufficient: 0.43 (0.27, 0.68)	Age, Gender, Educational Attainment, Family History, Hypertension, Body Weight Status, Cigarette Smoking, Alcohol Drinking, TV Viewing, Vegetables Intake, Meat Intake	7

Table D. Characteristics and quality assessment of studies included in the meta-regression analysis for the association between physical activity and ischemic heart disease

**Author (Year)**	**Study Name**	**Country**	**Time Period**	**Age Range**	**Sex**	**Sample Size**	**Activity Type**	**Effect Type**	**Effect**	**Covariates adjusted**	**NOS score**
Akesson (2007)	Swedish Mammography Cohort linked to Swedish Hospital Discharge	Sweden	1997-2003. Mean follow up 6.2 years	48-83	Female	24444	Recreation	RR	<1 Hr/Week: 1.40 (1.10, 1.47); ≥ 1Hr/Week: 1.00	Age, Education, Family History of MI, High Cholesterol, Hypertension, Use of Hormone Therapy, Use of Aspirin, Total Energy Intake, Healthy And Alcohol Dietary Patterns, Smoking, Waist To Hip Ratio	8
Allesoe (2014)	Danish Nurse Cohort Study	Denmark	1993-2008 Mean follow up 7.5 years.	45-64	Female	12093	Occupation<br><br>Recreation	HR	Sedentary: 1.13 (0.86, 1.49); Moderate: 1.00 Vigorous: 1.34 (1.08, 1.66)<br><br>Sedentary: 1.50 (1.04, 2.16); Moderate: 1.12 (0.96, 1.45); Vigorous: 1.0	Age, Family History of IHD, Diabetes, BMI, Smoking, Alcohol Consumption, Work Pressure, Job Influence, Shift Work, Work Hours Per Week	6
Armstrong (2015)	the Million Women Study	United Kingdom	1998-. Mean follow up 9	50-64	Female	1119239	total	RR	0-40 Per Week: 1 (0.98, 1.03); > 40-80 Per Week: 0.92 (0.90, 0.94);	BMI by Age, Smoking-by Age, Alcohol-By-Age,	7

Author (Year)	Study Name	Country	Time Period	Age Range	Sex	Sample Size	Activity Type	Effect Type	Effect	Covariates adjusted	NOS score
			years						> 80 Per Week : 0.96 (0.93, 0.98)	Stratified by SES and Region	
Batty (2002)	Whitehall study	United Kingdo m	1967-1995	40-64	Male	6408	Recreation	RR	Inactive: 1.08 (0.90, 1.30); Moderate: 0.94 (0.80, 1.10); Active: 1.00	Excluding deaths in first five years of follow up, Age, Employment Grade, Systolic BP, Cholesterol, Smoking, BMI, FEV in One Second, Disease At Study Entry	5
Bijnen (1998)	The Zutphen Study	Netherla nds	1985-1995	64-84	Male	802	Recreation+ Commuting	RR	Lowest Tertile: 1.00; Middle Tertile: 0.63 (0.38, 1.05); Highest Tertile: 0.85 (0.51, 1.44)	Age, Baseline CHD, Smoking, Alcohol	5
Calling (2006)	The Malmo Diet and Cancer Study	Sweden	1991-. Mean follow up 7.6 years	45-73	Both	26942	recreation	RR	Low Active: 1.00; Active: 0.66 (0.57, 0.77)	Age, Sex	5
Chen (1999)	Statistics Canada's National Population Health Survey	Canada	1994-1997	20+	Both	7158	Recreation	OR	Sedentary (Irregular Physical Activity): 5.00 (1.84, 13.59); Light (<1.5 Kcal/Kg/Day During Regular Physical Exercise): 3.7 (1.26, 10.67); Moderate (1.5-2.9 Kcal/Kg/Day During Regular Physical Activity): 1.00;	Age, Sex, Education, Household Income, Activity Limitation, Smoking, High Blood Pressure, BMI	6

Author (Year)	Study Name	Country	Time Period	Age Range	Sex	Sample Size	Activity Type	Effect Type	Effect	Covariates adjusted	NOS score
									Active (3 Kcal/Kg/Day During Regular Physical Activity): 1.3; (0.41, 3.89)		
Chiuve (2006)	Health Professionals Follow-up Study	United States	1986-2002	40-75	Male	42847	Recreation	RR	Moderate to Vigorous exercise:<br>0 hr/week: 1.22 (1.06, 1.40);<br>0.1-1.5 hr/week: 1.05 (0.90, 1.24);<br>1.5-3.5 hr/week: 1.08 (0.92, 1.27);<br>3.5-6.0 hr/week: 0.92 (0.79, 1.11);<br>≥6.0 hr/week: 1.00	Age, Family History of MI Before 60, Aspirin, Anti-hypertensives, Hypercholesterolemia, Hypertension, Other Lifestyle Factors	7
Chomistek (2013)	Women's Health Initiative Observational Study	United States	1994-2010	50-79	Female	71018	Recreation	HR	Inactive: 1.43 (1.25, 1.63);<br>Low: 1.28 (1.13, 1.45);<br>Medium: 1.20 (1.06, 1.36);<br>High: 1.00	Age, Sedentary Time, Race, Education, Income, Marital Status, Smoking, Family History of MI, Depression, Alcohol Intake, Hours of Sleep, Intake of Total Calories, Saturated Fat, Fiber, BMI, History of Hypertension, Diabetes, High Cholesterol at Baseline	8

Author (Year)	Study Name	Country	Time Period	Age Range	Sex	Sample Size	Activity Type	Effect Type	Effect	Covariates adjusted	NOS score
Donahue (1988)	The Honolulu Heart Program	United States	1965-.	45-65<br><br>65-69	Male<br><br>Male	7221<br><br>423	Total<br><br>Total	RR	Inactive: 1.00<br>Active: 0.69(0.53, 0.88)<br><br>Inactive: 1.00<br>Active: 0.43(0.19, 0.99)	Age, Alcohol Intake, Cigarette Use	6
Eaton (1995)	Cohort Analytic Study of Israeli government employees (The Israeli Ischemic Heart Disease Study)	Israel	1963-1986. Mean follow-up 11.5 years.	40+	Male	8463	Recreation+Commuting<br><br>Occupational	RR	Sedentary: 1.00;<br>Light: 0.79 (0.63, 0.99);<br>Light Daily: 0.73 (0.59, 0.89);<br>Heavy: 0.71 (0.52, 0.98)<br><br>Sitting: 1.00;<br>Standing: 0.99 (0.75, 1.18); Walking: 0.94 (0.78, 1.12); Physical Labor: 0.87 (0.67, 1.10)	Age	7
Folsom (1997)	The ARIC study	United States	1987-1995	45-64	Male<br><br>Female	6188 (males)<br><br>7852 (females)	Recreation	RR	Males:<br>Quartile 1 (Low): 1.00;<br>Quartile 2: 1.15 (0.79, 1.68);<br>Quartile 3: 1.03 (0.68, 1.54);<br>Quartile 4 (High): 0.83 (0.56, 1.23)<br>Females:<br>Quartile 1 (Low): 1.00;<br>Quartile 2: 0.99 (0.58, 1.67);<br>Quartile 3: 0.64 (0.32, 1.27);	Age, Education Level, Smoking, Alcohol, Diabetes, Waist/Hip Ratio, Total Cholesterol, HDL-Cholesterol, Systolic BP, Antihypertensive Medication Use,	8

Author (Year)	Study Name	Country	Time Period	Age Range	Sex	Sample Size	Activity Type	Effect Type	Effect	Covariates adjusted	NOS score
									Quartile 4 (High): 0.72 (0.37, 1.38)	Fibrinogen, Race, Aric Field Center + Hormone Replacement Therapy for Females	
Gulsvik (2012)	The Bergen Clinical Blood Pressure Survey	Norway	1965-2007. Mean follow up 21 years.	22-75	Both	5653	recreation and transportation	HR	No/Low: 1.00; Moderate: 0.77 (0.66, 0.90); High: 0.66 (0.52, 0.83)	Age, Sex	6
Haapanen (1997)	Census data of a medium-size industrial town and two rural municipalities in northeastern Finland	Finland	1980-1990	35-63	Male	842 (males)<br><br>963 (females)	Recreation+Household+Commuting	RR	Males:<br>Low (0-1100 kcal/week): 1.98 (1.22, 3.23);<br>Moderate (1101-1900 kcal/week): 1.33 (0.78, 2.27);<br>High (>1900 kcal/week): 1.00<br><br>Females:<br>Low (0-900 kcal/week): 1.25 (0.72, 2.15);<br>Moderate (901-1500 kcal/week): 0.73 (0.38, 1.39);<br>High (>1500 kcal/week): 1.00	Age, Smoking	5
Harari (2015)	The CORDIS Study (Cardiovascular Occupational Risk Factor Determination in Israel	Israel	1985-2007	20-70	Male	4819	Occupation<br><br>Recreation	HR	None-To-Mild: 1.00; Moderate-To-Hard: 1.35 (0.94, 1.95)<br><br>Less or None: 1.00; 30 Min at Least Twice a Week: 0.64	Age at Screening, Socioeconomic Status (Number Of People/Room), Educational Status,	6

Author (Year)	Study Name	Country	Time Period	Age Range	Sex	Sample Size	Activity Type	Effect Type	Effect	Covariates adjusted	NOS score
	Study)								(0.40, 1.02)	Father's Country of Origin, Body Mass Index, Cholesterol, High-Density Lipoprotein Cholesterol, Hypertension, Diabetes, Smoking, Coffee Consumption, Alcohol Consumption, Maintaining a Special Diet, Shift Work	
Hillsdon (2004)	The OXCHECK study	United Kingdom	1989-2001	35-64	Both	7704	Recreation	RR	Frequency of vigorous exercise: <1 time/month: 1.00; 1-3 times/month: 1.15 (0.42, 3.17); 1 time/week: 0.37 (0.12, 1.17); ≥2 times/week: 0.50 (0.20, 1.23)	Age, Sex, Smoking Status, Alcohol, Pre-Existing Disease, Social Class	6
Holtermann (2012)	The Copenhagen Male Study	Denmark	1970-2001.	40-59	Male	4774	Recreation	HR	Low: 1.00; Medium: 0.73 (0.59, 0.89); High: 0.62 (0.43, 0.90)	Age, Lifestyle, Clinical Factors, Psychosocial Stress At Work and Leisure, Number of Work Hours and Social Class	6
Hu	Six surveys	Finland	1972-	25-64	Male	22877	Recreation	HR	Males:	Age, Study	7

Author (Year)	Study Name	Country	Time Period	Age Range	Sex	Sample Size	Activity Type	Effect Type	Effect	Covariates adjusted	NOS score
(2007)	in five geographic areas of Finland		2003. Mean follow up 15.5 years.		 <br>Female	(males)<br><br>24963 (females)	 <br><br><br><br><br><br><br><br><br><br><br><br><br><br><br>Commuting<br><br><br><br><br><br><br><br><br><br><br><br><br><br><br><br>Occupational		Low: 1.00;<br>Moderate: 0.95 (0.88, 1.02);<br>High: 0.84 (0.74, 0.95)<br><br>Females:<br>Low: 1.00; Moderate: 0.84 (0.76, 0.94);<br>High: 0.74 (0.60, 0.93)<br><br>Males:<br>0 Min/Day: 1.00;<br>1-29 Min/Day: 0.96 (0.88, 1.04);<br>>=30 Min/Day: 0.95 (0.87, 1.05)<br><br>Females:<br>0 Min/Day: 1.00;<br>1-29 Min/Day: 0.87 (0.77, 0.98);<br>>=30 Min/Day: 0.72 (0.63, 0.83)<br><br>Males:<br>Low: 1.00;<br>Moderate: 0.86 (0.78, 0.96);<br>High: 0.87 (0.81, 0.95)<br><br>Females:<br>Low: 1.00;	Year, Education, Alcohol, Smoking, BMI, Systolic BP, Cholesterol, History Diabetes	

Author (Year)	Study Name	Country	Time Period	Age Range	Sex	Sample Size	Activity Type	Effect Type	Effect	Covariates adjusted	NOS score
									Moderate: 0.71 (0.63, 0.81);<br>High: 0.77 (0.68, 0.87)		
Inoue (2008)	The JPHC study	Japan	1995-2005	45-74	Male	39183 (males)<br><br>43851 (females)	Total daily activity	HR	Males:<br>Lowest Quartile: 1.00;<br>Second Quartile: 0.99 (0.72, 1.36);<br>Third Quartile: 0.69 (0.48, 0.99);<br>Highest Quartile: 0.78 (0.56, 1.09)<br><br>Females:<br>Lowest Quartile: 1.00;<br>Second Quartile: 0.76 (0.66, 0.88);<br>Third Quartile: 0.65 (0.55, 0.77);<br>Highest Quartile: 0.60 (0.49, 0.72)	Age, Geographic Area, Occupation, History Of Diabetes, Smoking, Alcohol, BMI, Total Energy Intake, Leisure-Time Sports or Physical Exercise, Excluding Deaths within First Three Years	7
Jefferis (2014)	British Regional Heart Study	United Kingdom	1996-2010. Mean follow up 7 years.	40-59	Male	3320	recreation	HR	None: 1.00;<br>Occasional: 0.53 (0.35, 0.81);<br>Light: 0.50 (0.32, 0.78);<br>Moderate: 0.57 (0.35, 0.92); Moderately Vigorous and Vigorous: 0.51 (0.33, 0.78)	Age, Region, Alcohol Intake, Smoking History, Plasma Vitamin C, Social Class, Total Cholesterol, High-Density Lipoprotein Cholesterol, Triglycerides, Systolic Blood Pressure, Waist	7

Author (Year)	Study Name	Country	Time Period	Age Range	Sex	Sample Size	Activity Type	Effect Type	Effect	Covariates adjusted	NOS score
										Circumference, Forced Expiratory Volume in 1 Second, Estimated Glomerular Filtration Rate, Depression, Diabetes Mellitus, N-Terminal Pro-Brain Natriuretic Peptide	
Kaprio (2000)	The Finnish twin cohort	Finland	1975-1995	25-69	Male	8205	Recreation+Commuting	RR	Sedentary: 1.00; Occasional Exercisers: 0.84 (0.70, 1.01); Conditioning Exercisers: 0.68 (0.50, 0.92)	Age, BMI, Smoking, Hypertension, Diabetes	7
Lakka (1994)	The Kuopio Ischemic Heart disease Risk Factor Study	Finland	1984-1991. Mean follow up 4.9 years.	42-60	Male	1453	Recreation	HR	<0.7 hr/week: 1.00; 0.7-2.2 hr/week: 1.11 (0.58, 2.12); >2.2 hr/week: 0.31 (0.12, 0.85)	Age, Year Of Examination	5
Leon (1997)	Participants of the Multiple Risk Factor Intervention Trial (MRFIT)	United States	1974-1990. Mean follow up 7 years.	35-57	Male	12138	Recreation	RR	0-9 Min/Day: 1.00; 10-36 Min/Day: 0.75 (0.54, 0.96); 37-75 Min/Day: 0.81 (0.64, 1.04); 76-359 Min/Day: 0.75 (0.59, 0.96)	Age, Intervention Group, Education, Cigarettes Per Day, Serum Cholesterol, Diastolic BP, BMI	6
Li (2006)	Nurses' Health Study	United States	1980-2000	34-59	Female	88393	Recreation	RR	< 1 hr/week: 1.43 (1.26, 1.63);	Age, Smoking Status,	7

Author (Year)	Study Name	Country	Time Period	Age Range	Sex	Sample Size	Activity Type	Effect Type	Effect	Covariates adjusted	NOS score
									1 - 3.49 hr/week: 1.34 (1.18, 1.51); ≥3.5 hr/week: 1.00	Parental History of Coronary Heart Disease, Postmenopausal Status, Hormone Use, Alcohol Consumption, Aspirin Use, BMI	
Mannsverk (2015)	The Tromsø Study	Norway	1994-2010.	25-100	Both	29582	Recreation	HR	<1 Hour Per Week: 1.0; >=1 Hour Per Week: 0.84 (0.74, 0.94)	Gender and Age	6
Meisinger (2007)	The MONICA Augsburg cohort study	Germany	1984-2002	45-74	Male Female	3501 (males) 3475 (females)	Recreation	HR	Males: No Sports Activities in Leisure Time: 1.00; Low Level of Sports Activities in Leisure Time: 1.01 (0.73, 1.40); Moderate Level of Sports Activities in Leisure Time: 0.78 (0.56, 1.10); High Level of Sports Activities in Leisure Time: 0.84 (0.59, 1.18) Females: No Sports Activities in Leisure Time: 1.00; Low Level of Sports Activities in Leisure Time: 1.00 (0.56, 1.78); Moderate Level of Sports Activities in Leisure Time: 0.49	Age, Baseline Survey, Actual Hypertension, Dyslipidemia, History of Diabetes, Smoking, Alcohol, Parental History of MI, Education, BMI	9

Author (Year)	Study Name	Country	Time Period	Age Range	Sex	Sample Size	Activity Type	Effect Type	Effect	Covariates adjusted	NOS score
									(0.24, 1.00)<br>High Level of Sports Activities in Leisure Time: 0.21 (0.05, 0.87)		
Menotti (2015)	the Italian Rural Areas of the Seven Countries Study of Cardiovascular Diseases	Italy	1960-2010.	40-59	Male	1585	occupation	HR	Sedentary (Mean Estimated Energy Expenditure=2500 Kcal): 1.00;<br>Moderate (Mean Estimated Energy Expenditure=2700 Kcal): 0.68 (0.49, 0.94);<br>Vigorous (Mean Estimated Energy Expenditure=3100 Kcal): 0.67 (0.50, 0.89)	Cigarette Smoking, Diet	6
Morris (1990)	Male executive officers aged 45-64 in the Department of Health and Social Security and in Inland Revenue throughout Britain	United Kingdom	1976-1986. Mean follow up 9 years and 4 months.	45-64	Male	9376	Recreation+Commuting+Housework	RR	Risk of IHD incidence:<br>No Vigorous Aerobic Exercise : 1.00;<br>Residual Vigorous Aerobic Exercise: 0.85 (0.68, 1.08);<br>Next Lesser Degree of This: 0.76 (0.56, 1.01);<br>Frequent Vigorous Aerobic Exercise: 0.35 (0.21, 0.57)<br><br>Risk of IHD mortality:<br>No Vigorous Aerobic Exercise: 1.00;<br>Residual Vigorous Aerobic Exercise: 0.88 (0.66, 1.17);<br>Next Lesser Degree		5

Author (Year)	Study Name	Country	Time Period	Age Range	Sex	Sample Size	Activity Type	Effect Type	Effect	Covariates adjusted	NOS score
									of This: 0.78 (0.54, 1.12);<br>Frequent Vigorous Aerobic Exercise: 0.34 (0.18, 0.66)		
Paffenbarger (1978)	Harvard Alumni	United States	1962-1972	35-74	Male	16936	Recreation+Commuting	RR	<2000 kcal/week: 1.64<br>≥2000 kcal/week: 1.0	Age	5
Pedersen (2008)	The Copenhagen City Heart Study	Denmark	1981-2001	20+	Male<br><br>Female	5272 (males)<br><br>6624 (females)	Recreation	HR	Males:<br>Inactive: 1.00;<br>Low: 0.68 (0.55, 0.83);<br>Moderate/High: 0.71 (0.58, 0.83)<br><br>Females:<br>Inactive: 1.00;<br>Low: 0.75 (0.60, 0.92);<br>Moderate/High: 0.72 (0.57, 0.92)	Age, Smoking, BMI, Education, Marital Status, Diabetes, Alcohol Intake, HDL-cholesterol	7
Qvist (1996)	Data from Nation-wide Swedish Level of living Survey and the national Cause of Death Register	Sweden	1980-1990	45-74	Male<br><br>Female	2546 (males)<br><br>2760 (females)	Recreation	RR	Males:<br>Inactivity: 1.26 (0.90, 1.80);<br>Activity: 1.00<br><br>Females:<br>Inactivity: 1.35 (0.80, 2.20);<br>Activity: 1.00	Age, Blood Pressure, Weight Index, Smoking	7
Rosengren (1997)	The Multifactor Primary Prevention Study	Sweden	1970-1993. Mean follow up 20 years	47-55	Male	7142	Recreation	RR	Sedentary: 1.00;<br>Moderately Active: 0.84 (0.71, 1.00);<br>Regular Exercise (Includes Men Who Did Athletic Sports): 0.84 (0.73, 0.96)	Age, Diastolic BP, Serum Cholesterol, Smoking, Alcohol Abuse, BMI, Diabetes, Manual vs Non-Manual	8

Author (Year)	Study Name	Country	Time Period	Age Range	Sex	Sample Size	Activity Type	Effect Type	Effect	Covariates adjusted	NOS score
										Occupational Class	
Salonen (1982)	Random sample from the population of Eastern Finland	Finland	1972-1978. Mean follow-up 3 years.	30-59	Male<br><br>Female	3978 (males)<br><br>3688 (females)	Recreation+Commuting<br><br>Occupational	RR	Males:<br>Low: 1.20 (0.90, 1.50);<br>High: 1.00<br><br>Females:<br>Low: 1.50 (0.90, 2.50);<br>High: 1.00<br><br>Males:<br>Low: 1.50 (1.20, 2.00);<br>High: 1.00<br><br>Females:<br>Low: 2.40 (1.50, 3.70);<br>High: 1.00	Age, Serum Cholesterol, Diastolic BP, BMI, Daily Number of Tobacco Products	7
Sesso (2000)	Harvard Alumni Health Study	United States	1977-1993	39-88	Male	12516	Recreation+Commuting	RR	<2100 kJ/week: 1.00;<br>2100-4199 kJ/week: 0.90 (0.79, 1.03);<br>4200-8399 kJ/week: 0.81 (0.71, 0.92);<br>8400-12599 kJ/week: 0.80 (0.69, 0.93);<br>≥12600 kJ/week: 0.81 (0.71, 0.94)	Age, BMI, Alcohol, Hypertension, Diabetes Mellitus, Smoking, Early Parental Death (<65 Years)	5
Slattery (1989)	The US Railroad study	United States	1957-1977	40-60	Male	2548	Recreation	HR	≤250 kcal/week: 1.28 (0.99, 1.63);<br>251-1000 kcal/week: 1.11 (1.00, 1.23);<br>1001-1999 kcal/week: 1.05 (1.00, 1.11);<br>≥2000 kcal/week: 1.00	Age, Systolic Blood Pressure, Serum Cholesterol, Smoking	7
Sobolski (1987)	Employed men (40-55	Belgium	1976-1983	40-55	Male	2109	Recreation	RR	Quartile 1 (Low): 0.69 (0.20, 2.20);		5

Author (Year)	Study Name	Country	Time Period	Age Range	Sex	Sample Size	Activity Type	Effect Type	Effect	Covariates adjusted	NOS score
	yrs) in selected factories						 <br><br><br><br><br><br>Occupational		Quartile 2: 1.00 (0.36, 2.90);<br>Quartile 3: 0.79 (0.25, 2.40);<br>Quartile 4 (High): 1.00<br><br>Quartile 1 (Low): .82 (0.28, 2.30); Quartile 2: 0.50 (0.14, 1.60);<br>Quartile 3: 0.81 (0.28, 2.30);<br>Quartile 4 (High): 1.00		
Sundquist (2005)	The Swedish Annual Level-of-Living Survey data linked to the Swedish National Hospital Discharge Register (SALLS)	Sweden	1988-2000. Mean follow up 11.7 years.	35-74	Both	5191	Recreation	HR	None: 1.0<br>Occasionally: 0.76 (0.55, 1.07);<br>1-2 times/week: 0.74 (0.53, 1.04);<br>Vigorously at Least Twice per Week: 0.59 (0.37, 0.95)	Age, Sex, Income, Smoking, BMI	7
Tamosiunas (2014)	Five general population surveys in Kaunas, Lithuania	Lithuania	1983-2011 Mean follow up 13.3 years.	45-64	Male<br><br><br><br><br><br><br><br><br>Female	2310<br><br><br><br><br><br><br><br><br>2579	Recreation<br><br><br><br><br><br><br><br><br>Recreation	HR	Inactive (<2.0 Hours/Week): 1.38 (0.89, 2.13);<br>Intermediate (2-6.99 Hours/Week): 1.05 (0.78, 1.42); Active (≥7 Hours/Week): 0.69 (0.52, 0.92)<br><br><br>Inactive (<2.0 Hours/Week): 1.47 (0.71, 3.05);<br>Intermediate (2-6.99 Hours/Week): 0.83	Age, Education, Alcohol Intake Frequency, Study Survey Year, Smoking Status, Body Mass Index, Total Cholesterol Level, Blood Pressure, Fasting	7

Author (Year)	Study Name	Country	Time Period	Age Range	Sex	Sample Size	Activity Type	Effect Type	Effect	Covariates adjusted	NOS score
									(0.50, 1.39); Active (≥7 Hours/Week): 0.82 (0.52, 1.28)	Glucose Level, Antihypertensive Treatment, Hypoglycemic Therapy, Lipid Lowering Treatment	
Tanasescu (2002)	The Health Professionals ' Follow-up Study (HPFS)	United States	1986-1998 Mean follow-up 6 years.	40-75	Male	44452	Recreation	RR	0-6.32 MET-h/wk: 1.00; 6.33-14.49 MET-h/wk: 0.93 (0.80, 1.06); 14.5-25.08 MET-h/wk: 0.90 (0.78, 1.05); 25.09-41.98 MET-h/wk: 0.87 (0.71, 1.01); 41.99 MET-h/wk: 0.74 (0.63, 0.87)	Alcohol, Smoking, Family History of MI, Nutrient Intake, Diabetes, High Cholesterol, Hypertension, BMI	7
Wagner (2002)	The Prospective Epidemiological Study of Myocardial Infarction	United Kingdom, France	1991-1996	50-59	Male	9758	Recreation	RR	Lowest: 1.00; Middle: 0.73 (0.51, 1.05); Highest: 0.66 (0.46, 0.96)		6
Wannamethee (2000)	The British Regional Heart Study	United Kingdom	1978-1995. Mean follow up 16.8 years.	40-59	Male	5159	Recreation+Commuting	RR	Inactive: 1.00; Occasional: 0.71 (0.54, 0.93); Light: 0.79 (0.61, 1.05); Moderate: 0.49 (0.55, 0.68); Moderately Vigorous/Vigorous: 0.89 (0.66, 1.18)	Age, Smoking, Alcohol, Social Class, BMI, Preexisting CHD, Insulin, Diastolic BP, Triglyceride, HDL Cholesterol,	7

Author (Year)	Study Name	Country	Time Period	Age Range	Sex	Sample Size	Activity Type	Effect Type	Effect	Covariates adjusted	NOS score
										Urate, Heart Rate	
Weinstein (2008)	The Women's Health Study	United States	1992-2004. Mean follow up 10.9 years.	45+	Female	38987	Recreation+Commuting	RR	0-199 kcal/week: 1.00; 200-599 kcal/week: 0.88 (0.73, 1.06); 600-1499 kcal/week: 0.78 (0.64, 0.94); ≥1500 kcal/week: 0.78 (0.63, 0.97)	Age, Treatment Randomization, Parental History MI, Alcohol, Smoking, Hormone Replacement Therapy, Dietary Factors	7
Weller (1998)	the Canada Fitness Survey (CFS)	Canada	1981-1988	30+	Female	6620	Total daily activity	OR	≥0 kcal/kg/day: 1.00; ≥3.9 kcal/kg/day: 0.95 (0.60, 1.51); ≥7.0 kcal/kg/day: 0.45 (0.25, 0.83); ≥11.3 kcal/kg/day: 0.61 (0.32, 1.15)	Age	6

Table E. Characteristics and quality assessment of studies included in the meta-regression analysis for the association between physical activity and ischemic stroke

Author (Year)	Study Name	Country	Time Period	Age Range	Sex	Sample Size	Activity Type	Effect Type	Effect	Covariates adjusted	NOS score
Abbott (1994)	Honolulu Heart Program	United States	1965-1990	55-68	Male	1854	Total daily activity	RR	Inactive: 1.8 (1.1, 3.1); Partially Active: 1.7 (1.0, 2.8); Active: 1.0	Systolic BP, Serum Cholesterol, Alcohol, Serum Glucose, Serum Uric Acid, Hematocrit	7
Agnarsson (1999)	The Reykjavik Study	Iceland	1979-1993. Mean follow up 10.6 years	45-80	Male	4484	Recreation	RR	Physical Activity after age 40: No: 1.00; Yes: 0.62 (0.40, 0.97)	Age, BMI, Smoking, Hypertension, FEV	7
Armstrong (2015)	The Million Women Study	United Kingdom	1998-Mean follow up 9 years	50-64	Female	1014862	Recreation	RR	Rarely/never: 1 (0.95, 1.06); At most once per week: 0.89 (0.84, 0.94); 2-3 times per week: 0.82 (0.77, 0.87); 4-6 times per week: 0.83 (0.76, 0.92); Daily: 0.91 (0.86, 0.97)	Adjusted For BMI-by-Age, Smoking-by-Age, Alcohol-by-Age, Stratified By SES and Region	7
Autenrieth (2013)	The ARIC Study	United States	1987-2007. Median follow up 18.8 years	45-64	Both	13069	Recreation	HR	Poor Physical Activity: 1.00; Intermediate Physical Activity: 0.93 (0.76, 1.14); Ideal Physical Activity: 0.84 (0.65, 1.07)	Age, Sex, Race-Field Center, Cigarette-Years, Educational Level, Waist-To-Hip Ratio, Systolic Blood Pressure, Antihypertensive Medication Use, Diabetes, Left Ventricular Hypertrophy, High-Density Lipoprotein Cholesterol, Low-Density Lipoprotein	8

Author (Year)	Study Name	Country	Time Period	Age Range	Sex	Sample Size	Activity Type	Effect Type	Effect	Covariates adjusted	NOS score
										Cholesterol, Lipoprotein(A), Fibrinogen, Von Willebrand Factor, White Blood Cell Count	
Bijnen (1998)	The Zutphen Study	Netherlands	1985-1995	64-84	Male	802	Recreation+ Commuting	RR	Lowest Tertile: 1.00; Middle Tertile: 0.65 (0.33, 1.25); Highest Tertile: 0.55 (0.24, 1.26)	Age, Baseline Stroke, Smoking, Alcohol	5
Calling (2006)	The Malmo Diet and Cancer Study	Sweden	1991-2003. Mean follow up 7.6 years.	45-73	Both	26942	Recreation	RR	Low Active: 1.00;<br>Active: 0.60 (0.50, 0.71)	Age, Sex	5
Chiuve (2008)	Health Professionals Follow-up Study and Nurses' Health Study	United States	1984-2004	38-53 (female)<br><br>40-75 (males)	Female<br><br>Male	71243 (females)<br><br>43685 (males)	Recreation	RR	Females:<br>0 hr/week: 1.66 (1.26, 2.20); 0.01-1.0 hr/week: 1.29 (0.97, 1.71); 1.0-3.5 hr/week: 1.19 (0.89, 1.58); 3.5-6.0 hr/week: 0.91 (0.65, 1.28); 6.0+ hr/week: 1.00<br><br>Males:<br>0 hr/week: 1.76 (1.34, 2.30); 0.01-1.0 hr/week: 1.41	Age, Calendar Year, Parental History of MI before 60, Regular Aspirin Use and Vitamin E Supplementation, Use of Hormone Therapy in Women	6

Author (Year)	Study Name	Country	Time Period	Age Range	Sex	Sample Size	Activity Type	Effect Type	Effect	Covariates adjusted	NOS score
									(1.00, 2.00); 1.0-3.5 hr/week: 1.34 (1.00, 1.81); 3.5-6.0 hr/week: 1.44 (1.03, 2.03); 6.0+ hr/week: 1.00		
Ellekjaer (2000)	A general health survey in Nord-Trondelag county in Norway	Norway	1984-1994	50-101	Female	13669	Recreation	RR	Low: 1.00; Medium: 0.75 (0.58, 0.97); High: 0.54 (0.39, 0.75)	(Participants Who Survived First 2 Years Of Follow-Up) Age, Smoking, Diabetes, BMI, Antihypertensive Medication, Systolic BP, Angina Pectoris, MI, Illness that Impairs Function in Daily Life, Education	7
Gulsvik (2012)	The Bergen Clinical Blood Pressure Survey	Norway	1965-2007.	22-75	Both	5653	recreation and transportation	HR	No/Low: 1.00; Moderate: 0.83 (0.67, 1.03); High: 0.66 (0.47, 0.93)	Age, Sex	6
Haheim (1993)	Men participated in a screening study in Oslo who later participated in the dietary and smoking intervention trial and the hypertension trial	Norway	1972-1984. Mean follow-up 6 years.	40-49	Male	14403	Recreation	RR	Risk of Stroke Incidence: Sedentary: 1.00; Moderate: 0.64 (0.38, 1.08); Intermediate+Great: 0.36 (0.15, 0.80) Risk of Stroke Mortality: Sedentary: 1.00; Moderate: 0.82		5

Author (Year)	Study Name	Country	Time Period	Age Range	Sex	Sample Size	Activity Type	Effect Type	Effect	Covariates adjusted	NOS score
									(0.33, 2.35); Intermediate+Great: 0.29 (0.03, 1.51)		
Hu (2000)	Nurses' Health Study	United States	1986-1994	40-65	Female	72488	Total Physical Activity	RR	0-2.0 MET hr/week: 1.00; 2.1-4.6 MET hr/week: 0.87 (0.62, 1.23); 4.7-10.4 MET hr/week: 0.83 (0.58, 1.19); 10.5-21.7 MET hr/week: 0.76 (0.52, 1.11); >21.7 MET hr/week: 0.52 (0.33, 0.80)	Age, Time, Cigarette Smoking, BMI, Menopausal Status, Hormone Replacement Therapy, Parental History MI Before Age 60, Alcohol Consumption, Aspirin Use, Hypertension, Diabetes, Hypercholesterolemia	7
Hu (2005)	Six independent population surveys in 5 geographic areas of Finland	Finland	1972-2003. Mean follow up 15.5 years.	25-64	Both	47721	Recreation	HR	Low: 1.00; Moderate: 0.85 (0.79, 0.92); High: 0.73 (0.64, 0.84)	Age, Area, Study Year, BMI, SBP, Cholesterol, Education, Smoking, Alcohol Consumption, Diabetes	7
Lapidus (1986)	A population study of women in Gothenburg, Sweden	Sweden	1968-1981. Mean follow-up 6.5 years.	38-60	Female	1462	Recreation	RR	Group I (Low): 10.1 (3.80, 27.1); Groups II, III, IV (More Active): 1.00	Age	6
Lee (1998)	Harvard University alumni	United States	1977-1990	43-88	Male	11130	Recreation+Commuting	RR	<1000 kcal/week: 1.00; 1000-1999 kcal/week: 0.76 (0.59, 0.98);	Age, Smoking, Alcohol, Early Parental Death	4

Author (Year)	Study Name	Country	Time Period	Age Range	Sex	Sample Size	Activity Type	Effect Type	Effect	Covariates adjusted	NOS score
									2000-2999 kcal/week: 0.54 (0.38, 0.76); 3000-3999 kcal/week: 0.78 (0.53, 1.15); ≥4000 kcal/week: 0.82 (0.58, 1.14)		
Lee (1999)	Physicians' Health Study	United States	1982-1995. Mean follow up 11.1 years.	40-84	Male	21823	Recreation	RR	Frequency of Vigorous Exercise: <1 Time/Week: 1.00; 1 Time/Week: 0.90 (0.66, 1.22); 2-4 Times/Week: 0.95 (0.74, 1.22); ≥5 Times/Week: 0.97 (0.71, 1.32)	Age, Treatment Assigned, Smoking, Alcohol, History Of Angina, Parental History of MI at <60 Years, BMI, History of Hypertension, History of High Cholesterol, History of Diabetes Mellitus	7
Lindenstrom (1993)	The Copenhagen City Health Study (CCHS)	Denmark	1976-1988	35+	Female	7060	Recreation	RR	Inactive: 1.45 (1.01, 2.08); Active: 1.00	Age, Education, Household Income, Smoking, Daily Number Cigarettes, Alcohol, Daily Consumption of Tranquilizers, BMI	7
Myint (2006)	European Prospective Investigation into Cancer-Norfolk (EPIC-Norfolk) population	United Kingdom	1993-2004. Mean follow up 8.6 years.	40-79	Both	22602	Recreation+Occupational	RR	Inactive: 1.00; Moderately Inactive: 0.78 (0.61, 1.00); Moderately Active: 0.66 (0.49, 0.91); Active: 0.70	Age, Sex, Systolic Blood Pressure, BMI, Cholesterol, History of Diabetes and Cigarette Smoking Status	7

Author (Year)	Study Name	Country	Time Period	Age Range	Sex	Sample Size	Activity Type	Effect Type	Effect	Covariates adjusted	NOS score
	study								(0.49, 0.99)		
Okada (1976)	Men and women from Akabane and Asahi in Japan	Japan	1964-1970	40-79	Both	4186	Occupational	RR	None: 1.20 (0.13, 4.69); Casual: 2.30 (0.41, 6.95); Restrained: 2.30 (0.13, 4.69); Regular: 1.00	Age, Sex	5
Paffenbarger (1978)	San Francisco longshoremen	United States	1951-1972	35-74	Male	3686	Occupational	RR	4750-8250 Kcal/Week: 1.50 (0.56, 4.49); 8500-10750 Kcal/Week: 1.00	Age, Smoking, Systolic BP	5
Paganini-Hill (2001)	Residents of Leisure World Laguna Hills	United States	1981-1998	44-101	Male<br><br>Female	4722 (males)<br><br>8532 (females)	Recreation	RR	Males: <0.5 hr/day: 1.00; 1+ hr/day: 0.85 (0.72, 1.01)<br><br>Females: <0.5 hr/day: 1.00; 1+ hr/day: 0.83 (0.73, 0.95)	Age	5
Salonen (1982)	Random population sample from two counties of Eastern Finland	Finland	1972-1978. Mean follow-up 3 years.	30-59 (males)<br><br>35-59 (females)	Male<br><br>Female	3978 (males)<br><br>3688 (females)	Recreation	RR	Males: Low Activity in Leisure: 1.00 (0.70, 1.50); High Activity in Leisure: 1.00<br><br>Females: Low Activity in Leisure: 1.30 (0.80, 2.00); High Activity in Leisure: 1.00	Age, Serum Cholesterol, Diastolic BP, BMI, Daily Number of Tobacco Products	7

Author (Year)	Study Name	Country	Time Period	Age Range	Sex	Sample Size	Activity Type	Effect Type	Effect	Covariates adjusted	NOS score
Sattelmair (2010)	Women's Health Study	United States	1992-2004. Mean follow up 10.4 years.	45+	Female	39315	Recreation	RR	0 – 199 kcal/week: 1.00; 200 – 599 kcal/week: 1.11 (0.85, 1.46); 600 – 1499 kcal/week: 0.87 (0.66, 1.16); 1500 + kcal/week: 0.88 (0.65, 1.19)	Age, Randomized Treatment Assignment, Smoking; Alcohol; Saturated fat, Fruit and Vegetable, and Fiber intake; Postmenopausal hormone therapy; Menopausal status, Parental history of myocardial infarction, Migraine Aura, BMI, History of Diabetes, History of Elevated Cholesterol, and History of Hypertension.	7
Simonsick (1993)	Established Populations for Epidemiologic Studies of the Elderly (EPESE)	United States	1982-1989	65+	Both	2712 (East boston)<br><br>2190 (New Haven)<br><br>2539 (Iowa)	Recreation+Housework	OR	East Boston: Inactive: 1.00; Moderate: 1.73 (0.98, 3.06); High: 1.21 (0.56, 2.61)<br><br>New Haven: Inactive: 1.00; Moderate: 1.29 (0.72, 2.32); High: 1.05 (0.52, 2.12)<br><br>Iowa: Inactive: 1.00; Moderate: 0.97 (0.64, 1.48); High: 0.56 (0.31, 1.03)	Age, Sex, Education, Work Status, Smoking, Depressive Symptomatology, Self-Rated Health, Respiratory Symptoms, History of MI and Stroke, Diabetes at Baseline, Angina at Baseline	5

Author (Year)	Study Name	Country	Time Period	Age Range	Sex	Sample Size	Activity Type	Effect Type	Effect	Covariates adjusted	NOS score
Wannamethee (1992)	The British regional heart study	United Kingdom	1978-1988	40-59	Male	7735	Recreation+Commuting	RR	Inactive: 1.00 Occasional: 0.80 (0.34, 1.81); Light: 0.60 (0.23, 1.45) Moderate: 0.70 (0.19, 1.46) Moderately Vigorous: 0.70 (0.12, 1.16) Vigorous: 0.20 (0.00, 0.82)	Age, Social Class, Smoking, Heavy Drinking, BMI, Systolic BP	7
Willey (2009)	The Northern Manhattan Study	United States	1993-2002	40-107	Both	3298	Recreation	HR	No Physical Activity: 1.00; Light: 0.94 (0.71, 1.25); Moderate To Heavy: 0.65 (0.43, 0.98)	Hypertension, Diabetes, Moderate Alcohol Intake and Tobacco Use	8
Zhang (2013)	The Kailuan study	China	2006-2010	18-98	Both	91698	Recreation	HR	Non-ideal: 1.00; Ideal: 0.76 (0.64, 0.90)	Age, Sex, Hospital, Education, Income, Diet, Total Cholesterol, Blood Pressure, Fasting Blood Glucose, BMI	6

References

Abbott RD, Rodriguez BL, Burchfiel CM, Curb JD. Physical activity in older middle-aged men and reduced risk of stroke: the Honolulu Heart Program. Am J Epidemiol. 1994; 139(9): 881-93.

Agnarsson U, Thorgeirsson G, Sigvaldason H, Sigfusson N. Effects of leisure-time physical activity and ventilatory function on risk for stroke in men: the Reykjavík Study. Ann Intern Med. 1999; 130(12): 987-90.

Akesson A, Weismayer C, Newby PK, Wolk A. Combined effect of low-risk dietary and lifestyle behaviors in primary prevention of myocardial infarction in women. Arch Intern Med. 2007; 167(19): 2122-7.

Allesøe K, Holtermann A, Aadahl M, Thomsen JF, Hundrup YA, Søgaard K. High occupational physical activity and risk of ischaemic heart disease in women: The interplay with physical activity during leisure time. European journal of preventive cardiology. 2015 ;22(12):1601-8.

Armstrong ME, Green J, Reeves GK, Beral V, Cairns BJ. Frequent physical activity may not reduce vascular disease risk as much as moderate activity: large prospective study of UK women. Circulation. 2015 Feb 16:CIRCULATIONAHA-114.

Autenrieth CS, Evenson KR, Yatsuya H, Shahar E, Baggett C, Rosamond WD. Association between physical activity and risk of stroke subtypes: the atherosclerosis risk in communities study. Neuroepidemiology. 2013; 40(2): 109-116.

Baan CA, Stolk RP, Grobbee DE, Witteman JC, Feskens EJ. Physical activity in elderly subjects with impaired glucose tolerance and newly diagnosed diabetes mellitus. Am J Epidemiol. 1999; 149(3): 219-27.

Bardia A, Hartmann LC, Vachon CM, Vierkant RA, Wang AH, Olson JE, Sellers TA, Cerhan JR. Recreational physical activity and risk of postmenopausal breast cancer based on hormone receptor status. Arch Intern Med. 2006; 166(22): 2478-83.

Batty GD, Shipley MJ, Marmot M, Smith GD. Physical activity and cause-specific mortality in men with Type 2 diabetes/impaired glucose tolerance: evidence from the Whitehall study. Diabet Med. 2002; 19(7): 580-8.

Bijnen FC, Caspersen CJ, Feskens EJ, Saris WH, Mosterd WL, Kromhout D. Physical activity and 10-year mortality from cardiovascular diseases and all causes: The Zutphen Elderly Study. Arch Intern Med. 1998; 158(14): 1499-505.

Bonora E, Kiechl S, Willeit J, Oberhollenzer F, Egger G, Meigs JB, Bonadonna RC, Muggeo M. Population-based incidence rates and risk factors for type 2 diabetes in white individuals: the Bruneck study. Diabetes. 2004;53(7):1782–9.

Borch KB, Lund E, Braaten T, Weiderpass E. Physical activity and the risk of postmenopausal breast cancer - the Norwegian Women and Cancer Study. J Negat Results Biomed. 2014; 13: 3.

Bostick RM, Potter JD, Kushi LH, Sellers TA, Steinmetz KA, McKenzie DR, Gapstur SM, Folsom AR. Sugar, meat, and fat intake, and non-dietary risk factors for colon cancer incidence in Iowa women (United States). Cancer Causes Control. 1994; 5(1): 38-52.

Breslow RA, Ballard-Barbash R, Munoz K, Graubard BI. Long-term recreational physical activity and breast cancer in the National Health and Nutrition Examination Survey I epidemiologic follow-up study. Cancer Epidemiol Biomarkers Prev. 2001; 10(7): 805-8.

Burchfiel CM, Sharp DS, Curb JD, Rodriguez BL, Hwang LJ, Marcus EB, Yano K. Physical activity and incidence of diabetes: the Honolulu Heart Program. Am J Epidemiol. 1995;141(4): 360–8.

Calling S, Hedblad B, Engström G, Berglund G, Janzon L. Effects of body fatness and physical activity on cardiovascular risk: risk prediction using the bioelectrical impedance method. Scandinavian journal of public Health. 2006 Dec 1;34(6):568-75.

Calton BA, Lacey JV Jr, Schatzkin A, Schairer C, Colbert LH, Albanes D, Leitzmann MF. Physical activity and the risk of colon cancer among women: a prospective cohort study (United States). Int J Cancer. 2006; 119(2): 385-91.

Carlsson S, Ahlbom A, Lichtenstein P, Andersson T. Shared genetic influence of BMI, physical activity and type 2 diabetes: a twin study. Diabetologia. 2013; 56(5): 1031-5.

Carlsson S, Midthjell K, Tesfamarian MY, Grill V. Age, overweight and physical inactivity increase the risk of latent autoimmune diabetes in adults: results from the Nord-Trondelag health study. Diabetologia. 2007;50(1):55–8.

Cerhan JR, Chiu BC, Wallace RB, Lemke JH, Lynch CF, Torner JC, Rubenstein LM. Physical activity, physical function, and the risk of breast cancer in a prospective study among elderly women. J Gerontol A Biol Sci Med Sci. 1998; 53(4): M251-256.

Chang S-C, Ziegler RG, Dunn B, Stolzenberg-Solomon R, Lacey JV Jr, Huang W-Y, Schatzkin A, Reding D, Hoover RN, Hartge P, Leitzmann MF. Association of energy intake and energy balance with postmenopausal breast cancer in the prostate, lung, colorectal, and ovarian cancer screening trial. Cancer Epidemiol Biomarkers Prev. 2006; 15(2): 334-41.

Chao A, Connell CJ, Jacobs EJ, McCullough ML, Patel AV, Calle EE, Cokkinides VE, Thun MJ. Amount, type, and timing of recreational physical activity in relation to colon and rectal cancer in older adults: the Cancer Prevention Study II Nutrition Cohort. Cancer Epidemiol Biomarkers Prev. 2004; 13(12): 2187-95.

Chen J, Millar WJ. Health effects of physical activity. Health Rep. 1999; 11(1): 21-31.

Chien KL, Chen MF, Hsu HC, Su TC, Lee YT. Sports activity and risk of type 2 diabetes in Chinese. Diabetes Res Clin Pract. 2009;84(3):311–8.

Chiuve SE, McCullough ML, Sacks FM, Rimm EB. Healthy lifestyle factors in the primary prevention of coronary heart disease among men: benefits among users and nonusers of lipid-lowering and antihypertensive medications. Circulation. 2006; 114(2): 160-7.

Chiuve SE, Rexrode KM, Spiegelman D, Logroscino G, Manson JE, Rimm EB. Primary prevention of stroke by healthy lifestyle. Circulation. 2008; 118(9): 947-954.

Chomistek AK, Manson JE, Stefanick ML, Lu B, Sands-Lincoln M, Going SB, Garcia L, Allison MA, Sims ST, LaMonte MJ, Johnson KC, Eaton CB. Relationship of sedentary behavior and physical activity to incident cardiovascular disease: results from the Womenâ€™s Health Initiative. J Am Coll Cardiol. 2013; 61(23): 2346-54.

Colbert LH, Hartman TJ, Malila N, Limburg PJ, Pietinen P, Virtamo J, Taylor PR, Albanes D. Physical activity in relation to cancer of the colon and rectum in a cohort of male smokers. Cancer Epidemiol Biomarkers Prev. 2001; 10(3): 265-8.

Colditz GA, Feskanich D, Chen WY, Hunter DJ, Willett WC. Physical activity and risk of breast cancer in premenopausal women. Br J Cancer. 2003; 89(5): 847-51.

Dallal CM, Sullivan-Halley J, Ross RK, Wang Y, Deapen D, Horn-Ross PL, Reynolds P, Stram DO, Clarke CA, Anton-Culver H, Ziogas A, Peel D, West DW, Wright W, Bernstein L. Long-term recreational physical activity and risk of invasive and in situ breast cancer: the California teachers study. Arch Intern Med. 2007; 167(4): 408-15.

Demakakos P, Hamer M, Stamatakis E, Steptoe A. Low-intensity physical activity is associated with reduced risk of incident type 2 diabetes in older adults: evidence from the English Longitudinal Study of Ageing. Diabetologia. 2010; 53(9): 1877-1885.

Donahue, R. P., Abbott, R. D., Reed, D. M., & Yano, K. (1988). Physical activity and coronary heart disease in middle-aged and elderly men: the Honolulu Heart Program. American Journal of Public Health, 78(6), 683-685.

Doi Y, Ninomiya T, Hata J, Hirakawa Y, Mukai N, Iwase M, Kiyohara Y. Two risk score models for predicting incident type 2 diabetes in Japan. Diabet Med. 2012;29(1):107–14.

Dorgan JF, Brown C, Barrett M, Splansky GL, Kreger BE, D'Agostino RB, Albanes D, Schatzkin A. Physical activity and risk of breast cancer in the Framingham Heart Study. Am J Epidemiol. 1994; 139(7): 662-9.

Dotevall A, Johansson S, Wilhelmsen L, Rosengren A. Increased levels of triglycerides, BMI and blood pressure and low physical activity increase the risk of diabetes in Swedish women. A prospective 18-year follow-up of the BEDA study. Diabet Med. 2004;21(6):615–22.

Eaton CB, Medalie JH, Flocke SA, Zyzanski SJ, Yaari S, Goldbourt U. Self-reported physical activity predicts long-term coronary heart disease and all-cause mortalities. Twenty-one-year follow-up of the Israeli Ischemic Heart Disease Study. Arch Fam Med. 1995; 4(4): 323-9.

Eliassen AH, Hankinson SE, Rosner B, Holmes MD, Willett WC. Physical activity and risk of breast cancer among postmenopausal women. Arch Intern Med. 2010; 170(19): 1758-64.

Ellekjaer H, Holmen J, Ellekjaer E, Vatten L. Physical activity and stroke mortality in women. Ten-year follow-up of the Nord-Trondelag health survey, 1984-1986. Stroke. 2000; 31(1): 14-8.

Elwood P, Galante J, Pickering J, Palmer S, Bayer A, Ben-Shlomo Y, Longley M, Gallacher J. Healthy lifestyles reduce the incidence of chronic diseases and dementia: evidence from the caerphilly cohort study. PLoS ONE. 2013;8(12):e81877.

Fan S, Chen J, Huang J, Li Y, Zhao L, Liu X, Li J, Cao J, Yu L, Deng Y, Chen N, Guo D, et al. Physical activity level and incident type 2 diabetes among Chinese adults. Med Sci Sports Exerc. 2015;47(4):751–6.

Folsom AR, Arnett DK, Hutchinson RG, Liao F, Clegg LX, Cooper LS. Physical activity and incidence of coronary heart disease in middle-aged women and men. Med Sci Sports Exerc. 1997; 29(7): 901-9.

Folsom AR, Kushi LH, Hong CP. Physical activity and incident diabetes mellitus in postmenopausal women. Am J Public Health. 2000; 90(1): 134-8.

Fraser G, Pearce N. Occupational physical activity and risk of cancer of the colon and rectum in New Zealand males. Cancer Causes Control. 1993; 4(1): 45-50.

Fretts AM, Howard BV, Kriska AM, Smith NL, Lumley T, Lee ET, Russell M, Siscovick D. Physical activity and incident diabetes in American Indians: the Strong Heart Study. Am J Epidemiol. 2009;170(5):632–9.

Friedenreich C, Norat T, Steindorf K, Boutron-Ruault M-C, Pischon T, Mazuir M, Clavel-Chapelon F, Linseisen J, Boeing H, Bergman M, Johnsen NF, Tjønneland A, Overvad K, Mendez M, Quirós JR, Martinez C, Dorronsoro M, Navarro C, Gurrea AB, Bingham S, Khaw K-T, Allen N, Key T, Trichopoulou A, Trichopoulos D, Orfanou N, Krogh V, Palli D, Tumino R, Panico S, Vineis P, Bueno-de-Mesquita HB, Peeters PHM, Monninkhof E, Berglund G, Manjer J, Ferrari P, Slimani N, Kaaks R, Riboli E.

Physical activity and risk of colon and rectal cancers: the European prospective investigation into cancer and nutrition. Cancer Epidemiol Biomarkers Prev. 2006; 15(12): 2398-407.

Frisch RE, Wyshak G, Witschi J, Albright NL, Albright TE, Schiff I. Lower lifetime occurrence of breast cancer and cancers of the reproductive system among former college athletes. Int J Fertil. 1987; 32(3): 217-25.

Gerhardsson M, Norell SE, Kiviranta H, Pedersen NL, Ahlbom A. Sedentary jobs and colon cancer. Am J Epidemiol. 1986; 123(5): 775-80.

Giovannucci E, Ascherio A, Rimm EB, Colditz GA, Stampfer MJ, Willett WC. Physical activity, obesity, and risk for colon cancer and adenoma in men. Ann Intern Med. 1995; 122(5): 327-34.

Grontved A, Pan A, Mekary RA, Stampfer M, Willett WC, Manson JE, Hu FB. Muscle-strengthening and conditioning activities and risk of type 2 diabetes: a prospective study in two cohorts of US women. PLoS Med. 2014;11(1):e1001587.

Grontved A, Rimm EB, Willett WC, Andersen LB, Hu FB. A prospective study of weight training and risk of type 2 diabetes mellitus in men. Arch Intern Med. 2012;172(17):1306–12.

Gulsvik AK, Thelle DS, Samuelsen SO, Myrstad M, Mowé M, Wyller TB. Ageing, physical activity and mortality—a 42-year follow-up study. International journal of epidemiology. 2012 Apr 1;41(2):521-30.

Gurwitz JH, Field TS, Glynn RJ, Manson JE, Avorn J, Taylor JO, Hennekens CH. Risk factors for non-insulin-dependent diabetes mellitus requiring treatment in the elderly. J Am Geriatr Soc. 1994; 42(12): 1235-40.

Haapanen N, Miilunpalo S, Vuori I, Oja P, Pasanen M. Association of leisure time physical activity with the risk of coronary heart disease, hypertension and diabetes in middle-aged men and women. Int J Epidemiol. 1997; 26(4): 739-47.

Harari G, Green MS, Zelber-Sagi S. Combined association of occupational and leisure-time physical activity with all-cause and coronary heart disease mortality among a cohort of men followed-up for 22 years. Occupational and environmental medicine. 2015 Mar 24:oemed-2014.

Hastert TA, Beresford SA, Patterson RE, Kristal AR, White E. Adherence to WCRF/AICR cancer prevention recommendations and risk of post-menopausal breast cancer. Cancer Epidemiology Biomarkers & Prevention. 2013 Jun 18:cebp-0210.

Helmrich SP, Ragland DR, Paffenbarger RS Jr. Prevention of non-insulin-dependent diabetes mellitus with physical activity. Med Sci Sports Exerc. 1994; 26(7): 824-30.

Hildebrand JS, Gapstur SM, Campbell PT, Gaudet MM, Patel AV. Recreational physical activity and leisure-time sitting in relation to postmenopausal breast cancer risk. Cancer Epidemiol Biomarkers Prev. 2013; 22(10): 1906-1912.

Hillsdon M, Thorogood M, Murphy M, Jones L. Can a simple measure of vigorous physical activity predict future mortality? Results from the OXCHECK study. Public Health Nutr. 2004; 7(4): 557-62.

Holme I, Tonstad S, Sogaard AJ, Larsen PG, Haheim LL. Leisure time physical activity in middle age predicts the metabolic syndrome in old age: results of a 28-year follow-up of men in the Oslo study. BMC Public Health. 2007;7:154.

Holtermann A, Mortensen OS, Søgaard K, Gyntelberg F, Suadicani P. Risk factors for ischaemic heart disease mortality among men with different occupational physical demands. A 30-year prospective cohort study. BMJ open. 2012 Jan 1;2(1):e000279.

Howard RA, Freedman DM, Park Y, Hollenbeck A, Schatzkin A, Leitzmann MF. Physical activity, sedentary behavior, and the risk of colon and rectal cancer in the NIH-AARP Diet and Health Study. Cancer Causes Control. 2008; 19(9): 939-53.

Howard RA, Leitzmann MF, Linet MS, Freedman DM. Physical activity and breast cancer risk among pre- and postmenopausal women in the U.S. Radiologic Technologists cohort. Cancer Causes Control. 2009; 20(3): 323-33.

Hsia J, Wu L, Allen C, Oberman A, Lawson WE, TorrÃ©ns J, Safford M, Limacher MC, Howard BV, Women's Health Initiative Research Group. Physical activity and diabetes risk in postmenopausal women. Am J Prev Med. 2005; 28(1): 19-25.

Hu FB, Leitzmann MF, Stampfer MJ, Colditz GA, Willett WC, Rimm EB. Physical activity and television watching in relation to risk for type 2 diabetes mellitus in men. Arch Intern Med. 2001; 161(12): 1542-8.

Hu FB, Sigal RJ, Rich-Edwards JW, Colditz GA, Solomon CG, Willett WC, Speizer FE, Manson JE. Walking compared with vigorous physical activity and risk of type 2 diabetes in women: a prospective study. JAMA. 1999; 282(15): 1433-9.

Hu FB, Stampfer MJ, Colditz GA, Ascherio A, Rexrode KM, Willett WC, Manson JE. Physical activity and risk of stroke in women. JAMA. 2000; 283(22): 2961-7.

Hu G, Jousilahti P, Borodulin K, Barengo NC, Lakka TA, Nissinen A, Tuomilehto J. Occupational, commuting and leisure-time physical activity in relation to coronary heart disease among middle-aged Finnish men and women. Atherosclerosis. 2007; 194(2): 490-7.

Hu G, Qiao Q, Silventoinen K, Eriksson JG, Jousilahti P, Lindström J, Valle TT, Nissinen A, Tuomilehto J. Occupational, commuting, and leisure-time physical activity in relation to risk for Type 2 diabetes in middle-aged Finnish men and women. Diabetologia. 2003; 46(3): 322-9.

Hu G, Sarti C, Jousilahti P, Silventoinen K, Barengo NC, Tuomilehto J. Leisure time, occupational, and commuting physical activity and the risk of stroke. Stroke. 2005; 36(9): 1994-9.

Håheim LL, Holme I, Hjermann I, Leren P. Risk factors of stroke incidence and mortality. A 12-year follow-up of the Oslo Study. Stroke. 1993; 24(10): 1484-9.

Inoue M, Iso H, Yamamoto S, Kurahashi N, Iwasaki M, Sasazuki S, Tsugane S, Japan Public Health Center-Based Prospective Study Group. Daily total physical activity level and premature death in men and women: results from a large-scale population-based cohort study in Japan (JPHC study). Ann Epidemiol. 2008; 18(7): 522-30.

James SA, Jamjoum L, Raghunathan TE, Strogatz DS, Furth ED, Khazanie PG. Physical activity and NIDDM in African-Americans. The Pitt County Study. Diabetes Care. 1998; 21(4): 555-62.

Jefferis BJ, Whincup PH, Lennon LT, Papacosta O, Goya Wannamethee S. Physical Activity in Older Men: Longitudinal Associations with Inflammatory and Hemostatic Biomarkers, N-Terminal Pro-Brain Natriuretic Peptide, and Onset of Coronary Heart Disease and Mortality. Journal of the American Geriatrics Society. 2014 Apr 1;62(4):599-606.

Jefferis BJ, Whincup PH, Lennon L, Wannamethee SG. Longitudinal associations between changes in physical activity and onset of type 2 diabetes in older British men: the influence of adiposity. Diabetes Care. 2012;35(9):1876–83.

Joseph J, Svartberg J, Njolstad I, Schirmer H. Incidence of and risk factors for type-2 diabetes in a general population: the Tromso Study. Scand J Public Health. 2010;38(7):768–75.

Kaprio J, Kujala UM, Koskenvuo M, Sarna S. Physical activity and other risk factors in male twin-pairs discordant for coronary heart disease. Atherosclerosis. 2000; 150(1): 193-200.

Krishnan S, Rosenberg L, Palmer JR. Physical activity and television watching in relation to risk of type 2 diabetes: the Black Women's Health Study. Am J Epidemiol. 2009;169(4):428–34.

Koloverou E, Panagiotakos DB, Pitsavos C, Chrysohoou C, Georgousopoulou EN, Pitaraki E, Metaxa V, Stefanadis C. 10-year incidence of diabetes and associated risk factors in Greece: the ATTICA study (2002–2012). Rev Diabet Stud. 2014;11(2):181–9.

Laaksonen MA, Knekt P, Rissanen H, Harkanen T, Virtala E, Marniemi J, Aromaa A, Heliovaara M, Reunanen A. The relative importance of modifiable potential risk factors of type 2 diabetes: a meta-analysis of two cohorts. Eur J Epidemiol. 2010;25(2):115–24.

Lakka TA, Venäläinen JM, Rauramaa R, Salonen R, Tuomilehto J, Salonen JT. Relation of leisure-time physical activity and cardiorespiratory fitness to the risk of acute myocardial infarction. N Engl J Med. 1994; 330(22): 1549-54.

Lapidus L, Bengtsson C. Socioeconomic factors and physical activity in relation to cardiovascular disease and death. A 12 year follow up of participants in a population study of women in Gothenburg, Sweden. Br Heart J. 1986; 55(3): 295-301.

Larsson SC, Rutegård J, Bergkvist L, Wolk A. Physical activity, obesity, and risk of colon and rectal cancer in a cohort of Swedish men. Eur J Cancer. 2006; 42(15): 2590-7.

Lee D, Park I, Jun TW, Nam BH, Cho S, Blair SN, Kim YS. Physical activity and body mass index and their associations with the development of type 2 diabetes in korean men. Am J Epidemiol. 2012; 176(1): 43-51.

Lee IM, Hennekens CH, Berger K, Buring JE, Manson JE. Exercise and risk of stroke in male physicians. Stroke. 1999; 30(1): 1-6.

Lee IM, Manson JE, Ajani U, Paffenbarger RS Jr, Hennekens CH, Buring JE. Physical activity and risk of colon cancer: the Physicians' Health Study (United States). Cancer Causes Control. 1997; 8(4): 568-74.

Lee IM, Paffenbarger RS Jr. Physical activity and its relation to cancer risk: a prospective study of college alumni. Med Sci Sports Exerc. 1994; 26(7): 831-7.

Lee IM, Paffenbarger RS Jr. Physical activity and stroke incidence: the Harvard Alumni Health Study. Stroke. 1998; 29(10): 2049-54.

Lee IM, Rexrode KM, Cook NR, Hennekens CH, Burin JE. Physical activity and breast cancer risk: the Women's Health Study (United States). Cancer Causes Control. 2001; 12(2): 137-45.

Lee K-J, Inoue M, Otani T, Iwasaki M, Sasazuki S, Tsugane S, JPHC Study Group. Physical activity and risk of colorectal cancer in Japanese men and women: the Japan Public Health Center-based prospective study. Cancer Causes Control. 2007; 18(2): 199-209.

Leitzmann MF, Moore SC, Peters TM, Lacey JV Jr, Schatzkin A, Schairer C, Brinton LA, Albanes D. Prospective study of physical activity and risk of postmenopausal breast cancer. Breast Cancer Res. 2008; 10(5): R92.

Leon AS, Myers MJ, Connett J. Leisure time physical activity and the 16-year risks of mortality from coronary heart disease and all-causes in the Multiple Risk Factor Intervention Trial (MRFIT). Int J Sports Med. 1997; S208-215.

Li TY, Rana JS, Manson JE, Willett WC, Stampfer MJ, Colditz GA, Rexrode KM, Hu FB. Obesity as compared with physical activity in predicting risk of coronary heart disease in women. Circulation. 2006; 113(4): 499-506.

LindenstrÃ¸m E, Boysen G, Nyboe J. Lifestyle factors and risk of cerebrovascular disease in women. The Copenhagen City Heart Study. Stroke. 1993; 24(10): 1468-72.

Longo-Mbenza B, On'Kin JK, Okwe AN, Kabangu NK, Fuele SM. Metabolic syndrome, aging, physical inactivity, and incidence of type 2 diabetes in general African population. Diabetes and Vascular Disease Research. 2009 Oct 28.

Lucke J, Waters B, Hockey R, Spallek M, Gibson R, Byles J, Dobson A. Trends in women's risk factors and chronic conditions: findings from the Australian Longitudinal Study on Women's Health. Womens Health (Lond Engl). 2007;3(4):423–32.

Luoto R, Latikka P, Pukkala E, Hakulinen T, Vihko V. The effect of physical activity on breast cancer risk: a cohort study of 30,548 women. Eur J Epidemiol. 2000; 16(10): 973-80.

Magliano DJ, Barr EL, Zimmet PZ, Cameron AJ, Dunstan DW, Colagiuri S, Jolley D, Owen N, Phillips P, Tapp RJ, Welborn TA, Shaw JE. Glucose indices, health behaviors, and incidence of diabetes in Australia: the Australian diabetes, obesity and lifestyle study. Diabetes Care. 2008;31(2):267–72.

Mai PL, Sullivan-Halley J, Ursin G, Stram DO, Deapen D, Villaluna D, Horn-Ross PL, Clarke CA, Reynolds P, Ross RK, West DW, Anton-Culver H, Ziogas A, Bernstein L. Physical activity and colon cancer risk among women in the California Teachers Study. Cancer Epidemiol Biomarkers Prev. 2007; 16(3): 517-25.

Mannsverk J, Wilsgaard T, Mathiesen EB, Løchen ML, Rasmussen K, Thelle DS, Njølstad I, Hopstock LA, Bønaa KH. Trends in Modifiable Risk Factors are Associated With Declining Incidence of Hospitalized and Non-Hospitalized Acute Coronary Heart Disease in a Population. Circulation. 2015 Nov 18:CIRCULATIONAHA-115.

Manson JE, Nathan DM, Krolewski AS, Stampfer MJ, Willett WC, Hennekens CH. A prospective study of exercise and incidence of diabetes among US male physicians. JAMA. 1992; 268(1): 63-7.

Manson JE, Rimm EB, Stampfer MJ, Colditz GA, Willett WC, Krolewski AS, Rosner B, Hennekens CH, Speizer FE. Physical activity and incidence of non-insulin-dependent diabetes mellitus in women. Lancet. 1991; 338(8770): 774-8.

Margolis KL, Mucci L, Braaten T, Kumle M, Trolle Lagerros Y, Adami H-O, Lund E, Weiderpass E. Physical activity in different periods of life and the risk of breast cancer: the Norwegian-Swedish Women's Lifestyle and Health cohort study. Cancer Epidemiol Biomarkers Prev. 2005; 14(1): 27-32.

Maruti SS, Willett WC, Feskanich D, Rosner B, Colditz GA. A prospective study of age-specific physical activity and premenopausal breast cancer. J Natl Cancer Inst. 2008; 100(10): 728-37.

McTiernan A, Kooperberg C, White E, Wilcox S, Coates R, Adams-Campbell LL, Woods N, Ockene J, Women's Health Initiative Cohort Study. Recreational physical activity and the risk of breast cancer in postmenopausal women: the Women's Health Initiative Cohort Study. JAMA. 2003; 290(10): 1331-6.

Meisinger C, Löwel H, Heier M, Kandler U, Döring A. Association of sports activities in leisure time and incident myocardial infarction in middle-aged men and women from the general population: the MONICA/KORA Augsburg cohort study. Eur J Cardiovasc Prev Rehabil. 2007; 14(6): 788-92.

Meisinger C, Löwel H, Thorand B, Döring A. Leisure time physical activity and the risk of type 2 diabetes in men and women from the general population. Diabetologia. 2005; 48(1): 27-34.

Menotti A, Puddu PE, Maiani G, Catasta G. Lifestyle behaviour and lifetime incidence of heart diseases. International journal of cardiology. 2015 Dec 15;201:293-9.

Mertens AJ, Sweeney C, Shahar E, Rosamond WD, Folsom AR. Physical activity and breast cancer incidence in middle-aged women: a prospective cohort study. Breast Cancer Res Treat. 2006; 97(2): 209-14.

Moradi T, Adami H-O, Ekbom A, WedrÃ©n S, Terry P, Floderus B, Lichtenstein P. Physical activity and risk for breast cancer a prospective cohort study among Swedish twins. Int J Cancer. 2002; 100(1): 76-81.

Moradi T, Adami HO, Bergström R, Gridley G, Wolk A, Gerhardsson M, Dosemeci M, Nyrén O. Occupational physical activity and risk for breast cancer in a nationwide cohort study in Sweden. Cancer Causes Control. 1999; 10(5): 423-30.

Moradi T, Gridley G, Björk J, Dosemeci M, Ji B-T, Berkel HJ, Lemeshow S. Occupational physical activity and risk for cancer of the colon and rectum in Sweden among men and women by anatomic subsite. Eur J Cancer Prev. 2008; 17(3): 201-8.

Morris JN, Clayton DG, Everitt MG, Semmence AM, Burgess EH. Exercise in leisure time: coronary attack and death rates. Br Heart J. 1990; 63(6): 325-34.

Mozaffarian D, Kamineni A, Carnethon M, DjoussÃ© L, Mukamal KJ, Siscovick D. Lifestyle risk factors and new-onset diabetes mellitus in older adults: the cardiovascular health study. Arch Intern Med. 2009; 169(8): 798-807.

Myint PK, Luben RN, Wareham NJ, Welch AA, Bingham SA, Day NE, Khaw K-T. Combined work and leisure physical activity and risk of stroke in men and women in the European prospective investigation into Cancer-Norfolk Prospective Population Study. Neuroepidemiology. 2006; 27(3): 122-129.

Nilsen TIL, Romundstad PR, Petersen H, Gunnell D, Vatten LJ. Recreational physical activity and cancer risk in subsites of the colon (the Nord-Trøndelag Health Study). Cancer Epidemiol Biomarkers Prev. 2008; 17(1): 183-8.

Okada H, Horibe H, Yoshiyuki O, Hayakawa N, Aoki N. A prospective study of cerebrovascular disease in Japanese rural communities, Akabane and Asahi. Part 1: evaluation of risk factors in the occurrence of cerebral hemorrhage and thrombosis. Stroke. 1976; 7(6): 599-607.

Okada K, Hayashi T, Tsumura K, Suematsu C, Endo G, Fujii S. Leisure-time physical activity at weekends and the risk of Type 2 diabetes mellitus in Japanese men: the Osaka Health Survey. Diabet Med. 2000; 17(1): 53-8.

Paffenbarger RS Jr, Brand RJ, Sholtz RI, Jung DL. Energy expenditure, cigarette smoking, and blood pressure level as related to death from specific diseases. Am J Epidemiol. 1978; 108(1): 12-8.

Paffenbarger RS Jr, Wing AL, Hyde RT. Physical activity as an index of heart attack risk in college alumni. Am J Epidemiol. 1978; 108(3): 161-75.

Paganini-Hill A, Perez Barreto M. Stroke risk in older men and women: aspirin, estrogen, exercise, vitamins, and other factors. J Gend Specif Med. 2001; 4(2): 18-28.

Panagiotakos DB, Pitsavos C, Skoumas Y, Lentzas Y, Stefanadis C. Five-year incidence of type 2 diabetes mellitus among cardiovascular disease-free Greek adults: findings from the ATTICA study. Vasc Health Risk Manag. 2008; 4(3): 691-8.

Pedersen JØ, Heitmann BL, Schnohr P, Grønbaek M. The combined influence of leisure-time physical activity and weekly alcohol intake on fatal ischaemic heart disease and all-cause mortality. Eur Heart J. 2008; 29(2): 204-12.

Peters TM, Schatzkin A, Gierach GL, Moore SC, Lacey JV Jr, Wareham NJ, Ekelund U, Hollenbeck AR, Leitzmann MF. Physical activity and postmenopausal breast cancer risk in the NIH-AARP diet and health study. Cancer Epidemiol Biomarkers Prev. 2009; 18(1): 289-96.

Pronk A, Ji B-T, Shu X-O, Chow W-H, Xue S, Yang G, Li H-L, Rothman N, Gao Y-T, Zheng W, Matthews CE. Physical activity and breast cancer risk in Chinese women. Br J Cancer. 2011; 105(9): 1443-50.

Qvist J, Johansson SE, Johansson LM. Multivariate analyses of mortality from coronary heart disease due to biological and behavioural factors. Scand J Soc Med. 1996; 24(1): 67-76.

Rathmann W, Strassburger K, Heier M, Holle R, Thorand B, Giani G, Meisinger C. Incidence of Type 2 diabetes in the elderly German population and the effect of clinical and lifestyle risk factors: KORA S4/F4 cohort study. Diabet Med. 2009;26(12):1212–9.

Reis JP, Loria CM, Sorlie PD, Park Y, Hollenbeck A, Schatzkin A. Lifestyle factors and risk for new-onset diabetes: a population-based cohort study. Ann Intern Med. 2011;155(5):292–9.

Rintala P, Pukkala E, Läärä E, Vihko V. Physical activity and breast cancer risk among female physical education and language teachers: A 34-year follow-up. Int J Cancer. 2003;107(2):268-70.

Rintala PE, Pukkala E, Paakkulainen HT, Vihko VJ. Self-experienced physical workload and risk of breast cancer. Scand J Work Environ Health. 2002:158-62.

Rockhill B, Willett WC, Hunter DJ, Manson JE, Hankinson SE, Colditz GA. A prospective study of recreational physical activity and breast cancer risk. Arch Intern Med. 1999; 159(19): 2290-6.

Rosenberg L, Palmer JR, Bethea TN, Ban Y, Kipping-Ruane K, Adams-Campbell LL. A prospective study of physical activity and breast cancer incidence in African-American women. Cancer Epidemiology Biomarkers & Prevention. 2014 Nov 1;23(11):2522-31.

Rosengren A, Wilhelmsen L. Physical activity protects against coronary death and deaths from all causes in middle-aged men. Evidence from a 20-year follow-up of the primary prevention study in Göteborg. Ann Epidemiol. 1997; 7(1): 69-75.

Salonen JT, Puska P, Tuomilehto J. Physical activity and risk of myocardial infarction, cerebral stroke and death: a longitudinal study in Eastern Finland. Am J Epidemiol. 1982; 115(4): 526-37.

Sattelmair JR, Kurth T, Buring JE, Lee I-M. Physical activity and risk of stroke in women ;Stroke. 2010; 41(6): 1243-50.

Sesso HD, Paffenbarger RS Jr, Lee IM. Physical activity and breast cancer risk in the College Alumni Health Study (United States). Cancer Causes Control. 1998; 9(4): 433-9.

Sesso HD, Paffenbarger RS Jr, Lee IM. Physical activity and coronary heart disease in men: The Harvard Alumni Health Study. Circulation. 2000; 102(9): 975-80.

Severson RK, Nomura AM, Grove JS, Stemmermann GN. A prospective analysis of physical activity and cancer. Am J Epidemiol. 1989; 130(3): 522-9.

Shi L, Shu XO, Li H, Cai H, Liu Q, Zheng W, Xiang YB, Villegas R. Physical activity, smoking, and alcohol consumption in association with incidence of type 2 diabetes among middle-aged and elderly Chinese men. PLoS One. 2013; 8(11): e77919.

Siegel LC, Sesso HD, Bowman TS, Lee IM, Manson JE, Gaziano JM. Physical activity, body mass index, and diabetes risk in men: a prospective study. The American journal of medicine. 2009 Dec 31;122(12):1115-21.

Sieverdes JC, Sui X, Lee DC, Church TS, McClain A, Hand GA, Blair SN. Physical activity, cardiorespiratory fitness and the incidence of type 2 diabetes in a prospective study of men. Br J Sports Med. 2010;44(4):238–44.

Silvera SAN, Jain M, Howe GR, Miller AB, Rohan TE. Energy balance and breast cancer risk: a prospective cohort study. Breast Cancer Res Treat. 2006; 97(1): 97-106.

Simonsick EM, Lafferty ME, Phillips CL, Mendes de Leon CF, Kasl SV, Seeman TE, Fillenbaum G, Hebert P, Lemke JH. Risk due to inactivity in physically capable older adults. Am J Public Health. 1993; 83(10): 1443-50.

Slattery ML, Jacobs DR Jr, Nichaman MZ. Leisure time physical activity and coronary heart disease death. The US Railroad Study. Circulation. 1989; 79(2): 304-11.

Sobolski J, Kornitzer M, De Backer G, Dramaix M, Abramowicz M, Degre S, Denolin H. Protection against ischemic heart disease in the Belgian Physical Fitness Study: physical fitness rather than physical activity?. Am J Epidemiol. 1987; 125(4): 601-10.

Steenland K, Nowlin S, Palu S. Cancer incidence in the National Health and Nutrition Survey I. Follow-up data: diabetes, cholesterol, pulse and physical activity. Cancer Epidemiol Biomarkers Prev. 1995; 4(8): 807-11.

Steinbrecher A, Erber E, Grandinetti A, Nigg C, Kolonel LN, Maskarinec G. Physical activity and risk of type 2 diabetes among Native Hawaiians, Japanese Americans, and Caucasians: the Multiethnic Cohort. J Phys Act Health. 2012;9(5):634–41.

Steindorf K, Ritte R, Eomois P-P, Lukanova A, Tjonneland A, Johnsen NF, Overvad K, Østergaard JN, Clavel-Chapelon F, Fournier A, Dossus L, Teucher B, Rohrmann S, Boeing H, Wientzek A, Trichopoulou A, Karapetyan T, Trichopoulos D, Masala G, Berrino F, Mattiello A, Tumino R, Ricceri F, Quirós JR, Travier N, Sánchez M-J, Navarro C, Ardanaz E, Amiano P, Bueno-de-Mesquita HBA, van Duijnhoven F, Monninkhof E, May AM, Khaw K-T, Wareham N, Key TJ, Travis RC, Borch KB, Sund M, Andersson A, Fedirko V, Rinaldi S, Romieu I, Wahrendorf J, Riboli E, Kaaks R. Physical activity and risk of breast cancer overall and by hormone receptor status: the European prospective investigation into cancer and nutrition. Int J Cancer. 2013; 132(7): 1667-78.

Stringhini S, Tabak AG, Akbaraly TN, Sabia S, Shipley MJ, Marmot MG, Brunner EJ, Batty GD, Bovet P, Kivimaki M. Contribution of modifiable risk factors to social inequalities in type 2 diabetes: prospective Whitehall II cohort study. BMJ. 2012;345:e5452.

Sun F, Tao Q, Zhan S. An accurate risk score for estimation 5-year risk of type 2 diabetes based on a health screening population in Taiwan. Diabetes Res Clin Pract. 2009;85(2):228–34.

Sundquist K, Qvist J, Johansson S-E, Sundquist J. The long-term effect of physical activity on incidence of coronary heart disease: a 12-year follow-up study. Prev Med. 2005; 41(1): 219-25.

Suzuki R, Iwasaki M, Yamamoto S, Inoue M, Sasazuki S, Sawada N, Yamaji T, Shimazu T, Tsugane S, Japan Public Health Center-based Prospective Study Group. Leisure-time physical activity and breast cancer risk defined by estrogen and progesterone receptor status--the Japan Public Health Center-based Prospective Study. Prev Med. 2011; 52(3-4): 227-33.

Suzuki S, Kojima M, Tokudome S, Mori M, Sakauchi F, Fujino Y, Wakai K, Lin Y, Kikuchi S, Tamakoshi K, Yatsuya H, Tamakoshi A, Japan Collaborative Cohort Study Group. Effect of physical activity on breast cancer risk: findings of the Japan collaborative cohort study. Cancer Epidemiol Biomarkers Prev. 2008; 17(12): 3396-401.

Tamosiunas A, Luksiene D, Baceviciene M, Bernotiene G, Radisauskas R, Malinauskiene V, Kranciukaite-Butylkiniene D, Virviciute D, Peasey A, Bobak M. Health factors and risk of all-cause, cardiovascular, and coronary heart disease mortality: findings from the MONICA and HAPIEE studies in Lithuania. PloS one. 2014 Dec 5;9(12):e114283.

Tanasescu M, Leitzmann MF, Rimm EB, Willett WC, Stampfer MJ, Hu FB. Exercise type and intensity in relation to coronary heart disease in men. JAMA. 2002; 288(16): 1994-2000.

Thune I, Brenn T, Lund E, Gaard M. Physical activity and the risk of breast cancer. N Engl J Med. 1997; 336(18): 1269-75.

Thune I, Lund E. Physical activity and risk of colorectal cancer in men and women. Br J Cancer. 1996; 73(9): 1134-40.

Tsai AC, Lee SH. Determinants of new-onset diabetes in older adults–Results of a national cohort study. Clinical Nutrition. 2015 Oct 31;34(5):937-42.

Villegas R, Shu XO, Li H, Yang G, Matthews CE, Leitzmann M, Li Q, Cai H, Gao YT, Zheng W. Physical activity and the incidence of type 2 diabetes in the Shanghai women's health study. Int J Epidemiol. 2006; 35(6): 1553–62.

Wagner A, Simon C, Evans A, Ferrières J, Montaye M, Ducimetière P, Arveiler D. Physical activity and coronary event incidence in Northern Ireland and France: the Prospective Epidemiological Study of Myocardial Infarction (PRIME). Circulation. 2002; 105(19): 2247-52.

Waki K, Noda M, Sasaki S, Matsumura Y, Takahashi Y, Isogawa A, Ohashi Y, Kadowaki T, Tsugane S. Alcohol consumption and other risk factors for self-reported diabetes among middle-aged Japanese: a population-based prospective study in the JPHC study cohort I. Diabet Med. 2005;22(3):323–31.

Waller K, Kaprio J, Lehtovirta M, Silventoinen K, Koskenvuo M, Kujala UM. Leisure-time physical activity and type 2 diabetes during a 28 year follow-up in twins. Diabetologia. 2010; 53(12): 2531-7.

Wannamethee G, Shaper AG. Physical activity and stroke in British middle aged men. BMJ. 1992; 304(6827): 597-601.

Wannamethee SG, Shaper AG, Alberti KG. Physical activity, metabolic factors, and the incidence of coronary heart disease and type 2 diabetes. Arch Intern Med. 2000; 160(14): 2108-16.

Weinstein AR, Sesso HD, Lee I-M, Rexrode KM, Cook NR, Manson JE, Buring JE, Gaziano JM. The joint effects of physical activity and body mass index on coronary heart disease risk in women. Arch Intern Med. 2008; 168(8): 884-90.

Weinstein AR, Sesso HD, Lee IM, Cook NR, Manson JE, Buring JE, Gaziano JM. Relationship of physical activity vs body mass index with type 2 diabetes in women. JAMA. 2004; 292(10): 1188-94.

Weller I, Corey P. The impact of excluding non-leisure energy expenditure on the relation between physical activity and mortality in women. Epidemiology. 1998; 9(6): 632-5.

Willey JZ, Moon YP, Paik MC, Boden-Albala B, Sacco RL, Elkind MSV. Physical activity and risk of ischemic stroke in the Northern Manhattan Study. Neurology. 2009; 73(21): 1774-1779.

Williams PT, Thompson PD. Walking versus running for hypertension, cholesterol, and diabetes mellitus risk reduction. Arterioscler Thromb Vasc Biol. 2013;33(5):1085–91.

Wolin KY, Lee I-M, Colditz GA, Glynn RJ, Fuchs C, Giovannucci E. Leisure-time physical activity patterns and risk of colon cancer in women. Int J Cancer. 2007; 121(12): 2776-81.

Wyrwich KW, Wolinsky FD. Physical activity, disability, and the risk of hospitalization for breast cancer among older women. J Gerontol A Biol Sci Med Sci. 2000; 55(7): M418-421.

Wyshak G, Frisch RE. Breast cancer among former college athletes compared to non-athletes: a 15-year follow-up. Br J Cancer. 2000; 82(3): 726-30.

Xu F, Ware RS, Tse LA, Wang Y, Wang Z, Hong X, Chan EY, Dunstan DW, Owen N. Joint associations of physical activity and hypertension with the development of type 2 diabetes among urban men and women in Mainland China. PloS one. 2014 Feb 13;9(2):e88719.

Yu S, Yarnell JWG, Sweetnam PM, Murray L, Caerphilly study. What level of physical activity protects against premature cardiovascular death? The Caerphilly study. Heart. 2003; 89(5): 502-6.

Zhang Q, Zhou Y, Gao X, Wang C, Zhang S, Wang A, Li N, Bian L, Wu J, Jia Q, Wu S, Zhao X. Ideal cardiovascular health metrics and the risks of ischemic and intracerebral hemorrhagic stroke. Stroke. 2013; 44(9): 2451-2456

Table F. Continuous dose-response relationships between physical activity and breast cancer, colon cancer, diabetes, ischemic heart disease, and ischemic stroke

	Pooled RRs (UI)				
**Physical activity in MET-minutes per week**	**Breast cancer**	**Colon cancer**	**Diabetes**	**Ischemic heart disease**	**Ischemic stroke**
0	Reference	Reference	Reference	Reference	Reference
600	0.987(0.971, 1.003)	0.978(0.940, 1.016)	0.980(0.967, 0.996)	0.909(0.857, 0.964)	0.910(0.831, 1.000)
1,200	0.974(0.942, 1.005)	0.956(0.880, 1.032)	0.961(0.933, 0.992)	0.819(0.714, 0.928)	0.819(0.662, 1.001)
1,800	0.960(0.914, 1.008)	0.933(0.820, 1.048)	0.921(0.867, 0.985)	0.807(0.739, 0.886)	0.802(0.701, 0.916)
2,400	0.957(0.914, 1.001)	0.883(0.806, 0.958)	0.882(0.800, 0.977)	0.796(0.711, 0.895)	0.785(0.643, 0.946)
3,000	0.953(0.913, 0.994)	0.833(0.759, 0.907)	0.834(0.773, 0.904)	0.776(0.712, 0.844)	0.784(0.701, 0.888)
3,600	0.949(0.912, 0.990)	0.831(0.760, 0.903)	0.786(0.735, 0.841)	0.756(0.692, 0.816)	0.783(0.695, 0.884)
4,200	0.946(0.909, 0.985)	0.829(0.761, 0.898)	0.737(0.684, 0.794)	0.736(0.651, 0.822)	0.782(0.643, 0.934)
4,800	0.942(0.907, 0.982)	0.827(0.761, 0.894)	0.736(0.684, 0.792)	0.736(0.653, 0.818)	0.780(0.644, 0.926)
5,400	0.939(0.902, 0.979)	0.825(0.762, 0.890)	0.735(0.684, 0.790)	0.735(0.654, 0.815)	0.777(0.646, 0.919)
6,000	0.935(0.897, 0.979)	0.824(0.762, 0.887)	0.734(0.684, 0.786)	0.734(0.655, 0.812)	0.774(0.648, 0.913)
6,600	0.931(0.889, 0.978)	0.822(0.762, 0.884)	0.733(0.685, 0.785)	0.734(0.656, 0.809)	0.771(0.650, 0.905)
7,200	0.928(0.883, 0.978)	0.820(0.762, 0.880)	0.732(0.685, 0.782)	0.733(0.658, 0.806)	0.768(0.651, 0.898)
7,800	0.924(0.875, 0.979)	0.818(0.761, 0.877)	0.731(0.684, 0.780)	0.733(0.660, 0.803)	0.765(0.650, 0.891)
8,400	0.920(0.867, 0.979)	0.816(0.760, 0.873)	0.730(0.684, 0.779)	0.732(0.662, 0.801)	0.762(0.650, 0.884)
9,000	0.917(0.858, 0.979)	0.815(0.758, 0.871)	0.729(0.684, 0.777)	0.731(0.664, 0.799)	0.759(0.651, 0.878)
9,600	0.912(0.862, 0.970)	0.813(0.758, 0.868)	0.728(0.684, 0.776)	0.731(0.666, 0.796)	0.756(0.651, 0.872)
10,200	0.908(0.861, 0.961)	0.811(0.758, 0.864)	0.727(0.684, 0.774)	0.730(0.667, 0.794)	0.753(0.652, 0.865)
10,800	0.904(0.859, 0.952)	0.809(0.757, 0.862)	0.726(0.683, 0.772)	0.730(0.669, 0.792)	0.750(0.653, 0.858)
11,400	0.900(0.855, 0.948)	0.807(0.756, 0.860)	0.725(0.682, 0.771)	0.729(0.670, 0.789)	0.747(0.653, 0.852)
12,000	0.895(0.850, 0.946)	0.805(0.754, 0.858)	0.723(0.682, 0.771)	0.728(0.671, 0.787)	0.744(0.651, 0.846)
12,600	0.891(0.841, 0.946)	0.804(0.753, 0.857)	0.722(0.681, 0.768)	0.728(0.670, 0.787)	0.741(0.650, 0.841)
13,200	0.887(0.832, 0.949)	0.802(0.751, 0.856)	0.721(0.681, 0.767)	0.727(0.670, 0.785)	0.738(0.649, 0.833)
13,800	0.883(0.819, 0.950)	0.800(0.750, 0.854)	0.720(0.680, 0.765)	0.727(0.670, 0.784)	0.735(0.649, 0.828)
14,400	0.878(0.807, 0.952)	0.798(0.748, 0.851)	0.719(0.678, 0.764)	0.726(0.671, 0.782)	0.732(0.647, 0.825)
15,000	0.874(0.795, 0.957)	0.796(0.746, 0.850)	0.718(0.678, 0.762)	0.725(0.670, 0.781)	0.729(0.644, 0.820)
15,600	0.872(0.797, 0.952)	0.795(0.744, 0.849)	0.717(0.677, 0.761)	0.725(0.671, 0.781)	0.727(0.642, 0.815)
16,200	0.870(0.798, 0.946)	0.793(0.741, 0.849)	0.716(0.676, 0.760)	0.724(0.671, 0.779)	0.724(0.639, 0.810)
16,800	0.867(0.801, 0.941)	0.791(0.739, 0.848)	0.715(0.674, 0.759)	0.724(0.669, 0.779)	0.721(0.637, 0.806)
17,400	0.865(0.804, 0.934)	0.789(0.736, 0.848)	0.714(0.673, 0.759)	0.723(0.669, 0.781)	0.718(0.635, 0.803)
18,000	0.863(0.806, 0.927)	0.787(0.733, 0.847)	0.713(0.670, 0.759)	0.722(0.668, 0.780)	0.715(0.632, 0.798)
18,600	0.861(0.807, 0.919)	0.786(0.729, 0.846)	0.712(0.667, 0.759)	0.722(0.667, 0.781)	0.712(0.630, 0.793)
19,200	0.859(0.808, 0.914)	0.784(0.726, 0.846)	0.711(0.665, 0.758)	0.721(0.666, 0.781)	0.709(0.625, 0.792)
19,800	0.856(0.809, 0.910)	0.782(0.723, 0.845)	0.710(0.663, 0.758)	0.721(0.665, 0.781)	0.706(0.622, 0.790)
20,400	0.854(0.808, 0.907)	0.780(0.720, 0.845)	0.708(0.660, 0.759)	0.720(0.664, 0.782)	0.703(0.619, 0.790)

	Pooled RRs (UI)				
**Physical activity in MET-minutes per week**	**Breast cancer**	**Colon cancer**	**Diabetes**	**Ischemic heart disease**	**Ischemic stroke**
21,000	0.852(0.809, 0.900)	0.778(0.717, 0.846)	0.707(0.658, 0.759)	0.720(0.663, 0.782)	0.700(0.614, 0.787)
21,600	0.850(0.808, 0.896)	0.776(0.713, 0.846)	0.706(0.656, 0.759)	0.719(0.662, 0.782)	0.697(0.611, 0.787)
22,200	0.847(0.805, 0.893)	0.775(0.709, 0.847)	0.705(0.653, 0.759)	0.718(0.660, 0.783)	0.694(0.606, 0.787)
22,800	0.845(0.804, 0.892)	0.773(0.705, 0.848)	0.704(0.651, 0.760)	0.718(0.659, 0.784)	0.691(0.601, 0.785)
23,400	0.843(0.801, 0.888)	0.771(0.702, 0.849)	0.703(0.650, 0.760)	0.717(0.657, 0.786)	0.688(0.595, 0.783)
24,000	0.841(0.797, 0.888)	0.769(0.698, 0.849)	0.702(0.648, 0.760)	0.717(0.655, 0.787)	0.685(0.592, 0.781)
24,600	0.838(0.793, 0.889)	0.767(0.694, 0.850)	0.701(0.645, 0.760)	0.716(0.652, 0.788)	0.682(0.585, 0.779)
25,200	0.836(0.788, 0.891)	0.766(0.689, 0.850)	0.700(0.643, 0.760)	0.715(0.650, 0.790)	0.679(0.577, 0.779)
25,800	0.834(0.784, 0.892)	0.764(0.685, 0.850)	0.699(0.640, 0.760)	0.715(0.648, 0.791)	0.676(0.570, 0.777)
26,400	0.832(0.779, 0.893)	0.762(0.679, 0.851)	0.698(0.637, 0.760)	0.714(0.645, 0.792)	0.674(0.564, 0.778)
27,000	0.830(0.773, 0.893)	0.760(0.675, 0.853)	0.697(0.634, 0.760)	0.714(0.642, 0.794)	0.671(0.558, 0.779)
27,600	0.827(0.769, 0.894)	0.758(0.671, 0.854)	0.696(0.631, 0.761)	0.713(0.640, 0.797)	0.668(0.551, 0.781)
28,200	0.825(0.765, 0.897)	0.757(0.668, 0.856)	0.695(0.627, 0.762)	0.712(0.636, 0.798)	0.665(0.546, 0.782)
28,800	0.823(0.759, 0.900)	0.755(0.663, 0.857)	0.693(0.624, 0.763)	0.712(0.633, 0.800)	0.662(0.540, 0.783)
29,400	0.821(0.752, 0.903)	0.753(0.658, 0.857)	0.692(0.621, 0.764)	0.711(0.630, 0.801)	0.659(0.533, 0.785)
30,000	0.818(0.745, 0.905)	0.751(0.654, 0.856)	0.691(0.618, 0.765)	0.711(0.626, 0.802)	0.656(0.527, 0.788)
30,600	0.816(0.738, 0.906)	0.749(0.649, 0.858)	0.690(0.614, 0.766)	0.710(0.623, 0.803)	0.653(0.520, 0.789)

Table G. Sensitivity Analysis: relative risks for the associations between physical activity and breast cancer for studies that assessed physical activity quantitatively versus those that assessed it qualitatively

	**Pooled RRs (UI)**	
**Physical activity in MET-minutes per week**	**Studies that assessed physical activity quantitatively**	**Studies that assessed physical activity qualitatively**
<600	Reference	Reference
600 – 3,999	0.975(0.930, 1.024)	0.958(0.912, 1.006)
4,000 – 7,999	0.948(0.896, 1.004)	0.930(0.870, 0.988)
≥ 8,000	0.871(0.821, 0.923)	0.858(0.805, 0.914)
